# Artificial Intelligence Meets Whole Slide Images: Deep Learning Model Shapes an Immune-Hot Tumor and Guides Precision Therapy in Bladder Cancer

**DOI:** 10.1155/2022/8213321

**Published:** 2022-09-19

**Authors:** Yiheng Jiang, Shengbo Huang, Xinqing Zhu, Liang Cheng, Wenlong Liu, Qiwei Chen, Deyong Yang

**Affiliations:** ^1^Department of Urology, First Affiliated Hospital of Dalian Medical University, Dalian 116021, China; ^2^School of Information and Communication Engineering, Dalian University of Technology, Dalian, China; ^3^Department of Pathology and Laboratory Medicine, Warren Alpert Medical School of Brown University, Lifespan Academic Medical Center, Providence, RI 02903, USA; ^4^School of Information Science and Technology, Dalian Maritime University, 116000 Dalian City, Liaoning Province, China

## Abstract

**Background:**

To construct and validate a deep learning cluster from whole slide images (WSI) for depicting the immunophenotypes and functional heterogeneity of the tumor microenvironment (TME) in patients with bladder cancer (BLCA) and to explore an artificial intelligence (AI) score to explore the underlying biological pathways in the developed WSI cluster.

**Methods:**

In this study, the WSI cluster was constructed based on a deep learning procedure. Further rerecognition of TME features in pathological images was applied based on a neural network. Then, we integrated the TCGA cohort and several external testing cohorts to explore and validate this novel WSI cluster and a corresponding quantitative indicator, the AI score. Finally, correlations between the AI cluster (AI score) and classical BLCA molecular subtypes, immunophenotypes, functional heterogeneity, and potential therapeutic method in BLCA were assessed.

**Results:**

The WSI cluster was identified associated with clinical survival (*P* < 0.001) and was proved as an independent predictor (*P* = 0.031), which could also predict the immunology and the clinical significance of BLCA. Rerecognition of pathological images established a robust 3-year survival prediction model (with an average classification accuracy of 86%, AUC of 0.95) for BLCA patients combining TME features and clinical features. In addition, an AI score was constructed to quantify the underlying logic of the WSI cluster (AUC = 0.838). Finally, we hypothesized that high AI score shapes an immune-hot TME in BLCA. Thus, treatment options including immune checkpoint blockade (ICB), chemotherapy, and ERBB therapy can be used for the treatment of BLCA patients in WSI cluster1 (high AI score subtype).

**Conclusions:**

In general, we showed that deep learning can predict prognosis and may aid in the precision medicine for BLCA directly from H&E histology, which is more economical and efficient.

## 1. Introduction

Bladder cancer (BLCA) is one of the worldwide most common urinary malignancies, approximately 83,730 incident cases were reported in the USA in 2021 [1]. BLCA was proved progress along two different pathways, which is posing two quite different challenges for therapeutic opportunities: nonmuscle invasive cancers, not immediately life-threatening, but they are prone to recurrence and require costly lifelong monitoring [2]. In contrast, muscle-invasive bladder cancers (MIBC) tend to rapidly progress and have a bad prognosis [3].

MIBC is defined as a heterogeneous disease at the molecular level due to its genomic instability and high mutation rate [4]. A previous study proposed several instinct BLCA molecular subtypes based on RNA-Seq data. Subtypes with the most scientific consensus are MDA subtype [5] and UNC subtype [6]; on the basis of these two subtypes, other classification systems have identified similar groups [7, 8]. However, these methods are difficult to be implemented in clinical setting, the main limiting factor is the requirement of sample size for RNA-Seq data analysis, and high accuracy analysis of RNA-Seq data relies on sufficient sample size. Therefore, a large number of patient samples need to be collected, which is difficult in clinical implementation. Thus, a high need for a faster and more economic procedure is in urgent [9, 10]. One possible solution is the application of deep learning model to predict clinical-related parameters; the advent of deep learning and thousands of hematoxylin and eosin- (HE-) stained slides has provided new opportunities to reexamine classic methods of diagnosis and prediction of patient outcomes [11–14].

Although deployed in a growing number of studies, approaches to elucidate how deep learning algorithms make decisions tend to lack [15]. However, this is critical because these models will only be widely applied and supported if there is a way to understand the underlying decision process [16]. In our study, deep learning was used to accurately identify the BLCA subtypes from WSI of HE-stained slides downloaded from TCGA to establish a novel molecular subtype of BLCA and correlated them in TME, comprehensive genomic, immunophenotypes, and clinical outcomes [17]. In general, our study demonstrated that this novel WSI cluster can provide a good classifier for full-resolution microscopic pathological image learning, even if it only uses the entire image label for training.

## 2. Methods

### 2.1. Data Retrieval and Preprocessing

#### 2.1.1. Training Cohorts

Our data set comes from the TCGA database, which provides an online platform for the research community to upload, search, view, and download cancer-related data [18]. After filtering the genomic and clinicopathological data, a total of 363 TCGA-BLCA samples were selected. All the selected patients were diagnosed with muscle-invasive bladder cancer (MIBC). Furthermore, after removing low-quality pathological images, 435 digitized HE stained histopathological WSI of these 363 patients were then downloaded for molecular subtype recognition.

#### 2.1.2. External Testing Cohorts

Two GSE cohorts (GSE32894 and GSE13507) with detailed survival data of 389 BLCA samples were gathered from Gene Expression Omnibus (GEO) and integrated to a GEO-metatesting cohort for external validation. IMvigor210 cohort, with 348 BLCA samples received anti-PD-L1 immunotherapy, was obtained in the IMvigor210CoreBiologies R package. In addition, the E-MTAB-4321 cohort (with detailed survival data of 476 BLCA samples) was gathered from the European Molecular Biology Laboratory database. Besides, three immunotherapy-related cohorts (GSE135222, GSE100797, and Nathanson2017 pre) were gathered from the GEO database and TIDE website.

### 2.2. Construction of Deep Learning Signature and WSI Cluster

To cluster patients, we chose WSI as the clustering sources and wanted to extract the overall image features of them. First, we downsampled the WSI at a reduction of 0.15625x by openslide library and cropped 512 × 512 pixel (removing the background around) for feature extraction. Furthermore, we clustered all the patients into 2-5 groups using different kinds of features by different common clustering methods, obtaining different clustering results. The final type of features was extracted from the last fully connected layer of inception V3 model which was pretrained by ImageNet and was of 2048 dimensions. We then selected the number of blocks for patients empirically; too many blocks for one patient may lead to overfitting and too few may make the feature extraction inadequate. Therefore, the block number were determined by multiple quantitative experiments of prepreparation. The significance of different clustering classes is to find the boundaries of medicine; 2-5 is the number of classes that may be meaningful in clinical. Doctors are more likely to get valuable conclusions and valid applications based on the class. After our ablation experiment, the following three clustering methods are finally determined to have the best experimental effect: Gaussian mixture model clustering, mini batch *K*-means clustering, and hierarchical clustering. We then clustered all the images into several groups, from 2 to 5, using the three mentioned methods. Results which had less than 5 images in a group were dropped.

From the above, we obtained 5 distinct classification model and 70 specific groups, survival analysis was then conducted using R package survminer. Among them, the three-classification model based on mini batch *K*-means clustering model was identified associated with the best prognosis for OS among all groups (*P* = 0.005). Therefore, we determined this three-classification model as WSI cluster. Due to the survival analysis, we found that the groups clustered by mini batch *K*-means using features extracted by the neural network had meaningful performance. In order to capture the image features of these clusters, we trained a new classification model using the above clusters as labels and then visualized the class activation maps by Grad-CAM and summarized the interpretable features by experts. For classification, we used the same multiplier of whole slide images as when clustering and trained an inception V3 model by transfer learning using 6-fold cross-validation. We did 3 independent experiments, each reaching comparable conclusions. The optimizer we used was SGD with learning rate of 0.001, and the loss function was cross-entropy.

### 2.3. Rerecognition of Tumor Microenvironment Features by Artificial Intelligence

The rerecognition of TME features based on WSI is all based on the neural network. Considering the amount of data in the data set, the parameters after ImageNet training were used as pretraining parameters for transfer learning, only the last fully connected layer was trained. In order to ensure the accuracy of the input features of the prognostic prediction model, we excluded the cases with missing clinical data and the surviving cases with less than 3 years of follow-up, and pathologists then browsed the block classification results of each case and excluded the cases with an overall block prediction accuracy of less than 80%, leaving 120 cases. Furthermore, in the ablation experiment of various features, the hidden layer design is adjusted according to the number of experimental input features, so as to obtain the optimal model of current features and evaluate the effectiveness of the feature for prognostic prediction, see Supplementary Information for details (Table S1-S2).

### 2.4. Development of AI Score

We then developed a signature score (AI score) to explore underlying logistic of the WSI cluster. We followed the methods of Hu et al. [19]. First of all, univariate Cox regression analysis and lasso regression were conducted on the differential expressed genes (DEGs) between WSI cohorts to identify prognostic AI gene signature. Then, principal component 1 was gathered from principal component analysis (PCA) on those above prognostic DEGs; this method had advantage of focusing the score on the set with the largest block of well correlated (or anticorrelated) genes in the set while downweighting contributions from genes that do not track with other set members. The process to establish the AI score was similar to that in previous studies [20–28].

(1) $$\begin{array}{c} AI\text{ score}=\sum \text{P}\text{C}1i, \end{array}$$

where *i* means the expression of prognostic DEGs in the WSI cluster.

For all external testing BLCA cohorts, we, respectively, calculated the AI score based on the prognostic AI gene signature. Furthermore, we evaluated the AI score in pan-cancer cohort to ensure the comparability of the analyses.

### 2.5. Exploration of TME Underlying WSI Cluster and AI Cluster

From the above, we then constructed and validated the AI cluster. Specifically, patients were classified into high and low AI score groups based on the median AI score. The Kaplan-Meier method was then applied to explore the prognostic significance of the AI score. Based on the gene expression profile, the potential biological pathways underlying WSI cluster and AI cluster were identified [29]. First, differentially expressed genes (DEGs) between every subgroup stratified by the WSI cluster were identified using DESeq2 R package. Furthermore, functional enrichment analysis (GO and KEGG) were applied based on the clusterProfiler R package [30]. Besides, immunophenoscore (IPS) was calculated to evaluate the immune status [31, 32]. In addition, ESTIMATE, CIBERSORT [33], and MCP-counter [34] R package were used to evaluate the immune cell infiltration populations; samples with *P* < 0.05 were reserved for further analysis. Similarly, according to the previous research [35], we constructed immune cell signatures for further assessing the landscape of tumor microenvironment phenotypes in WSI cluster and AI cluster. Besides, scores of each step in cancer-immunity cycle was calculated with ssGSEA [36], see Supplementary Information for details (Section II Supplementary Results).

### 2.6. Evaluation of WSI Cluster-Associated Immune Cell Infiltration, Clinical Features, and Classic Molecular Subtypes

First, the association between immune cell infiltration populations and WSI cluster was evaluated using Kruskal-Wallis statistic. Next, we explore the distribution of clinical features and molecular characteristics of the WSI cluster. Finally, multivariate Cox regression analysis was used to depict the prognostic value of WSI cluster with overall survival as the endpoint, see Supplementary Information for details (Figure S13-S17).

Besides, R packages ConsensusMIBC and BLCAsubtyping were used to calculate multiple classic molecular subtype characteristics in WSI cluster, including CIT, Lund, MDA, TCGA, Baylor, UNC, and Consensus subtypes. Besides, we classified these seven classic BLCA molecular subtypes into two major subtypes (basal and luminal) basing on the previous study. Thereafter, ROC curves were drawn to explore the predictive ability of AI score for these classic molecular subtypes. The results were confirmed reproducible in the external testing cohort.

### 2.7. Depiction of Classical Molecular Subtype-Specific Signatures and Efficacy Prediction of Several Treatments

Previous researches have concluded twelve distinct molecular subtype-specific signatures [37]. Besides, critical targeted therapy-related signatures and radiotherapy-related signatures were also summarized [38]. Additionally, the mutation proportions of multiple critical genes (including ERBB2, ERCC2, ATM, RB1 and FANCC, which were proved predict the therapeutic response to neoadjuvant chemotherapy) was calculated. Furthermore, Mariathasan et al. developed nineteen gene signatures associated with the therapeutic response to atezolizumab (an anti-PD-L1 drug) in BLCA [39].

In this study, we collected the enrichment scores of these above signatures in BLCA [40]. Then, we identified the role of AI score in predicting the response of these therapeutic opportunities. Furthermore, potential BLCA-related drugs along with corresponding drug-target genes were gathered from the DrugBank database for further analysis [41]. Additionally, we checked for MSigDB database to explore several biological pathways (including hallmark, oncogenic, and KEGG pathways) [42]. To further explore the biological processes and KEGG pathways in AI clusters, we optimize the algorithm based on R package clusterProfiler; the new procedure was as follows: [1] Differential analysis was conducted between the high AI score subtype (*n* = 181) and the low AI score (*n* = 181). An absolute value of log2 (fold change) > 1 combined with the false discovery rate (FDR) adjusted *P* value < 0.05 was selected as the threshold for cutoff value [2]. Then, 3,792 differential expressed genes (DEGs), including 3,198 upregulated expression (UE) and 544 downregulated expression (DE), were filtered out from TCGA cohort in high AI score subtype vs. low AI score subtype [3]. Furthermore, GO (including biological processes, cellular component, and molecular function) and KEGG pathway analyses were performed based on the DEGs separately.

### 2.8. Statistical Analysis

Statistical analysis was applied using R (version 4.0.3) and R Bioconductor packages. Continuous and ordered categorization variables were evaluated with Student's *t*-test, Kruskal-Wallis test, and Wilcoxon test. Pearson *χ*^2^ test or Fisher exact test were used to compare disordered categorization variables. A permutation test was performed to compare the mutation frequencies between clusters. Correlation matrices are created using Pearson or Spearman correlation. The pROC and timeROC R package were called to plot receiver operating characteristic (ROC) curve and to calculate time-dependent area under the ROC curve (AUC). Kaplan-Meier method was used for survival analysis. All tests are bidirectional, and *P* < 0.05 is considered significant unless otherwise noted. FDR calibration is used for multiple tests to reduce false positives.

## 3. Results

### 3.1. Depicting the WSI Clusters

**Figure 1:**
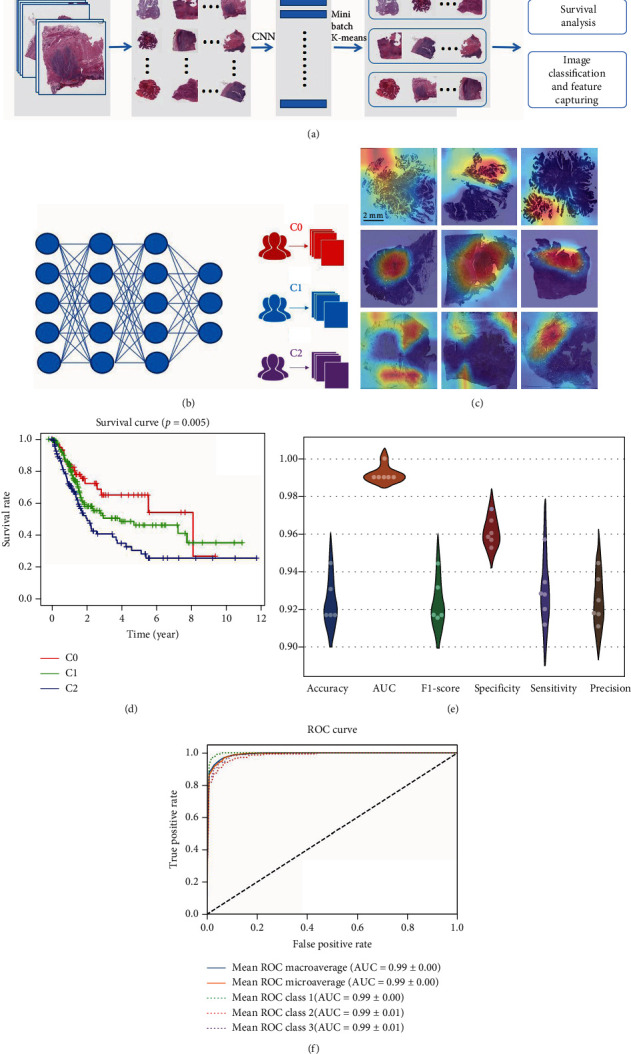
Overview of the deep learning model. (a) The overall flow chart of developing WSI cluster. (b) The model training for WSI cluster at tie level. (c) Whole slide images of patients with each WSI subtype in TCGA cohort. (d) Survival analysis of WSI cluster (e) The performance of 6-fold cross validation. (f) The average ROC curve of 6-fold cross-validation.

We obtained 435 digitized H&E-stained histopathology WSI of 363 patients from TCGA database. For these slides, in order to cluster them with whole image features instead of detailed cell and tissue features, we chose to use the downsampled images instead of tiled patches. We downsampled all the slides at a reduction of 0.15625x by openslide library and cropped 512 × 512 pixel (removing the background around) for features extraction. We tried three types of features, e.g., 512-dimension histogram features, flatten features, and features extracted from pretrained Inception V3 model (Figure 1(b)). Then, we used mini batch *K*-means, hierarchical clustering, and Gaussian mixture mode (GMM) to cluster patients into 2-5 groups. After feature extraction and clustering, we use Kaplan-Meier for survival assessment and found that the three groups clustered by mini batch *K*-means using features of the neural network got the best performance (Figure 1(c)).

Besides, to capture the image features among these three groups, we did transfer learning with Inception V3 model to classify them and then used Grad-CAM to visualize the important areas. For 6-fold cross-validation, we got an average classification accuracy of 92.38%, AUC of 0.99 (Figure 1(f)), *F*1 score of 0.9167, specificity of 0.9568, sensitivity of 0.9115, and precision of 0.9117 (Figure 1(e)). The workflow of constructing WSI clusters is shown in Figure 1(a).

### 3.2. Rerecognition of TME Features in WSI Based on Machine Learning

**Figure 2:**
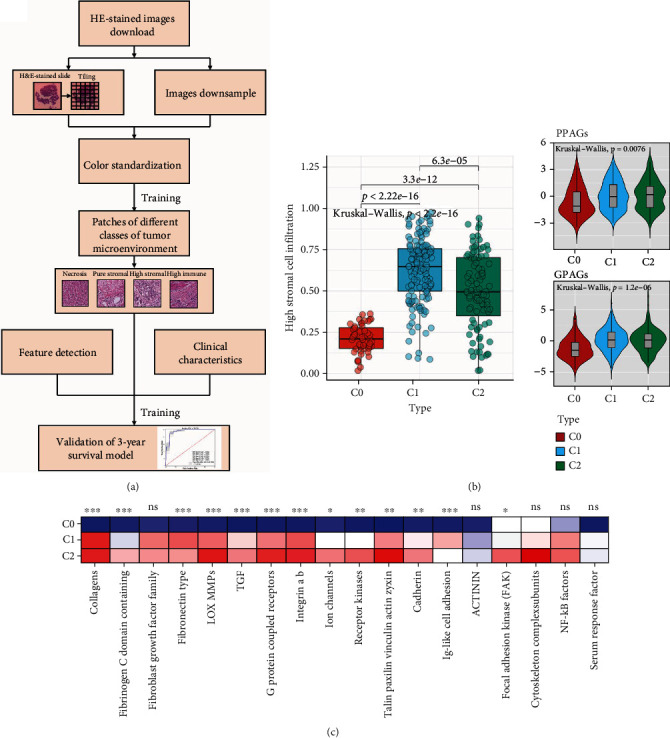
Rerecognition of tumor microenvironment features by artificial intelligence. (a) The flow chart of the rerecognition algorithm. (b) The differences in the enrichment scores of stromal cell infiltration degree between WSI cluster. (c) The differences in the expression of stromal cell-related pathways between WSI subgroups.

The workflow of the machine learning procedure to rerecognition of TME features in WSI was shown in (Figure 2(a)). The WSI within TME features were defined as pure stromal infiltration, necrosis, high immune high stromal infiltration, high immune low stromal infiltration, low immune high stromal infiltration, and low immune low stromal infiltration according to three pathologists for classification (Figure 2(b)). Four neural networks were then built overall to complete the hierarchical classification. For the labeled 12,000 blocks, the data set was divided into the training cohort, test cohort, and validation cohort by 8 : 1 : 1 randomly. The models were evaluated by accuracy, AUC, specificity, and sensitivity, and the performance of each network is shown in Table S3. In addition, the 3-year survival prediction model was constructed based on the overall image features, TME features, and clinical features. The network design and experimental results of the specific backpropagation neural network (BPNN) were shown in Table S4. Among them, three types of features showed the best results (AUC = 0.95, accuracy = 86.00%, specificity = 0.8353, and sensitivity = 0.8923). Collectively, in this research, based on the machine learning for semiquantitative assessment of TME features in WSI, we established a robust 3-year survival prediction model that incorporates pathological image features, TME features, and clinical features in the BLCA cohort, which confirmed the accuracy and effectiveness of machine learning applied to WSI.

### 3.3. Clinical Parameters, Molecular Characteristics, and Prognosis of the WSI Clusters

Clinical-related parameters and molecular characteristics, classified by WSI clusters, was, respectively, calculated and summarized in (Table S2). Compared with C0 and C1, the median survival age of C2 is slightly higher (Figure 1(d)). Besides, C2 revealed worse stages among three AI subtypes. In addition, C0 showed more luminal type and less basal type than C1 and C2 in terms of MDA subtype, which presented with better pathological differentiation. In addition, we found that the WSI cluster showed a significantly prognostic value in the BLCA cohort.

### 3.4. Biological Functional Analyses

To explore the underlying differential biological functional procedures among WSI clusters, GO and KEGG analyses were applied on DEGs separately. (Figure S1). As shown in Figure 1(d), C0 and C2 revealed great difference in survival analysis, we found that DEGs between C0 and C2 were significantly enriched in extracellular matrix/structure organization and collagen-containing extracellular matrix, indicating that difference between C0 and C2 were mainly reflected in the extracellular matrix, possibly altering cellular activities such as adhesion and migration [43]. Besides, KEGG analysis also revealed that PI3K-Akt pathway was significantly enriched, which was confirmed play a critical role in control the normal progression of cell cycle [44] and the initiation and progression of bladder cancer [45] in previous researches. Besides, compared with C0 and C1, GO terms of C2 were primarily enriched in T cell activation, which is important in localized human BLCA treatment with BCG [46]. In addition, KEGG terms of C2 showed that inflammation and immune-related pathways were significantly enriched as expected, including cytokine-cytokine receptor interaction.

### 3.5. Immune Characterization and Mutation of WSI Cluster

**Figure 3:**
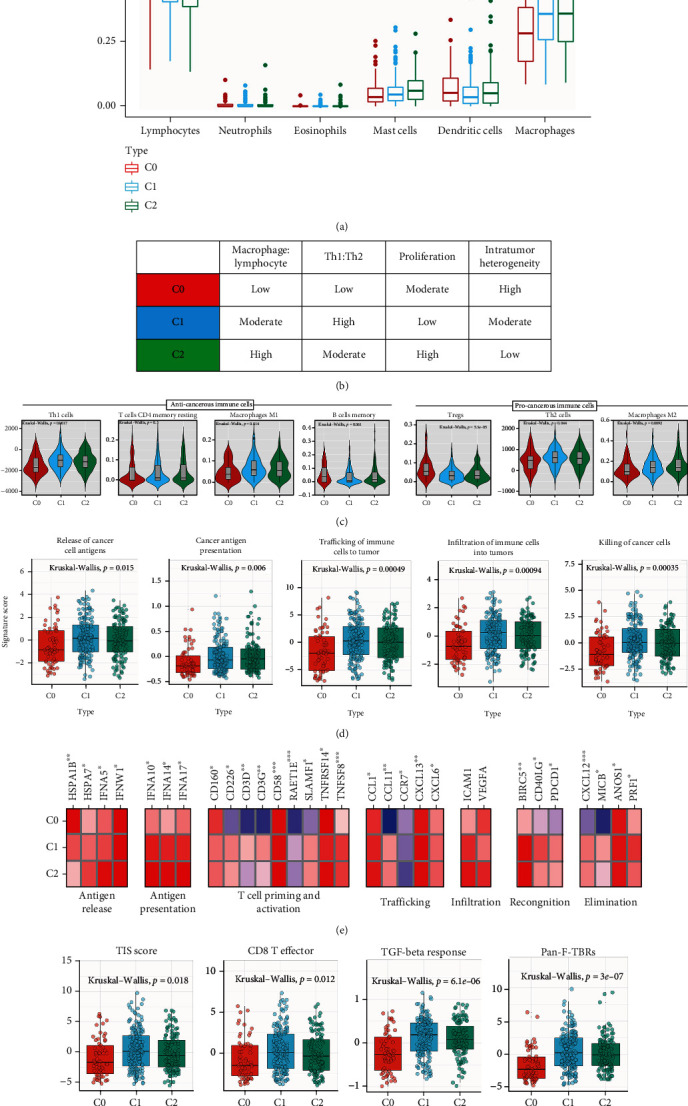
The WSI cluster correlated with immune phenotypes in the TCGA-BLCA cohort. (a) The proportion of major classes of immune cells (from CIBERSORT) within the leukocyte compartment for different WSI subgroups. (b) Key characteristics of WSI subtypes. (c) Values of key immune characteristics by WSI subtype. (d) Differences in activities of the cancer immunity cycles between WSI subgroups. (e) The differences in the expression of cancer immune cycle effector genes between WSI subgroups. (f) The differences in the enrichment scores of positive ICB response-related signatures between WSI cluster.

First, IPS were calculated as an immune activation indicator among WSI subtypes. C1 showed a higher IPS *z*-score than C0 and C2, suggesting its higher immunogenic [32] and a different TME among WSI clusters. Furthermore, C1 showed higher effector cell scores (*P* = 0.0066) and lower suppressor cell scores (*P* = 0.0017). Besides, we found that no differences in the immune checkpoint category and antigen presentation across WSI clusters. (Figure 3 (d)).

Then, ESTIMATE method was applied in WSI clusters to explore their stromal and immune cell infiltration. C0 revealed a lower immune score and the lowest stromal score. In contrast, C1 showed more abundance of stromal scores (*P* < 0.001) and immune scores (*P* = 0.0091), in agreement with the IPS results (Figure S2A).

To further elucidate this issue in immune cells infiltration, Xcell method was also used to evaluate the association between WSI cluster and immune phenotypes (Figure S2B and C). First, the highest abundance of resting and activated CD4+ T memory cells, memory B cells and Treg cells was confirmed in C0 (*P* < 0.001). However, the least abundance of Th1 and Th2 cells, M1 and M2 macrophages presented in C0, which is related to the lowest expression of macrophages and mast cells. In comparison, C1 exhibited the highest density of Th1 and Th2 cells, M1 and M2 macrophages, and the lowest density of memory B cells, Treg cells, and dendritic cells. The lymphocytes and mast cell expression of C1 were in the middle among the three WSI clusters (Figures 3(b) and 3(c)). Interestingly, the highest abundance of mast cells was confirmed in C2 compared with C0 and C1 (Figure 3(f)), accompanied by the lowest abundance of lymphocytes.

Then, we collected the 13 most significantly mutated genes basing on previous research [4]. As shown in (Figure S2D), the C0 group was significantly enrich in KDM6A (35%), ARID1A (31%), and FGFR3 mutations (21%). These tumor-associated FGFR3 activation signatures suggest that C0 may respond to FGFR inhibitors [47]. C1 was mainly characterized by enrichment of PIK3CA (25%) and RB1 mutations (21%), PIK3CA mutation test helps to distinguish between tumors (indeed supported by PI3K activation) and cancers characterized by participation in other signaling pathways, while PIK3CA inhibitors show satisfactory activity in breast cancer [48]. In additional, C2 shows higher STAG2 mutant rates, which is reportedly correlated with poor prognosis in BLCA [49]; STAG2 has also been shown to regulate the cell cycle progression of bladder cancer cells [50], suggesting that the C2 group may respond to chemotherapy. Besides, in this study, we observed that p53/cell cycle regulation (mutations occurred in 66.13% of cases in C0, 71.26% in C1, and 67.84% in C2) and PI(3)K signal transduction (mutations occurred in 64.52% of cases in C0, 57.47% in C1, and 53.91% in C2) were significantly altered in the WSI clusters; other signaling pathways, including RTK, TP53, Notch, Myc, Hippo, Nrf2 and TGF*β* pathways, did not change significantly among these three subtypes (*P* > 0.05), which is consistent with previous research [7].

### 3.6. C1 Is More Suitable for ICB Therapy

Analysis of the clinical characteristics showed that the C0 group had lower T stage and pathological grade, which was associated with its higher OS (Table S1). Besides, pathway and functional enrichment analysis showed significant differences in inflammatory and immune-related pathways between C1 and C2 subtypes (Figure S1). Therefore, we suggest that there are differences in reactivity to immune checkpoint inhibitors between these two subtypes. In our cohort, C1 shows a significantly higher infiltration of CD8 effector T cells, along with the presence of elevated pan-fibroblast TGF-*β* response signature (Pan-F-TBRS), which may indicate the better OS of this subtype relate to its effect on stromal cells [21] (Figure 3(f)).

Biomarkers of T cell–inflamed tumor microenvironment mainly include tumor inflammation signature (TIS) [16, 18] and the expression of multiple inhibitory receptors (IR), including PDCD1. In this study, TIS (*P* = 0.018) was observed higher abundance in C1 subtype; besides, expression of PDCD1 was identified upregulated in C1 compared with the other subtypes, suggesting that anti-PD1 treatments in C1 may response (Figure 3(f)). These results indicated that patients in C1 may exhibit a stronger response to checkpoint blockade immunotherapy than patients in other subtypes. Besides, C1 also showed higher EMT scores (*P* < 0.001), which was proved valuable in predicting patients who may suitable for ICB therapy and antiangiogenesis drugs.

### 3.7. Depiction of AI Signature, AI Score, AI Cluster, and Their Functional Analyses

**Figure 4:**
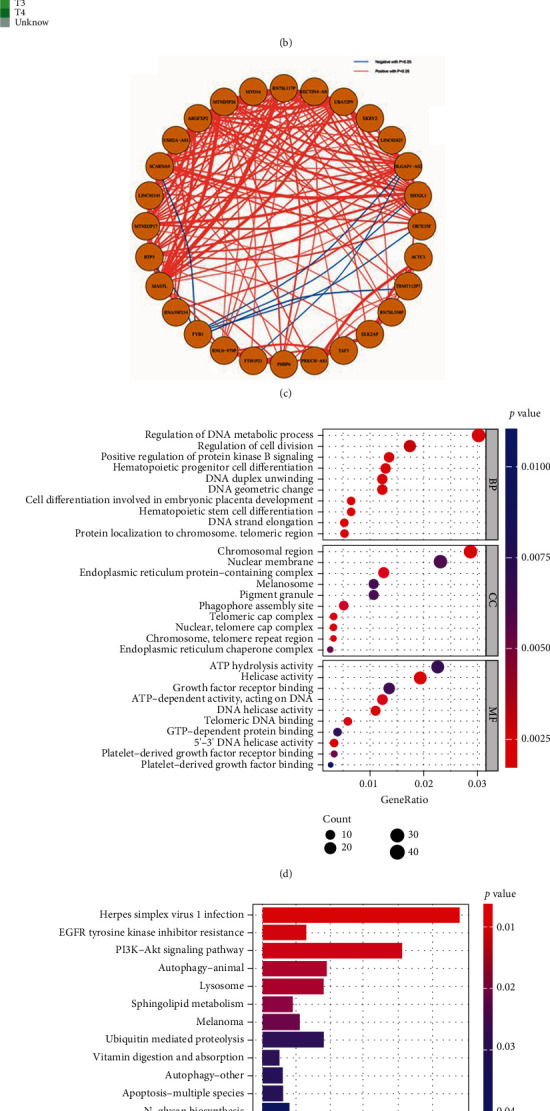
Developing the AI gene signature, AI score and their functional analyses in the TCGA-BLCA cohort. (a) The flow chart of the AI score algorithm. (b) The heatmap shows the expression of 28 optimal genes (AI signature) between AI clusters. (c) The volcano plot shows the 28 optimal genes, and the correlation between these genes. (d) Gene Ontology (GO) analysis of the AI gene signature. (e) Kyoto Encyclopedia of Genes and Genomes (KEGG) analysis of the AI gene signature. (f) Survival analysis of the AI score cluster. (g) The differences in the AI score between the AI subgroups. (h) ROC curve showed the accuracy of the AI score in predicting prognosis in the TCGA-BLCA cohort.

Furthermore, to depict the molecular differences between WSI clusters, we developed an AI score to quantify WSI cluster. Figure S3 displays the AI score algorithm. First, 1,369 DEGs were identified between each two WSI clusters as mentioned above (Figure S1). Within the DEGs, 802 DEGs had prognostic value. To avoid overfitting, lasso regression analysis was conducted to identify 28 optimal genes with minimal lambda (0.0741) in WSI cluster (Figure S12). Next, PCA was performed on the selected prognostic DEGs to calculate the AI score. Finally, we divided the WSI cluster into a high AI score subtype (*n* = 181) and a low AI score subtype (*n* = 181), K-M survival analysis shows that low AI score subtype had a better prognosis than high AI score subtype (Figure 4(f)). Interestingly, we found that most of these genes are enriched in the high AI score group. Besides, we observed that AI score could quantify the characteristics of WSI clusters effectively (Figures 4(g) and 4(h)).

Next, we summarized the differences between the AI clusters in mutation landscape and biological pathways. To explore the potential biological pathways regarding AI score, we, respectively, performed GSVA in the high AI score group and low AI score group. Results showed that between these two AI groups, several immune-related biological pathways (including proliferation-relevant, DDR-relevant, and cell component-relevant pathways) were enriched higher in the high AI score subtype. Along with the biological pathways, a majority of immune-related hallmark (Figure S4A) pathways were also highly enriched in the high AI score subtype. Furthermore, Figure S5 performed the biological functional analyses based on the expression of DEGs in the high AI score subtype; as expected, PI3K-Akt pathway was significantly enriched in the high AI score subtype, while the Notch pathway was negative correlated with the high AI score subtype. These results revalidated the previous GSVA analysis, indicating that high AI score subtype may benefit more from the ICB therapy. TP53 and RB1 mutations were proved lead to the instability of genomic and benefit the response of ICB therapy in previous study [51]. In our cohort, TP53 (52% vs. 44%) and RB1 (25% vs. 12%) mutation rates were observed significantly abundance in the high AI score subtype (Figure S4C and D). Besides, several oncogenic pathways were also highly enriched in the high AI score subtype (Figure S4B). These results demonstrated that high AI score subtype (WSI cluster C1) may have a better prognosis during ICB therapy.

### 3.8. High AI Score Shaped an Immune-Hot Tumor in BLCA

**Figure 5:**
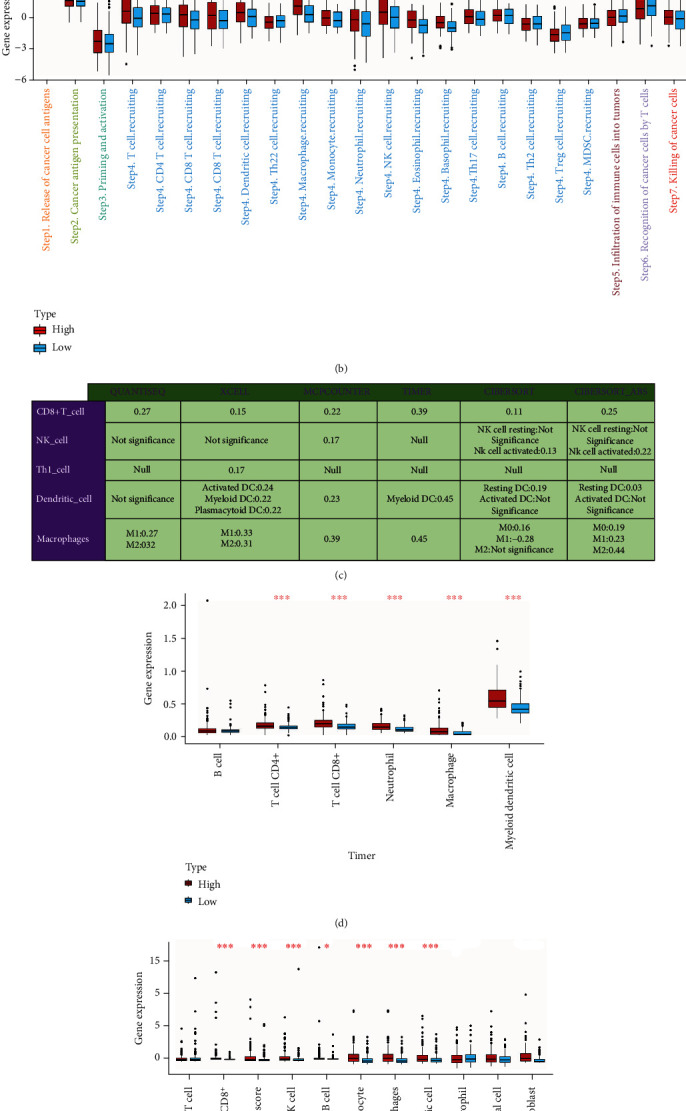
AI score shapes an inflamed TME in BLCA. (a) Differences in the expression of 122 immunomodulators (chemokines, receptors, MHC, and immunostimulators) between AI clusters in BLCA. (b) Differences in the various steps of the cancer immunity cycle between AI clusters. (c) Correlation between AI score and the infiltration levels of five types of TIICs (CD8+ T cells, NK cells, macrophages, Th1 cells, and dendritic cells), which were calculated using six independent algorithms. (d, e) The differences in infiltration levels of TIICs between AI clusters in the TIMER and MCP-counter algorithms. The asterisks indicate a statistically significant p value calculated using the Mann–Whitney *U* or *t*-test (^∗^*P* < 0.05, ^∗∗^*P* < 0.01, and ^∗∗∗^*P* < 0.001). (f) The differences in the expression of effector genes of several anticancer TIICs (including CD8+ T cells, NK cells, macrophages, Th1 cells, and dendritic cells) between AI subtypes.

**Figure 6:**
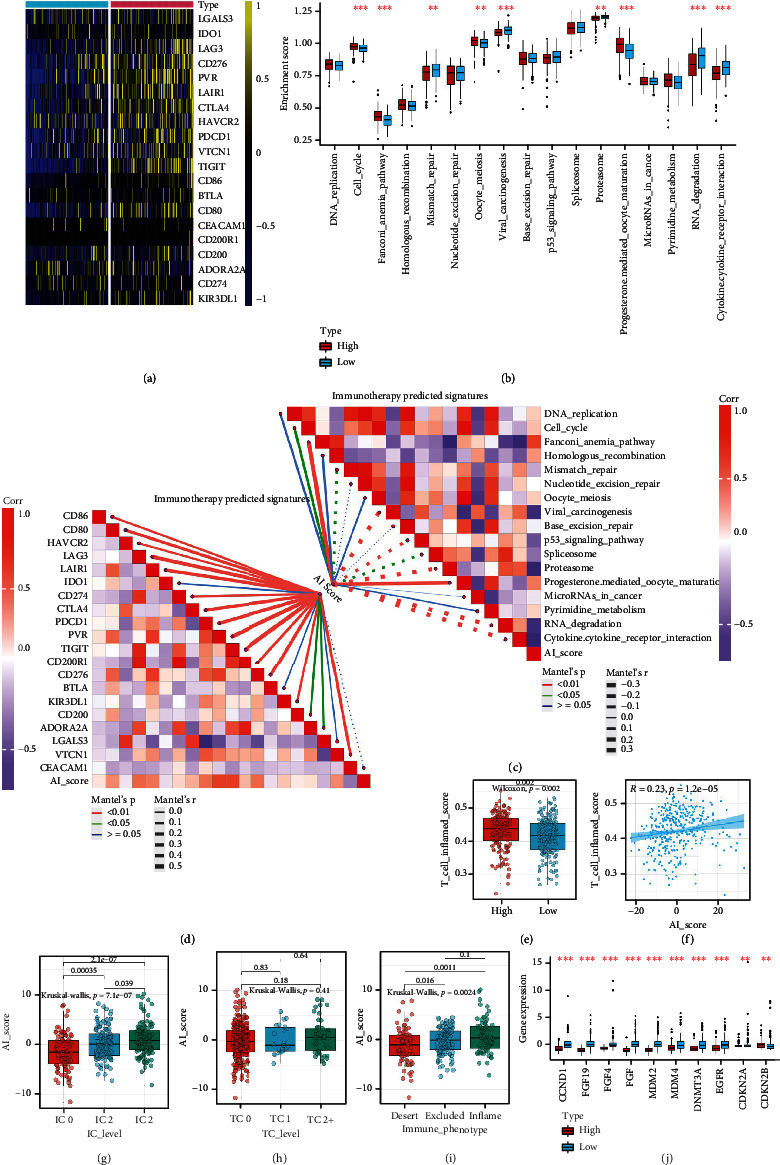
The AI clusters and AI score correlated with predictors of ICB response in the TCGA-BLCA cohort. (a) The differences in the expression of 22 immune checkpoints between AI clusters. (b) The differences in the enrichment scores of positive ICB response-related signatures between AI clusters. (c, d) The upper right part indicates the correlations between AI score and the enrichment scores of positive ICB response-related signatures; the lower left part shows the correlations between AI score and the expression of 22 immune checkpoints. (e, f) The differences and correlations between AI score and TIS. (g, h) Differences in the PD-L1 expression on tumor cells, and the PD-L1 expression on immune cells between AI clusters in the IMvigor210 cohort. (i) Expression of AI score in all three phenotypes in the IMvigor210 cohort. (j) The difference in mRNA expression of hyperprogression-associated genes between AI clusters. The asterisks indicate a statistically significant *P* value calculated using the Mann–Whitney *U* or *t*-test (^∗^*P* < 0.05, ^∗∗^*P* < 0.01, and ^∗∗∗^*P* < 0.001).

In our cohort, AI score was found to be positively correlated with a majority of immunomodulators (Figure 5(a)). MHC-related molecules were significantly upregulated in high AI score subtype, indicating that high AI score subtype shows higher ability in antigen presentation and processing. Besides, critical chemokine ligand family members (including CXCL1, CXCL2, CXCL5, CXCL8, and CXCL11), which involved in tumor growth regulation and metastasis and mediated the enrichment of CD8+ T cells into the TME, were upregulated in the high AI score subtype. In addition, AI score was found positively correlated with a majority of critical steps of the cancer immunity cycle (Figure 5(b)). As a result, AI score was positively correlated with a majority of anticancer TIICs and their corresponding effector genes, which were validated using six independent algorithms (Figure 5(f)). It is reported that expression of immune checkpoint inhibitors was high in inflamed TME; consistently, in our cohort, AI score was found significantly positively correlated with several ICB-related genes including PD-L1, CTLA4, and PD-1 (Figure 6(a)).

Besides, these results were validated in external testing cohorts. For example, AI score was identified higher in inflamed phenotypes, and the TC2 and IC2 groups in the IMvigor210 cohort (Figures 6(g)–6(i)). Besides, AI score was proved positively associated with multiple immunomodulators, TIIC effector genes, and ICB-related genes in GSE32894, GSE13507, and E-MTAB-4321 cohort (Figure S9A-E, Figure S10A-E). In addition, the pan-cancer analyses of the AI score also confirmed the prognostic value of the AI score in 33 types of cancers (Figure S11A-D).

Collectively, AI score strongly correlated with the development of an immune-hot tumor in BLCA. Multivariate Cox analysis also showed that AI score was an independent prognostic factor in BLCA.

### 3.9. AI Score Predicted Immunotherapy Response and Hyperprogression of ICB in BLCA

As mentioned above, high AI score defines an inflamed TME, patients in high AI score subtype should have a higher response to ICB. As expected, we found that AI score was positively associated with the enrichment scores of most immunotherapy-related signatures (Figure 6(b)), which was validated in three external cohorts (Figure S9E, Figure S10E). Besides, we also assessed the association between AI score and several immune signature in three external cohorts. Results of the validation cohort also demonstrated that higher AI score showed higher immune signatures and predicted a higher therapeutic response to immunotherapy (Figure S9B and C, Figure S10B and C). In addition, expression of ICB-associated hyperprogression is lower in high AI score subtype. (Figure 6(j)).

**Figure 7:**
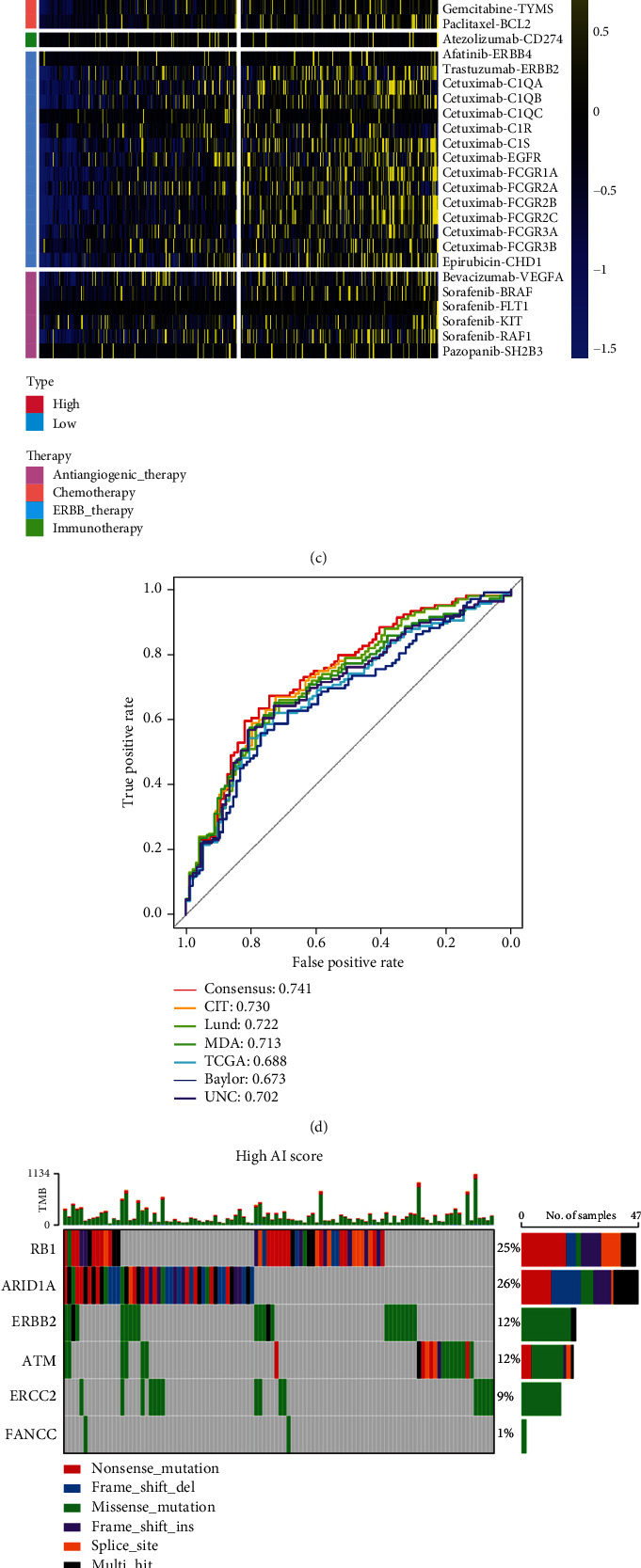
AI clusters predicted classical molecular subtypes and therapeutic opportunities in the TCGA-BLCA cohort. (a, b) The correlations between AI clusters and seven classical molecular subtype classifications. (c) The correlations between the AI cluster and the enrichment scores of several therapeutic signatures, such as targeted therapy and radiotherapy. (d) ROC curves showed the accuracy of the AI score in predicting classical molecular subtypes. (e, f) The overall mutation rates of neoadjuvant chemotherapy-related genes in the AI cluster subgroups. (g) The difference on the therapeutic sensitivities of six chemotherapy drugs, including cisplatin, docetaxel, gemcitabine, paclitaxel, bleomycin, and camptothecin.

Besides, we compared the correlationships between AI scores and ICB therapeutic response predictors. Most of the ICB predictors were upregulated in high AI score subtype (Figure 6(a)). On the other hand, the signature scores of potential ICB response-related predictors and the TIS scores were significantly higher in high AI score subtype than low AI score subtype (Figure 7(e)). Therefore, high AI score subtype may be more sensitive to ICB.

In summary, patients in the high AI score cohort may benefit more from ICB therapy as they showed higher effective responsive to ICB and lower hyperprogression.

### 3.10. AI Score Predicted Classic Molecular Subtypes and Therapeutic Opportunities

Previous research demonstrated that basal-type BLCA tend to show the highest infiltration abundance of immune cell and the best therapeutic response to pembrolizumab [52]. In our cohort, BLCA with higher AI score was more likely to be categorized as the basal-subtype among the classic molecular subtypes. Additionally, the signature scores of luminal differentiation, myofibroblasts, and smooth muscle were higher in the low AI score subtype. On the other hand, the signature scores of basal, urothelial, and EMT differentiation are higher in high AI score subtype (Figure 7(a)). These results revalidated that AI score was positively associated with the response to ICB therapy. Besides, to enhance the differences and correlations between AI clusters and seven classical molecular subtypes, we further applied correlation analysis based on the R package GGally. The coincident and exclusive associations across the signature scores of seven classical molecular subtypes and AI scores from the high and low AI score subtype were analyzed, respectively, in which red represents the high AI score subtype while blue represents the low AI score subtype. As expected, the signature score of EMT differential (*P* < 0.05, corr = 0.067) and basal differential (*P* < 0.05, corr = 0.079) were positively correlated with AI score, while the signature score of Luminal differential (*P* < 0.001, corr = −0.412), myofibroblasts (*P* < 0.001, corr = −0.476), and smooth muscle differential (*P* < 0.05, corr = 0.079) were negatively correlated with AI score (*P* < 0.001, corr = −0.276), which is consistent with the differential analysis. These results revalidated the previous results, indicating that AI score was positively associated with the response to ICB therapy (Figure 7(a)). Additionally, these outcomes were validated using three external cohorts (Figure S7A and D).

Furthermore, the ROC curves showed the high accuracy of AI score (ranging from 0.67 to 0.74) in predicting classical molecular subtypes (Figure 7(d)). These results were validated in external testing cohorts (Figure S7C and D). Besides, we also explored the role of AI score in identifying different classic molecular subtypes, in line with the results mentioned above, basal-subtype showed the highest AI score, while luminal subtypes showed the lowest AI score (Figure S6B). Collectively, AI score could reflect the potential biological characteristics of the rare molecular subtypes, which cover the shortage of the binary molecular subtype system.

Molecular subtype of BLCA can also predict the therapeutic response of multiple treatment opportunities, including chemotherapy and radiotherapy [53]. Basal-subtype tumors were considered benefit more from neoadjuvant chemotherapy. As expected, basal-subtype signatures (including mutation rates of RB1, ERBB2, and FANCC) were significantly higher in the high AI score (Figure 7(e)). Besides, high AI score subtype indicated a significantly higher response to several therapeutic opportunities basing on the results from the DrugBank database (Figure 7(c)). For example, high AI score subtype showed more sensitive reaction to chemotherapeutic drugs, including cisplatin, paclitaxel, gemcitabine, docetaxel, etoposide, camptothecin, and bleomycin (Figure 7(d)). In general, treatment options including ERBB therapy, ICB therapy, and chemotherapy (neoadjuvant or adjuvant) can be used for the patients in the high AI score subtype.

In contrast, low AI score subtype showed more similarity with the luminal-subtype (Figure 7(a)). As a reason, ICB therapy, radiotherapy, and chemotherapy may all unsuitable for patients in low AI score subtype. As mentioned above, low AI score shapes an immune-cold tumor in BLCA; therefore, targeted therapy blocking immunosuppressive oncogenic pathways can be used for the treatment of patients in low AI score subtype (Figure S8A-D). On the other hand, we demonstrated that patients in low AI score subtype may benefit more from antiangiogenic therapy (Figure S8C, Figure S8B, and D).

## 4. Discussion

We developed a deep learning procedure to integrate information from both WSI and genomic characteristics into a single framework to predict outcomes. The prognostic accuracy of our approach was systematically evaluated based on gene expression, functional enrichment analysis, molecular characteristics, TME, and clinical parameters. Remarkably, we were able to achieve almost a 1.0 AUC for detecting the classification of MIBC molecular subtypes. In addition, rerecognition of WSI based on machine learning confirmed the predictive ability of WSI subtypes. Further analysis demonstrated that WSI subtype was characterized by distinctive mutations, mRNA expression profiles, tumor microenvironment, composition of tumor-infiltrating immune cells, and cancer-immunity cycle. These findings suggest that molecular-targeted therapy with cytokine and/or chemokine antagonists works well in patients in C0. Besides, combined therapeutic opportunities of antiangiogenic therapy and ICB therapy may improve outcomes of patients in C1, while patients in subtype C2 may benefit more from chemotherapy.

Then, we further developed an AI score to quantify the WSI clusters. First, we confirmed that high AI score shaped an immune-hot tumor in BLCA basing on the positive correlation between AI score and immunological status of tumor microenvironment. On the other hand, we demonstrated that AI score could predict the therapeutic response of ICB, chemotherapy, radiotherapy, and targeted therapy.

A rapidly growing number of works are now trying to obtain clinically information, such as molecular characteristics or tumor microenvironment information, from traditional histopathological morphology using complex deep learning algorithms [11, 13, 23]. However, although results are encouraging, there are still some vital discussion points, including that performance measurement reports are often incomplete and lack a method to elucidate how deep learning algorithms reach their decisions. So far, the accuracy of BLCA molecular subtypes has not attained a satisfactory level for further clinical application, even though in binary classification models [54]. In this study, two deep learning methods were applied including neural networks and convolutional, combining these two techniques to establish a model that is applicable to different size images and may suitable for the digital pathology workflow assessment of BLCA tumor samples. In addition, combining recent deep learning algorithm with transfer learning-based approaches achieves an AUC of almost 1.0. Lastly, we built CAM and “reverse-engineered” the histomorphological criteria most related to our deep learning model to construct specific BLCA molecular subtypes [55]. Collectively, we confirmed specific histomorphological characteristics responsible for different BLCA subtypes.

TME has been proved to be associated with prognosis in BLCA [24]. TME not only interacts with tumor cells to benefit them to proliferate and keep them from metastasis and apoptosis but also plays a crucial role in therapeutic opportunities predicting [25, 26]. In this study, the WSI clusters and AI clusters (AI score) revealed various biological pathways of the TME in BLCA. Function enrichment analysis demonstrated that DEGs between WSI clusters were significantly enriched in immune-related pathways, such as cytokine/chemokine signaling pathways. Besides, AI score was positively associated with most of the immunomodulators, such as CXCL1, CXCL2, and CXCR5, which were proved vital for the TIICs infiltration. Meanwhile, the AI score was positively correlated with the activities of several steps of cancer immunity cycles, and multiple anticancer-related TIICs, including CD8+ T cells and NK cells. In general, high AI score (WSI cluster C1) indicated an immune-hot tumor characterized by high potential anticancer immunity. A previous study has proved that an inflamed TME is more sensitive to ICB [27]. Identically, in this research, we found that the AI score was positively related to ICB-related signatures, UCB-related genes, and TIS scores [56]. Besides, we revalidated these results in the IMvigor210 cohort [28]. Furthermore, we found that AI score was negatively correlated with the incidence of ICB-related hyperprogression. Collectively, AI score may be a potential predictor of ICB therapy in BLCA.

In addition, AI score showed robust potential in predicting multiple other therapeutic opportunities, including chemotherapy (neoadjuvant), targeted therapy, and radiotherapy. High AI score subtype is more likely basal-subtype characterized by the higher basal differentiation. Consistently, in the high AI score subtype, mutation rate of RB1 was significantly higher, which indicated that the high AI score group (WSI cluster C1) may be more sensitive to chemotherapy (neoadjuvant). Meanwhile, the high AI score subtype (WSI cluster C1) was demonstrated sensitive to ERBB therapy and radiotherapy. In addition, different immune-related oncogenic pathways were enriched in high AI score subtype (WSI cluster C1). Therefore, patients in high AI score subtype may benefit more from targeting these pathways. A previous study proved that these immune-inhibited oncogenic pathways may lead to an inflamed TME [38]. In line with this, we found that the high AI score shapes an immune-hot tumor and showed an inflamed phenotype characterized by higher level of anticancer immunity infiltration, which indicated that high AI score subtype (WSI cluster C1) may benefit more from ICB therapy. In general, our deep learning model showed a robust ability to accurately distinct classic molecular subtypes and guide precision therapy in the BLCA cohort.

Although ICB therapy is undoubtedly one of the most effective immunotherapy strategies in BLCA potential therapeutic opportunities, there remain nonnegligible problems, including growing adverse events, possible low response rate, and inescapable acquired resistance. Researches have now demonstrated that combination of antiangiogenesis therapy and ICB therapy can not only reprogram the irresponsive TME to an immune responsive microenvironment but also enhance the anti-cancer effect [38]. Tian et al. [57] defined good-prognosis angiogenesis (GPAGs) and poor-prognosis angiogenesis genes (PPAGs). Among them, GPAGs are highly relevant to cell-cell adhesion and proliferation of smooth muscle cells, while PPAGs are mostly relevant to ECM decomposition and hypoxia. In this study, C1 is characterized by higher PPAGs and lower GPAGs, suggesting tumor angiogenesis was present in C1 (Figure 3(b)). In addition, our study on angiogenesis signaling pathway demonstrated that C1 was highly enriched in LOX MMP pathway, which partially explained the relationship between angiogenesis and OS of C1 (Figure 3(c)). At present, the combination between ICB therapy and anti-VEGF antibodies (bevacizumab in combination with intravenous) [58], anti-VEGFR antibodies (ramucirumab in combination with pembrolizumab) [59], and VEGFR TKI axitinib in combination with pembrolizumab) [60] tend to show more clinical benefits than ICB therapy or antiangiogenic therapy and homogenous combination therapy. In conclusion, we suggest that patients in C1 may respond better to the use of antiangiogenic agents in combination with ICIs.

In clinical, whole-transcriptome sequencing data tend to obtain difficultly because its higher cost. In addition, flow cytometry is difficult to detect all infiltrated immune cells and stromal cells in the TME, requiring complex protocols and high-quality BLCA tissues. Therefore, we focus on obtaining information about molecular subtypes and precision therapeutic opportunities of BLCA in a more convenient way. We demonstrate how AI can propose a convolutional neural network-based strategy by recognizing features in pathological images. The development of cheaper and more powerful techniques has made it possible to train larger and more complex neural networks.

## 5. Conclusion

We developed and validated a robust pathological image-based deep learning model to identify three reproducible WSI subtypes of BLCA, which shows reliable generalizability to predict the clinical outcomes of BLCA patients. Deep learning rerecognition supports our analysis of TME. The AI score was conducted to quantify WSI clusters, identify distinct classic BLCA molecular subtypes, and stratify precision therapeutic opportunities in BLCA. Besides, we found that AI score shapes an immune-hot tumor in BLCA and is able to predict response to ICB therapy and the potential BLCA molecular subtype.

## Data Availability

The original data presented in this study can be found in online repositories. Contact the corresponding authors for further inquiries. For readers to repeat the analysis easily, essential scripts are available on the GitHub website (https://github.com/YihengJiang0912/AI-meets-WSI).

## Supplementary Materials

Supplementary Materials Figure S1: differential expression analysis and functional analysis of WSI clusters. The left parts show differential expression analysis in WSI clusters; the middle parts show GO analysis based on the separate DEGs between WSI clusters; and the right parts show KEGG analysis based on the separate DEGs between WSI clusters. (A) 530 DEGs was found in C0 vs. C1. (B) 497 DEGs was found in C0 vs. C2. (C) 342 DEGs was found in C1 vs. C2. Figure S2: immune characterization and mutation of WSI cluster. (A) Immunophenoscore (IPS) across the WSI clusters. (B, C) Immune cell infiltration in WSI cluster using Xcell algorithm. (D) Mutation profile of WSI cluster in BLCA main dysregulated pathways. (E) Cox regression analysis of immune checkpoints in WSI clusters. Figure S3: construction and validation of AI cluster. The workflow of constructing AI clusters and the AI score. Figure S4: functional analyses of AI clusters. (A) The differences in hallmark pathways between the AI cluster. (B) The differences in oncogenic pathways between the AI clusters. (C, D) The differences in mutational profiles between AI clusters. Figure S5: the differences in GO and KEGG pathways between AI clusters. (A) GO enrichment of the DEGs between AI clusters shows the activation status of biological pathways in different AI cluster. (B) KEGG pathway enrichment based on the DEGs between AI clusters. The blue bar shows the enrichment analysis results based on down-regulated genes in high AI score subtype, representing the negative correlated biological processes and KEGG pathways with the high AI scores, while the red bar shows the enrichment analysis results based on upregulated genes in high AI score subtype, representing the positive correlated biological processes and KEGG pathways with the high AI scores. Figure S6: (A) the proportions of every subgroup in seven classic subtype systems. (B) The distribution of AI score among different subgroups in all molecular subtype systems. Figure S7: AI score predicted classical molecular subtypes in two external validation BLCA cohorts. (A) The correlations between AI score and seven classical molecular subtype classifications in the GEO BLCA cohorts (GSE48075 and GSE32894). (B) ROC curves showed the accuracy of the AI score in predicting seven classical molecular subtypes in the GEO BLCA cohorts. (C) The correlations between AI score and seven classical molecular subtype classifications in the E-MTAB-4321 cohort. (D) ROC curves showed the accuracy of AI score in predicting seven classical molecular subtypes in the E-MTAB-4321 cohort. Figure S8: AI score predicted therapeutic opportunities in two external validation BLCA cohorts. (A, C) The correlations between AI score and the BLCA-related drug-target genes were screened from the DrugBank database; the upper part shows the correlation between AI score and the E-MTAB-4321 cohort; the lower part shows the correlation between AI score and the GEO BLCA cohorts (GSE48075 and GSE32894). (B, D) The correlations between AI score and the enrichment scores of several therapeutic signatures, such as targeted therapy and radiotherapy, in the E-MTAB-4321 cohort. Figure S9: AI score correlated with immune phenotypes and ICB response in the GEO BLCA cohorts (GSE48075, GSE32894). (A) Survival analysis of AI score clusters. (B) The differences in 122 immunomodulators between AI score clusters. (C) The differences in the expression of effector genes of five anticancer TIICs (including CD8+ T cells, NK cells, macrophages, Th1 cells, and dendritic cells) between the AI score clusters. (D) The correlations between AI score and TIS. (E) The lower left part shows the correlations between AI score and the expression of 22 immune checkpoints; the upper right part shows the correlations between AI score and the enrichment scores of positive ICB response-related signatures. Figure S10: AI score correlated with immune phenotypes and ICB response in the E-MTAB-4321 cohort. (A) Survival analysis of AI score clusters. (B) The differences in 122 immunomodulators between AI score clusters. (C) The differences in the expression of effector genes of five anticancer TIICs (including CD8+ T cells, NK cells, macrophages, Th1 cells, and dendritic cells) between AI score clusters. (D) The correlations between AI score and TIS. (E) The lower left part shows the correlations between AI score and the expression of 22 immune checkpoints; the upper right part shows the correlations between AI score and the enrichment scores of positive ICB response-related signatures. Figure S11: Pan-cancer analyses of the AI score and AI gene signature. (A) The prognostic analyses of AI score across cancers using a univariate Cox regression model. A hazard ratio > 1 indicated a risk factor, and a hazard ratio < 1 represented a protective factor. (B) Correlations between AI score and potential immune checkpoints. (C) The correlations between AI score and TMB in all cancers. (G) The correlations between AI score and MSI across cancers. The asterisks indicate a significant statistical p-value calculated with Spearman correlation analysis (^∗^*P* < 0.05, ^∗∗^*P* < 0.01, and ^∗∗∗^*P* < 0.001). Figure S12: identification of AI score candidate member. (A) LASSO coefficient profiles of 802 prognostic DEGs between WSI clusters. The coefficient profile plot was developed against the log (lambda) sequence. (B) Cross-validation for turning parameter selection via minimum criteria in the LASSO regression model. Two dotted vertical lines were plotted at the optimal values using the minimum criteria. Optimal RNAs with the best discriminative capability (28 in number) were selected for developing the AI score. Figure S13: Survival analysis of binary classification model identified by deep learning procedure. Mini batch *K*-means, hierarchical clustering, and Gaussian mixture mode (GMM) were, respectively, conducted for clustering (^∗^*P* < 0.05, ^∗∗^*P* < 0.01, and ^∗∗∗^*P* < 0.001). Figure S14: survival analysis of triple-classification model identified by deep learning procedure. Mini batch *K*-means, hierarchical clustering, and Gaussian mixture mode (GMM) were, respectively, conducted for clustering. The model marked by red rectangle was selected to develop WSI cluster (^∗^*P* < 0.05, ^∗∗^*P* < 0.01, and ^∗∗∗^*P* < 0.001). Figure S15: survival analysis of four-classification model identified by deep learning procedure. Mini batch *K*-means, hierarchical clustering, and Gaussian mixture mode (GMM) were, respectively, conducted for clustering (^∗^*P* < 0.05, ^∗∗^*P* < 0.01, and ^∗∗∗^*P* < 0.001). Figure S16: survival analysis of five-classification model identified by deep learning procedure. Mini batch *K*-means, hierarchical clustering, and Gaussian mixture mode (GMM) were, respectively, conducted for clustering (^∗^*P* < 0.05, ^∗∗^*P* < 0.01, and ^∗∗∗^*P* < 0.001). Figure S17: survival analysis of seven-classification model identified by deep learning procedure. Mini batch *K*-means, hierarchical clustering, and Gaussian mixture mode (GMM) were, respectively, conducted for clustering (^∗^*P* < 0.05, ^∗∗^*P* < 0.01, and ^∗∗∗^*P* < 0.001). Table S1: univariable and multivariable analyses for overall survival in patients with BLCA. Table S2: clinicopathological characteristics of patients with BLCA in TCGA. Table S3: performance of patch classification models. Table S4: ablation experiments of 3-year prediction features.
