# Supplementary material for: Artificial Intelligence Meets Whole Slide Images: Deep Learning Model Shapes an Immune-Hot Tumor and Guides Precision Therapy in Bladder Cancer
Source: J Oncol. 2022 Sep 19;2022:8213321. doi: 10.1155/2022/8213321 (PMC9553530; doi:10.1155/2022/8213321)
Supplement: Supplementary Materials — Figure S1: differential expression analysis and functional analysis of WSI clusters. The left parts show differential expression analysis in WSI clusters; the middle parts show GO analysis based on the separate DEGs between WSI clusters; and the right parts show KEGG analysis based on the separate DEGs between WSI clusters. (A) 530 DEGs was found in C0 vs. C1. (B) 497 DEGs was found in C0 vs. C2. (C) 342 DEGs was found in C1 vs. C2. Figure S2: immune characterization and mutation of WSI cluster. (A) Immunophenoscore (IPS) across the WSI clusters. (B, C) Immune cell infiltration in WSI cluster using Xcell algorithm. (D) Mutation profile of WSI cluster in BLCA main dysregulated pathways. (E) Cox regression analysis of immune checkpoints in WSI clusters. Figure S3: construction and validation of AI cluster. The workflow of constructing AI clusters and the AI score. Figure S4: functional analyses of AI clusters. (A) The differences in hallmark pathways between the AI cluster. (B) The differences in oncogenic pathways between the AI clusters. (C, D) The differences in mutational profiles between AI clusters. Figure S5: the differences in GO and KEGG pathways between AI clusters. (A) GO enrichment of the DEGs between AI clusters shows the activation status of biological pathways in different AI cluster. (B) KEGG pathway enrichment based on the DEGs between AI clusters. The blue bar shows the enrichment analysis results based on down-regulated genes in high AI score subtype, representing the negative correlated biological processes and KEGG pathways with the high AI scores, while the red bar shows the enrichment analysis results based on upregulated genes in high AI score subtype, representing the positive correlated biological processes and KEGG pathways with the high AI scores. Figure S6: (A) the proportions of every subgroup in seven classic subtype systems. (B) The distribution of AI score among different subgroups in all molecular subtype systems. Figure S7: AI score [file 8213321.f1.zip › Supplementary (3).docx]

***Supplementary Material***

Artificial Intelligence meets Whole Slide Images: Deep learning model shapes an immune-hot tumor and guides precision therapy in bladder cancer

1. **Supplementary Methods**
2. **Supplementary Results**
3. **Supplementary Tables and Figures**

Contents

1. **Supplementary Methods**

1. Study design

1. **Supplementary Results**

1. Potential biological pathways, immunophenoscore, immunogenicity, and immune cell infiltration populations between any two WSI clusters

1.1 C0 vs C1

1.2 C0 vs C2

1.3 C1 vs C2

1. **Supplementary Figures and Tables**

Figure S1. Differential expression analysis and Functional analysis of WSI clusters.

Figure S2. Immune characterization and Mutation of WSI cluster.

Figure S3. Construction and validation of AI cluster.

Figure S4. Functional analyses of AI clusters.

Figure S5. The differences in KEGG pathways between AI clusters.

Figure S6. The proportions of every subgroup in seven classic subtype systems.

Figure S7. AI score predicted classical molecular subtypes in two external validation BLCA cohorts.

Figure S8. AI score predicted therapeutic opportunities in two external validation BLCA cohorts.

Figure S9. AI score correlated with immune phenotypes and ICB response in the GEO BLCA cohorts (GSE48075, GSE32894).

Figure S10. AI score correlated with immune phenotypes and ICB response in the E-MTAB-4321 cohort.

Figure S11. Pan-cancer analyses of the AI score and AI gene signature.

Figure S12. Identification of AI score candidate member.

Figure S13. Survival analysis of Binary-Classification Model identified by deep learning procedure.

Figure S14. Survival analysis of Triple-Classification Model identified by deep learning procedure.

Figure S15. Survival analysis of Four-Classification Model identified by deep learning procedure.

Figure S16. Survival analysis of Five-Classification Model identified by deep learning procedure.

Figure S17. Survival analysis of Seven-Classification Model identified by deep learning procedure.

Table S1A. Univariable and multivariable analyses for overall survival in patients with BLCA.

Table S1B. Clinicopathological characteristics of patients with BLCA in TCGA.

Table S2A. Performance of patch classification models.

Table S2B. Ablation experiments of 3-year prediction features.

**I Supplementary Methods**

**1. Study design**

In this study, the whole slide images (WSI) cluster was constructed based on a deep learning cohort (n = 367). Further re-recognition of tumor microenvironment (TME) features in pathological images was applied based on a neural network. Then, we integrated the TCGA cohort and multiple external bladder cancer (BLCA) cohorts to explore and validate this novel WSI cluster and a corresponding quantitative indicator, the AI score. Finally, correlations between the AI cluster (AI score) and classical molecular subtypes, immunophenotypes, clinical outcomes, and potential therapeutic opportunities in BLCA were assessed. The WSI cluster was identified as associated with survival (P < 0.001) and was an independent predictor (P =0.031), which could also predict the clinical outcomes and the TME immune characteristics of BLCA. Re-recognition of pathological images established a robust 3-year survival prediction model (with an average classification accuracy of 86%, AUC of 0.95) for BLCA patients combining TME features and clinical features. In addition, an AI score was constructed to quantify the underlying logic of the WSI cluster (AUC = 0.838). We hypothesized that high AI score designs an inflamed TME in BLCA based on the evidence that AI score positively correlated with immunomodulators, immune checkpoints, cancer immunity cycles, and TIICs. Thus, treatment options including immune checkpoint blockade (ICB), neoadjuvant or adjuvant chemotherapy, and ERBB therapy can be used, either alone or in combination, for the treatment of BLCA patients in WSI cluster1 (with high AI score).

**II Supplementary Results**

**1. Potential biological pathways, immunophenoscore, immunogenicity, and immune cell infiltration populations between any two WSI clusters**

**1.1 C0 vs C1**

The WSI cluster C0 and C1 represented distinct immune cells expression patterns in the discovery cohort. Compared to C1, a higher resting and activated CD4+ T memory cells, memory B cells and Treg cells was confirmed in C0 **(Figure 1A, Figure 1B, Figure 1C, Figure S2B, and Figure S2C)**. In comparison, a lower Th1 and Th2 cells, M1 and M2 macrophages abundance was found in C0. Besides, the expression of immune score and stromal score in C1 was higher than that in C0, in agreement with the higher IPS scores, including Antigen presentation score and Effector cells score **(Figure 1A, Figure 1B, Figure 1C, Figure S2A, Figure S2B, Figure S2C, and Figure S2E)**. Lastly, we identified C1 exhibited higher enrichment score in Cancer-Immunity Cycle than C0, including release of cancer cell antigens, cancer antigen presentation, trafficking of immune cells to tumors, infiltration of immune cells into tumors, and killing of cancer cells (**Figure 1D, Figure 1E**).

**1.2 C0 vs C2**

The WSI cluster C0 and C2 represented distinct immune cells expression patterns in the discovery cohort. Compared to C0, a higher infiltration of Th1 cells, Th2 cells, M1 macrophages, and M2 macrophages was confirmed in C2, the proportion of Macrophage cells and immune cells proliferation was also performed higher in C2 **(Figure 1A, Figure 1B, Figure 1C)**. Cancer-Immunity Cycle also showed significance difference between C0 and C2. In comparison, a higher enrichment score of release of cancer cell antigens, cancer antigen presentation, trafficking of immune cells to tumors, infiltration of immune cells into tumors, and killing of cancer cells was found in C2 (**Figure 1D, Figure 1E**). Besides, the IPS score, including Antigen presentation score and Effector cells score were higher in C2. However, the enrichment score of Checkpoints and Suppressor cells were lower in C2 **(Figure S2A and FigureS2E Figure 1B, Figure 1C, Figure S2B, and Figure S2C)**.

**1.3 C1 vs C2**

The WSI cluster C1 and C2 represented distinct immune cells expression patterns in the discovery cohort. Compared to C2, a higher infiltration of Th1 cells, Th2 cells, and M1 macrophages was confirmed in C1, the proportion of Macrophage cells and Lymphocyte was also performed higher in C1. In comparison, a lower Tregs, Memory B cells, and M2 macrophages abundance was found in C1 **(Figure 1A, Figure 1B, Figure 1C)**. Besides, based on Cancer-Immunity Cycle, a higher enrichment score of release of cancer cell antigens, cancer antigen presentation, infiltration of immune cells into tumors, and killing of cancer cells was found in C1, while trafficking of immune cells to tumors score was higher in C2 (**Figure 1D, Figure 1E**). Lastly, we identified C1 exhibited higher Antigen presentation score and Effector cells score compared with C2 **(Figure S2A and FigureS2E Figure 1B, Figure 1C, Figure S2B, and Figure S2C)**.


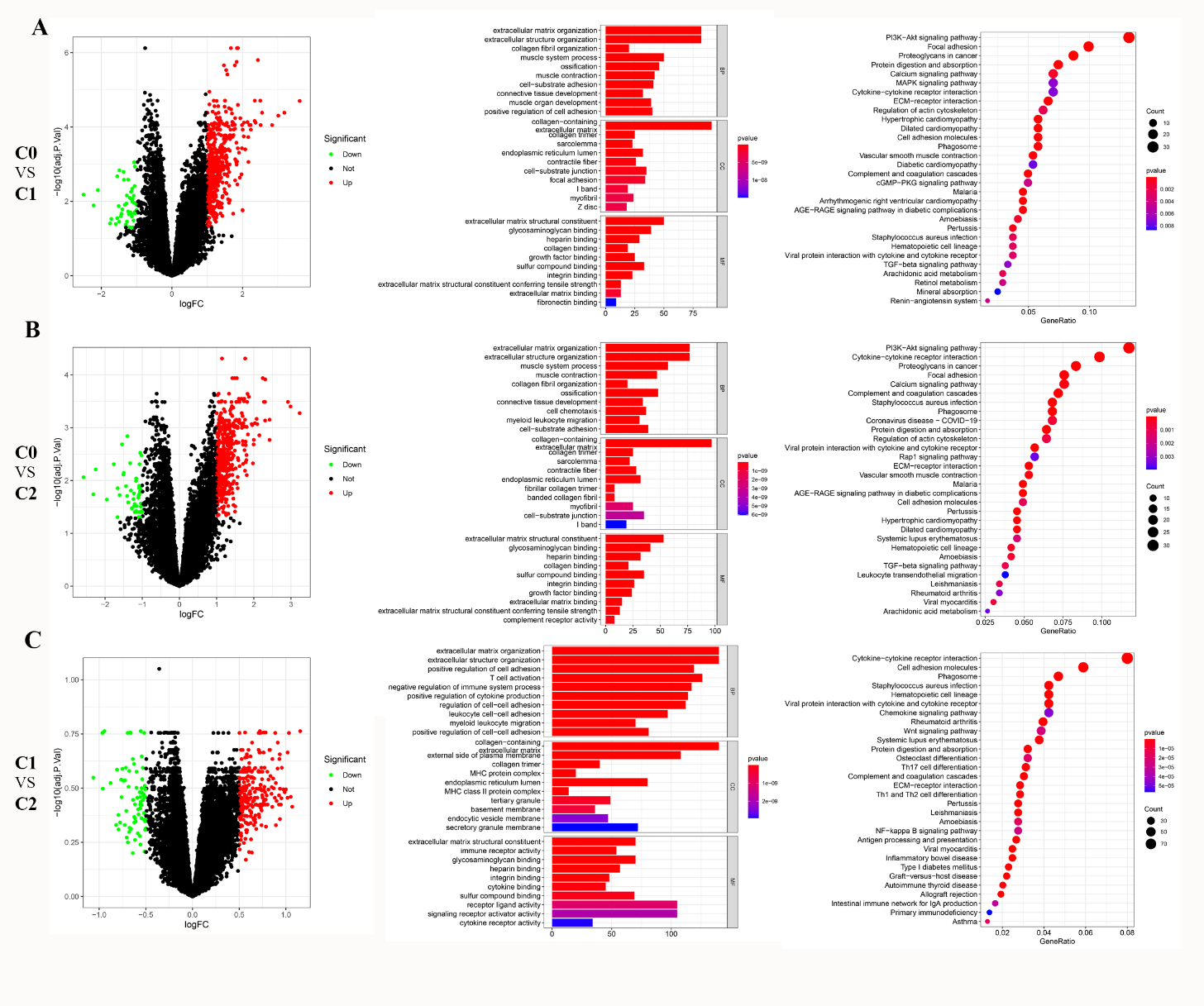
**III. Supplementary Tables and Figures**

**Figure S1. Differential expression analysis and Functional analysis of WSI clusters.** The left parts show differential expression analysis in WSI clusters; the middle parts show GO analysis based on the separate DEGs between WSI clusters; the right parts show KEGG analysis based on the separate DEGs between WSI clusters. (A) 530 DEGs was found in C0 vs C1 (B). 497 DEGs was found in C0 vs C2 (C). 342 DEGs was found in C1 vs C2.


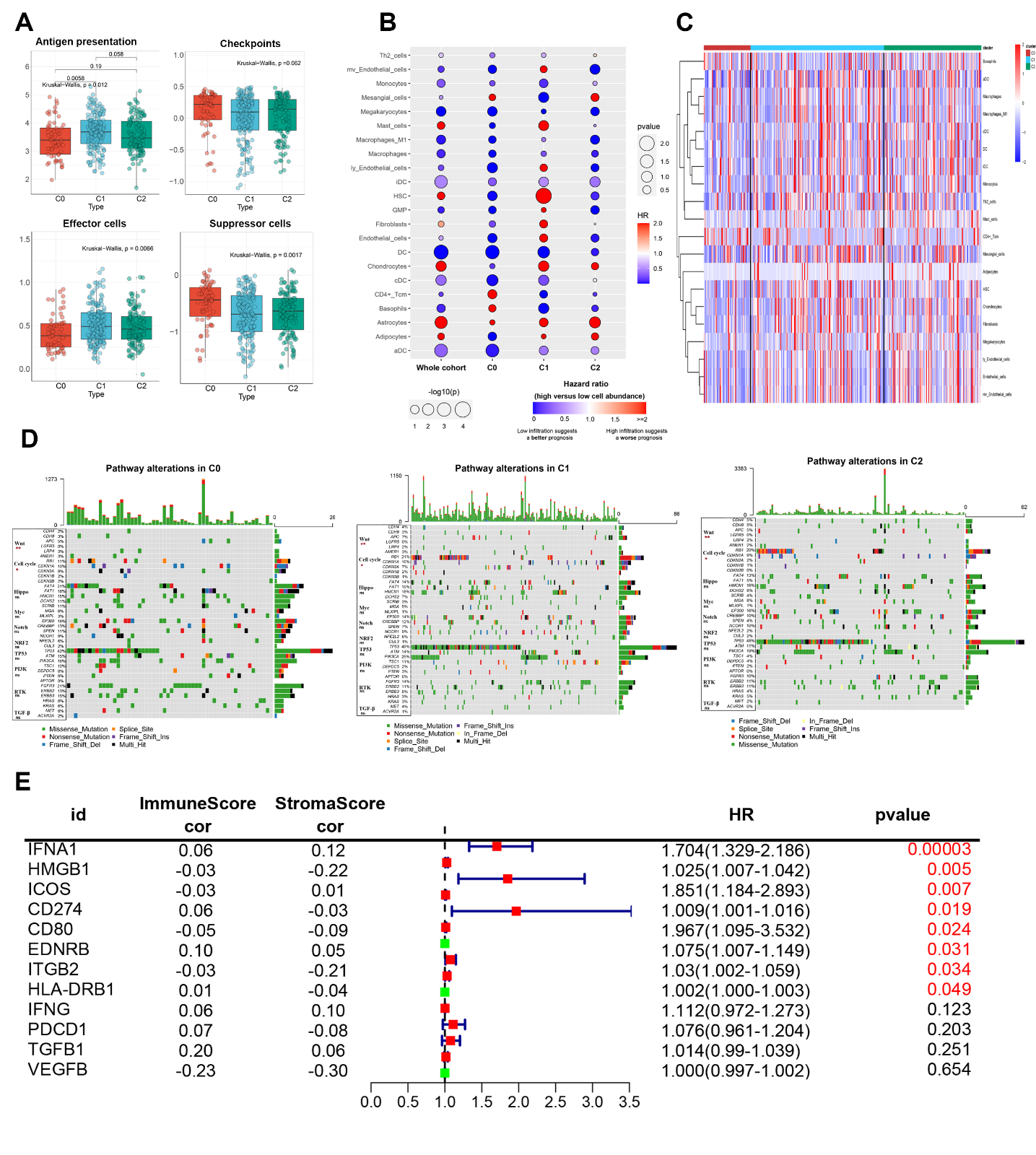
**Figure S2. Immune characterization and Mutation of WSI cluster**

(A) Immunophenoscore (IPS) scores across the WSI clusters. (B-C) Immune cell infiltration in WSI cluster using Xcell algorithm. (D) Mutation profile of WSI cluster in BLCA main dysregulated pathways. (E) COX regression analysis of immune checkpoints in WSI clusters.


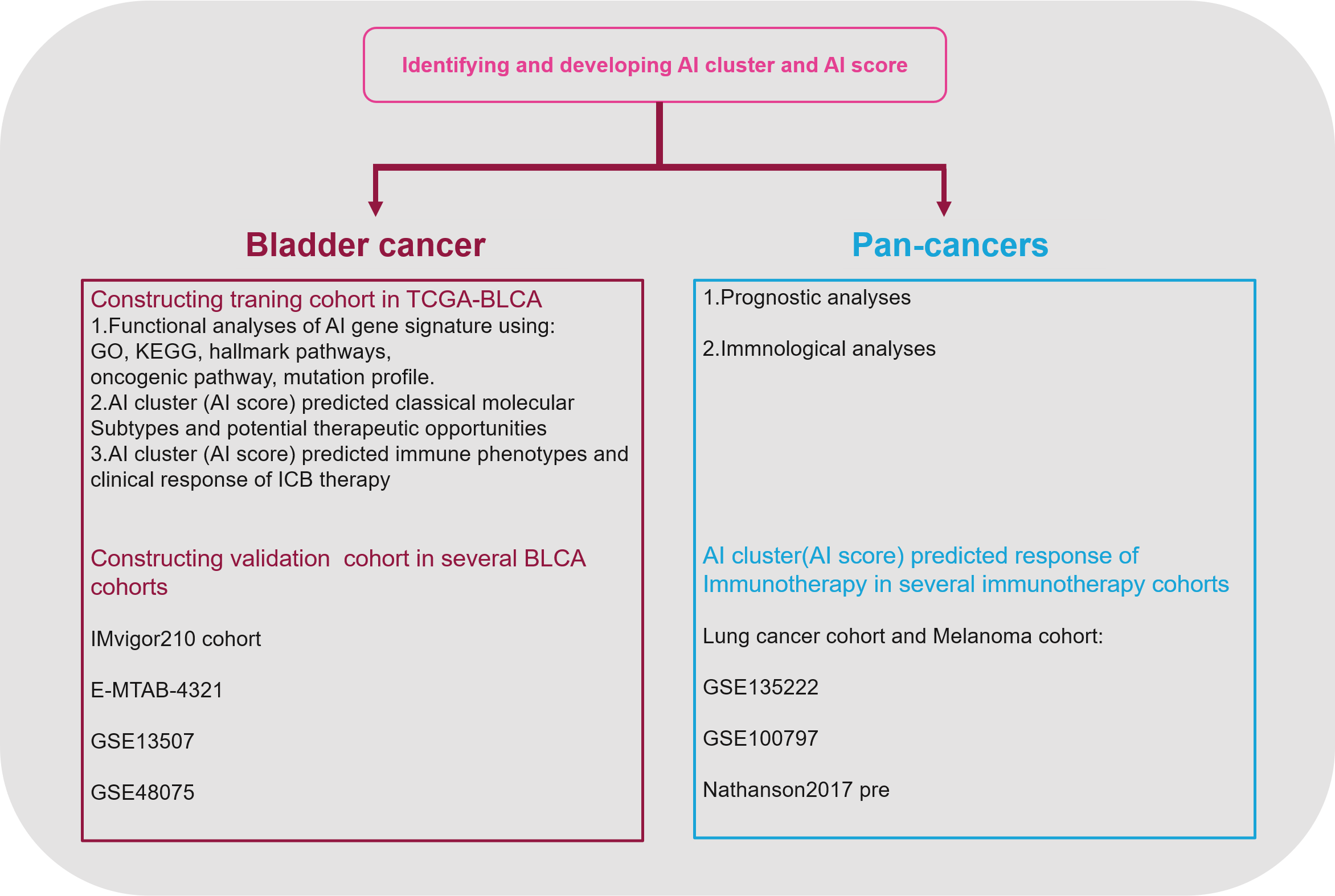


**Figure S3. Construction and validation of AI cluster.** The workflow of constructing AI clusters and the AI score.


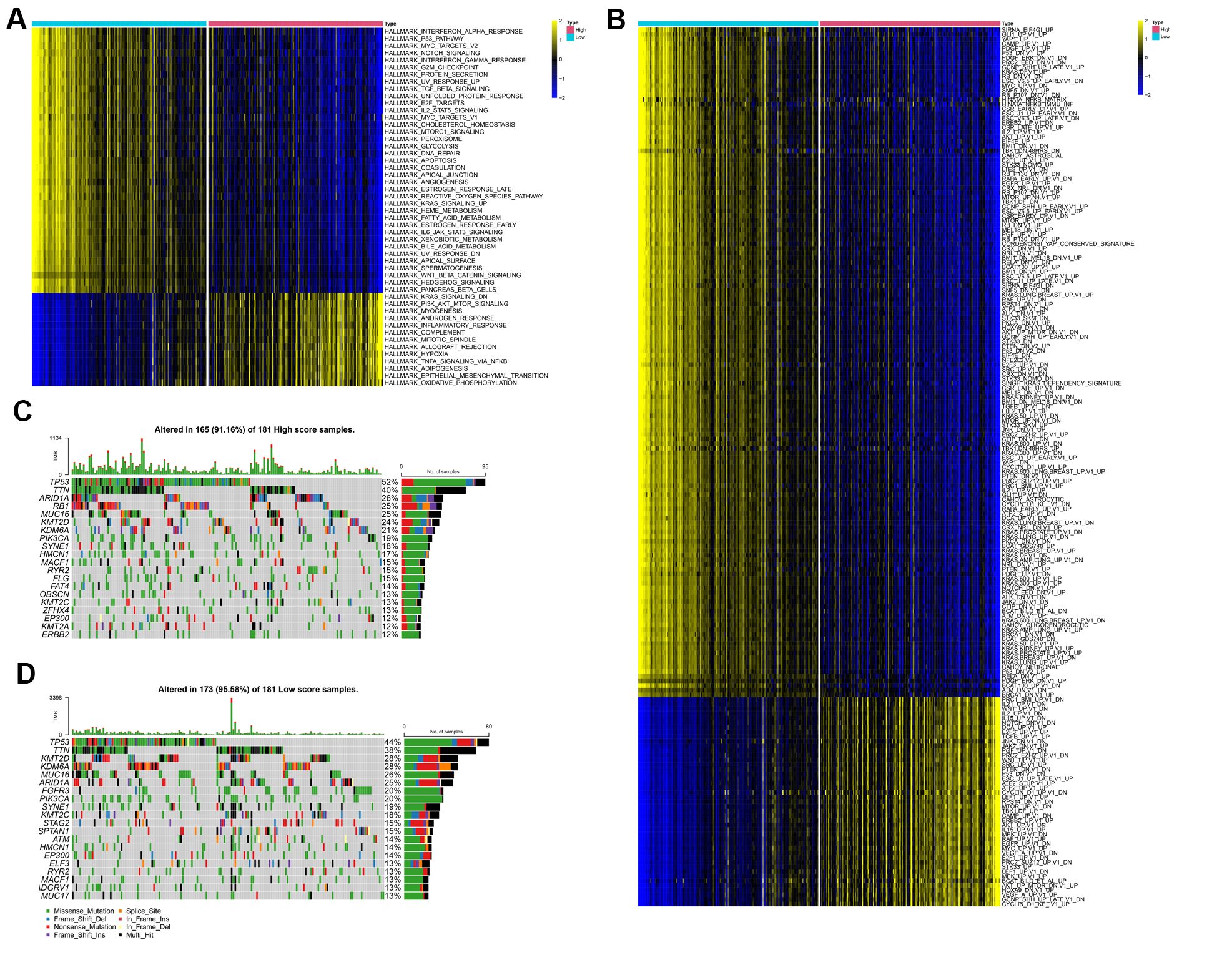


**Figure S4. Functional analyses of AI clusters.** (A) The differences in hallmark pathways between the AI cluster. (B) The differences in oncogenic pathways between the AI clusters. (C-D) The differences in mutational profiles between AI clusters.


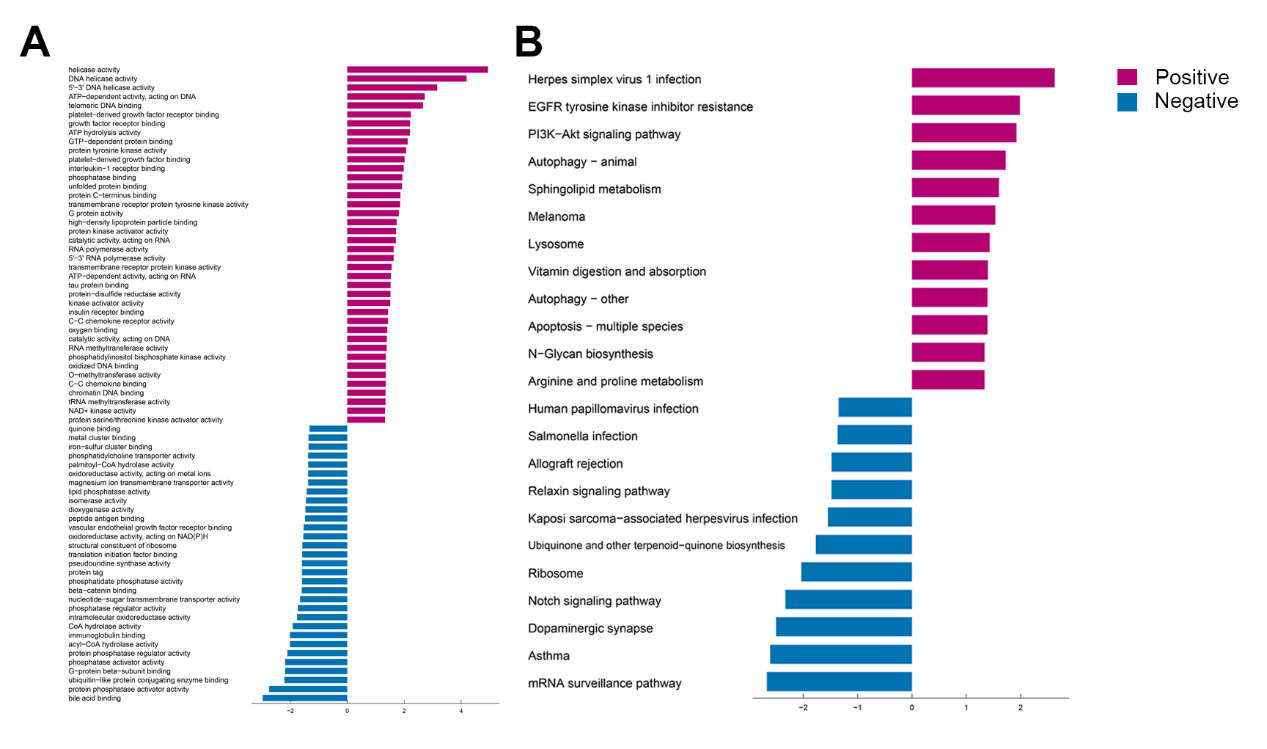


**Figure S5. The differences in GO and KEGG pathways between AI clusters.** (A) GO enrichment of the DEGs between AI clusters shows the activation status of biological pathways in different AI cluster. (B) performs KEGG pathway enrichment based on the DEGs between AI clusters. The blue bar shows the enrichment analysis results based on down-regulated genes in high AI score subtype, representing the negative correlated biological processes and KEGG pathways with the high AI scores, while the red bar shows the enrichment analysis results based on up-regulated genes in high AI score subtype, representing the positive correlated biological processes and KEGG pathways with the high AI scores.


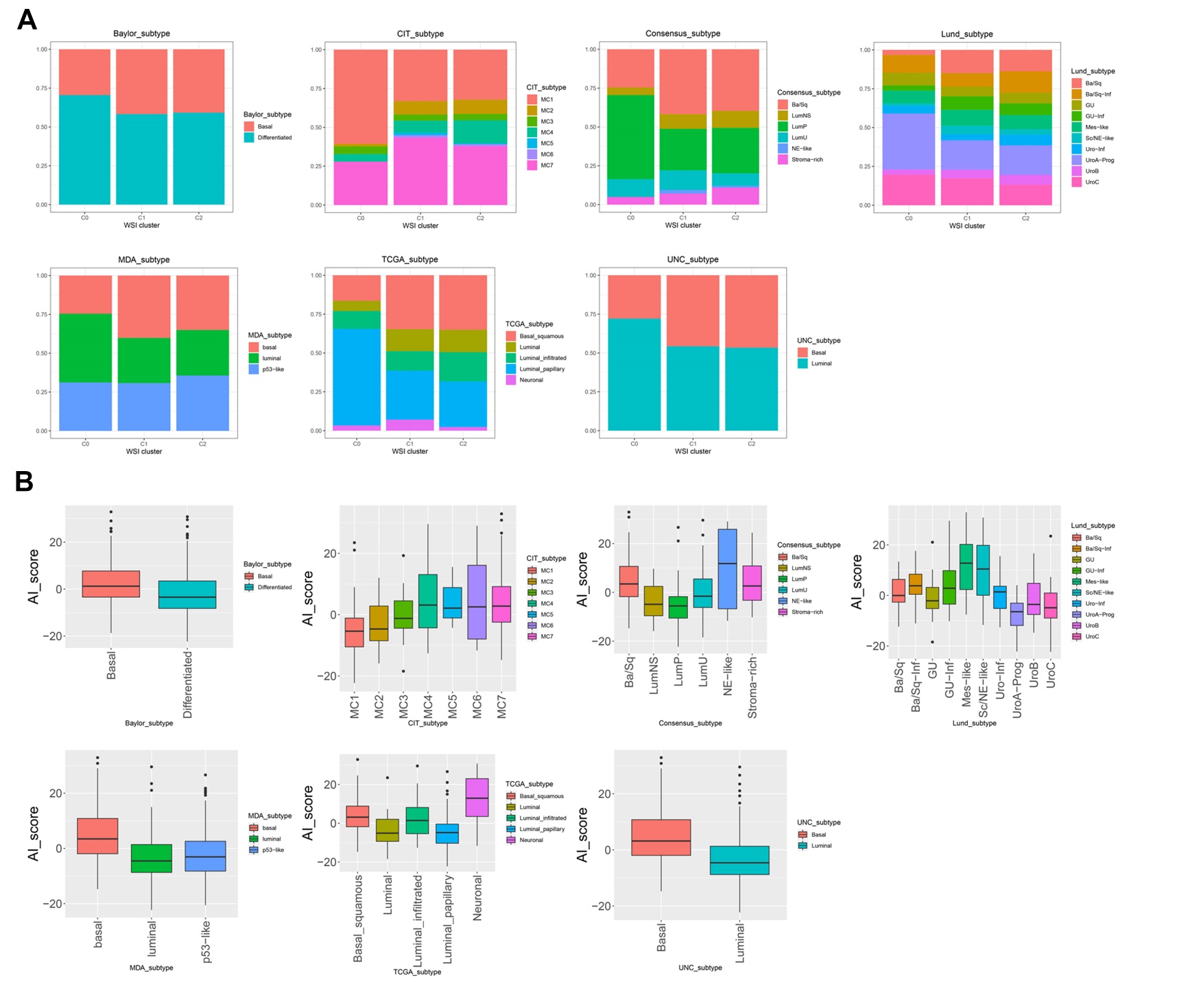
**Figure S6.** (A) The proportions of every subgroup in seven classic subtype systems. (B) The distribution of AI score among different subgroups in all molecular subtype systems.


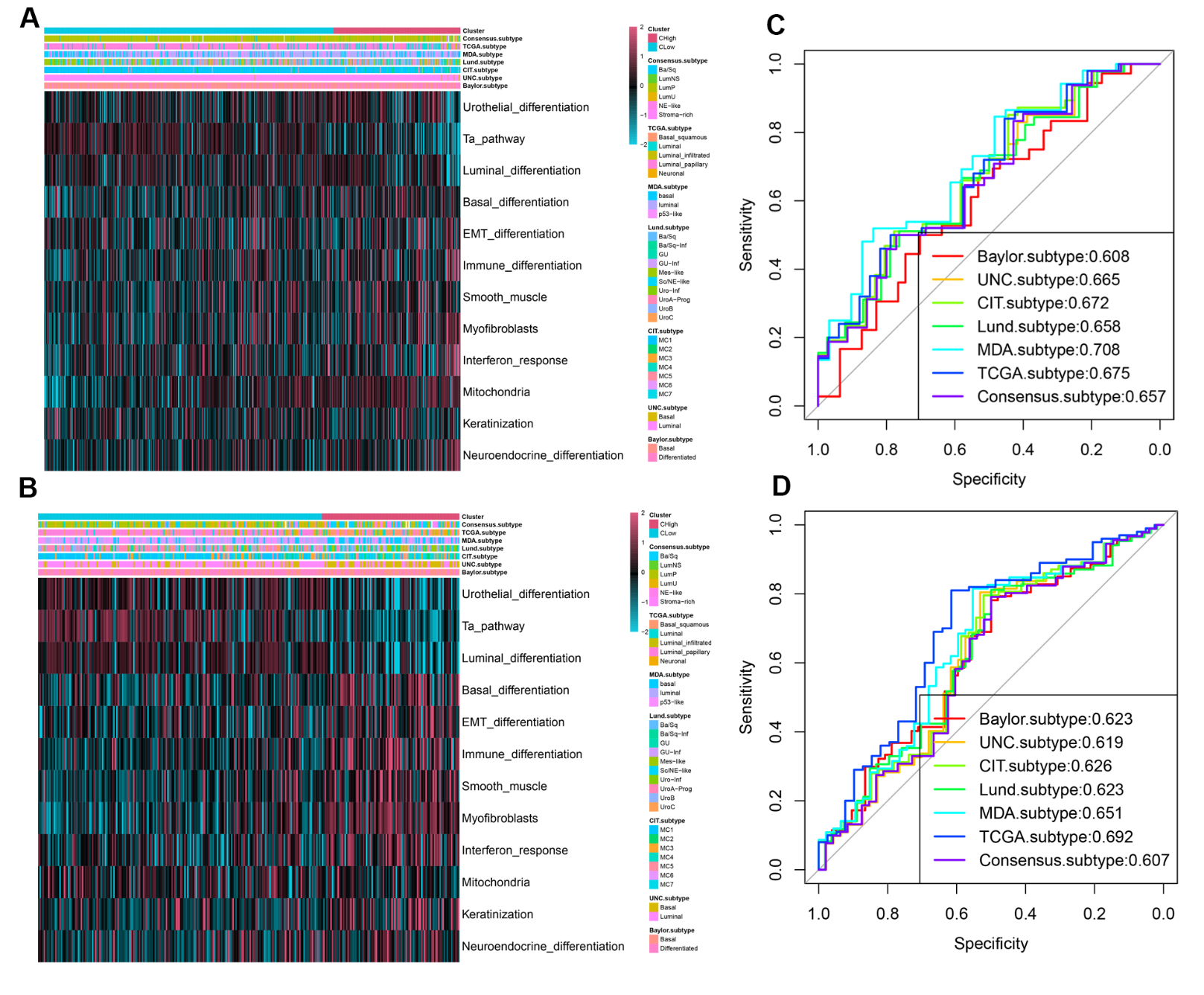
**Figure S7. AI score predicted classical molecular subtypes in two external validation BLCA cohorts**. (A) The correlations between AI score and seven classical molecular subtype classifications in the GEO BLCA cohorts (GSE48075 and GSE32894). (B) ROC curves showed the accuracy of the AI score in predicting seven classical molecular subtypes in the GEO BLCA cohorts. (C) The correlations between AI score and seven classical molecular subtype classifications in the E-MTAB-4321 cohort. (D) ROC curves showed the accuracy of AI score in predicting seven classical molecular subtypes in the E-MTAB-4321 cohort.


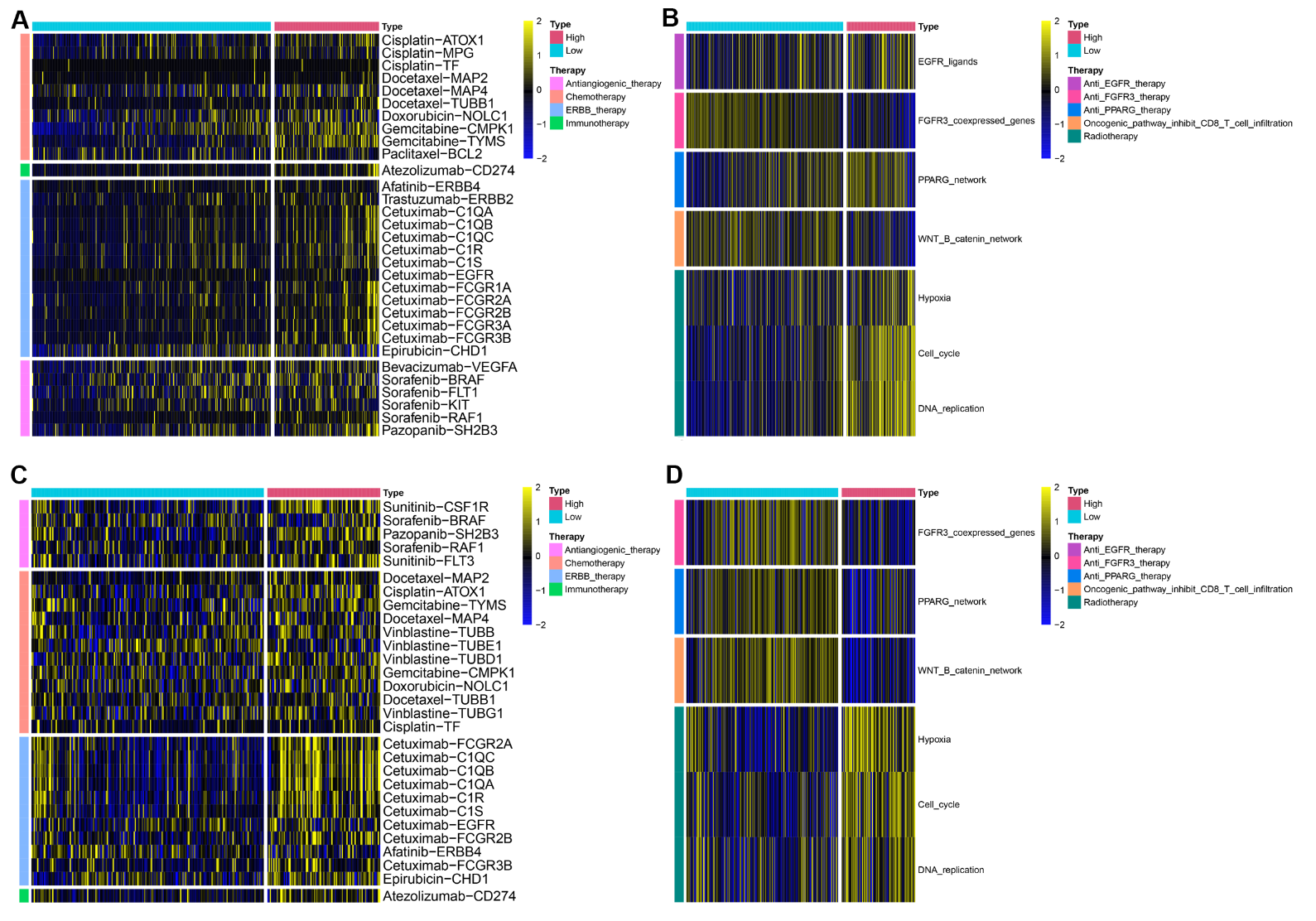
**Figure S8. AI score predicted therapeutic opportunities in two external validation BLCA cohorts**. (A, C) The correlations between AI score and the BLCA-related drug-target genes were screened from the DrugBank database, the upper part shows the correlation between AI score and the E-MTAB-4321 cohort; the lower part shows the correlation between AI score and the GEO BLCA cohorts (GSE48075 and GSE32894). (B, D) The correlations between AI score and the enrichment scores of several therapeutic signatures, such as targeted therapy and radiotherapy, in the E-MTAB-4321 cohort.


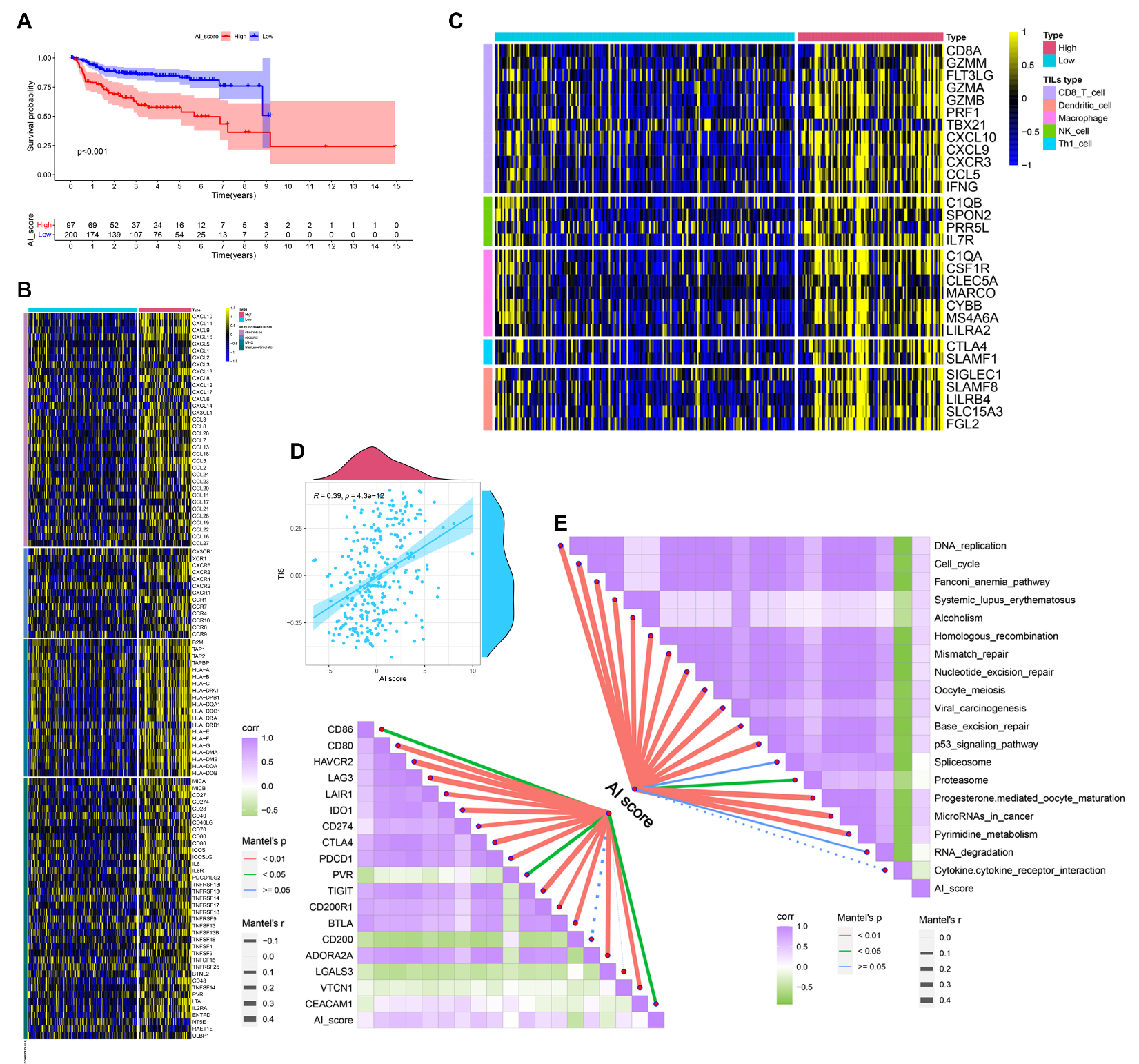
**Figure S9.** **AI score correlated with immune phenotypes and ICB response in the GEO BLCA cohorts** **(GSE48075, GSE32894).** (A) Survival analysis of AI score clusters. (B) The differences in 122 immunomodulators between AI score clusters. (C) The differences in the expression of effector genes of five anticancer TIICs (including CD8+ T cells, NK cells, macrophages, Th1 cells, and dendritic cells) between the AI score clusters. (D) The correlations between AI score and TIS. (E) The lower left part shows the correlations between AI score and the expression of 22 immune checkpoints; the upper right part shows the correlations between AI score and the enrichment scores of positive ICB response-related signatures.


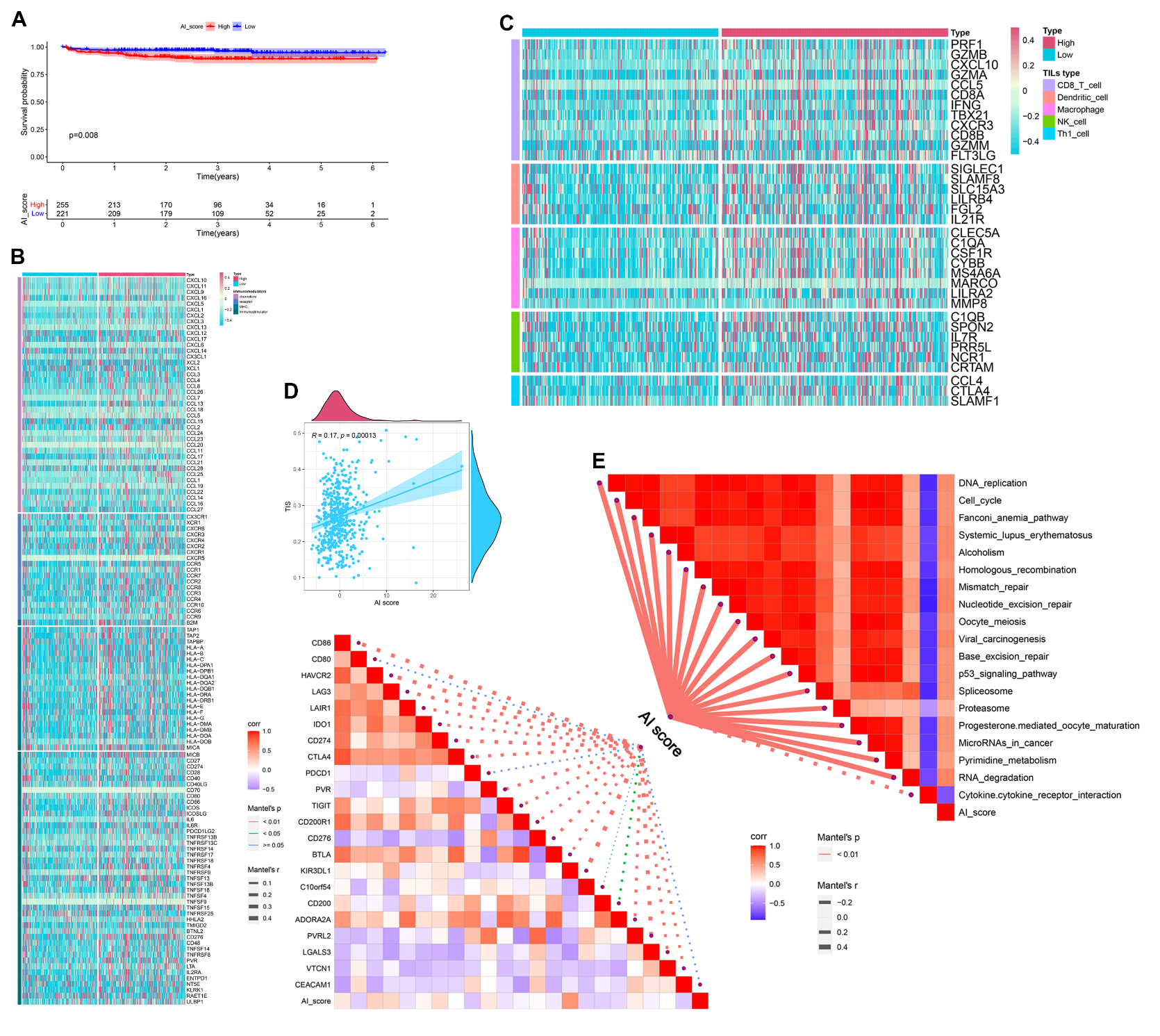
**Figure S10.** **AI score correlated with immune phenotypes and ICB response in the E-MTAB-4321 cohort.** (A) Survival analysis of AI score clusters. (B) The differences in 122 immunomodulators between AI score clusters. (C) The differences in the expression of effector genes of five anticancer TIICs (including CD8+ T cells, NK cells, macrophages, Th1 cells, and dendritic cells) between AI score clusters. (D) The correlations between AI score and TIS. (E) The lower left part shows the correlations between AI score and the expression of 22 immune checkpoints; the upper right part shows the correlations between AI score and the enrichment scores of positive ICB response-related signatures.


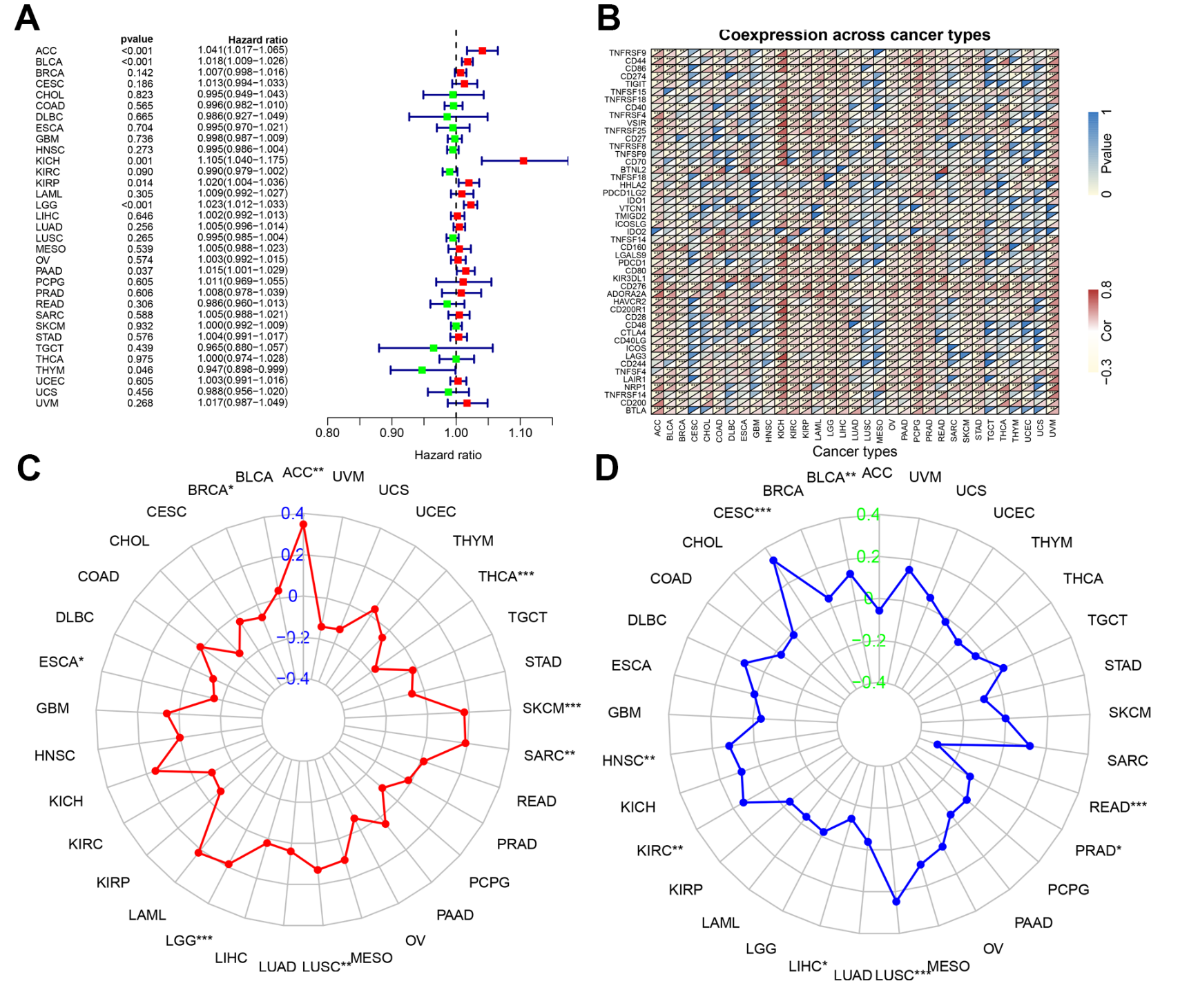
**Figure S11. Pancancer analyses of the AI score and AI gene signature.** (A) The prognostic analyses of AI score across cancers using a univariate Cox regression model. A hazard ratio >1 indicated a risk factor, and a hazard ratio <1 represented a protective factor. (B) Correlations between AI score and potential immune checkpoints. (C) The correlations between AI score and TMB in all cancers. (G) The correlations between AI score and MSI across cancers. The asterisks indicate a significant statistical p-value calculated with Spearman correlation analysis. (*P < 0.05; **P < 0.01; ***P < 0.001).


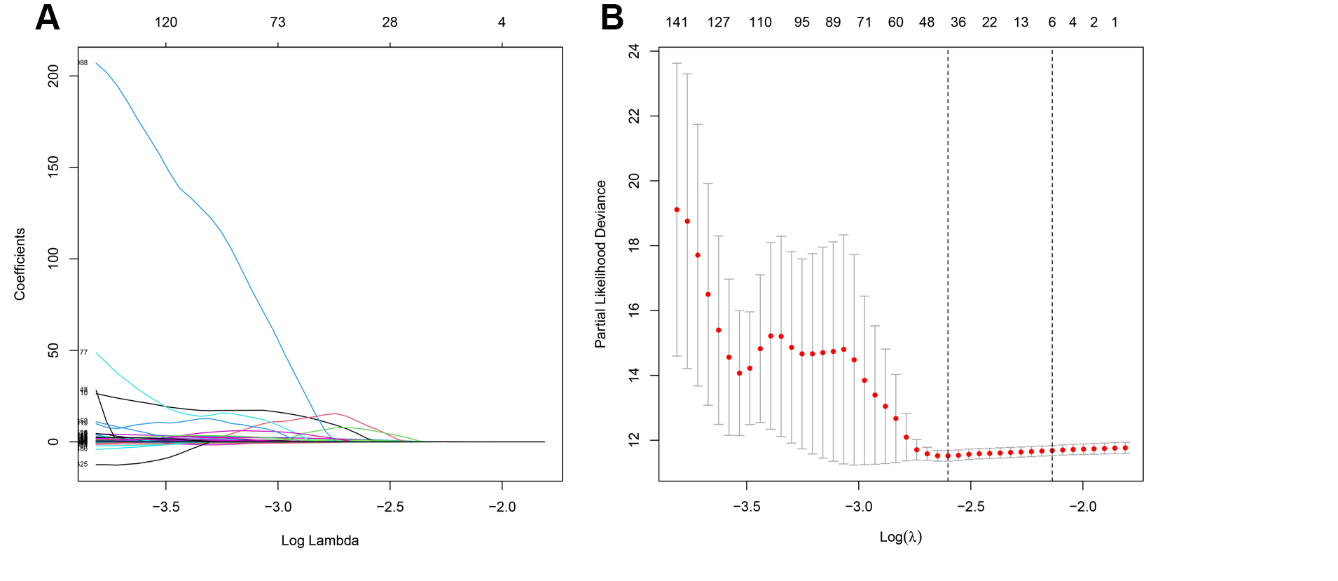


**Figure S12. Identification of AI score candidate member.** (A) LASSO coefficient profiles of 802 prognostic DEGs between WSI clusters. The coefficient profile plot was developed against the log (Lambda) sequence. (B) Cross-validation for turning parameter selection via minimum criteria in the LASSO regression model. Two dotted vertical lines were plotted at the optimal values using the minimum criteria. Optimal RNAs with the best discriminative capability (28 in number) were selected for developing the AI score.


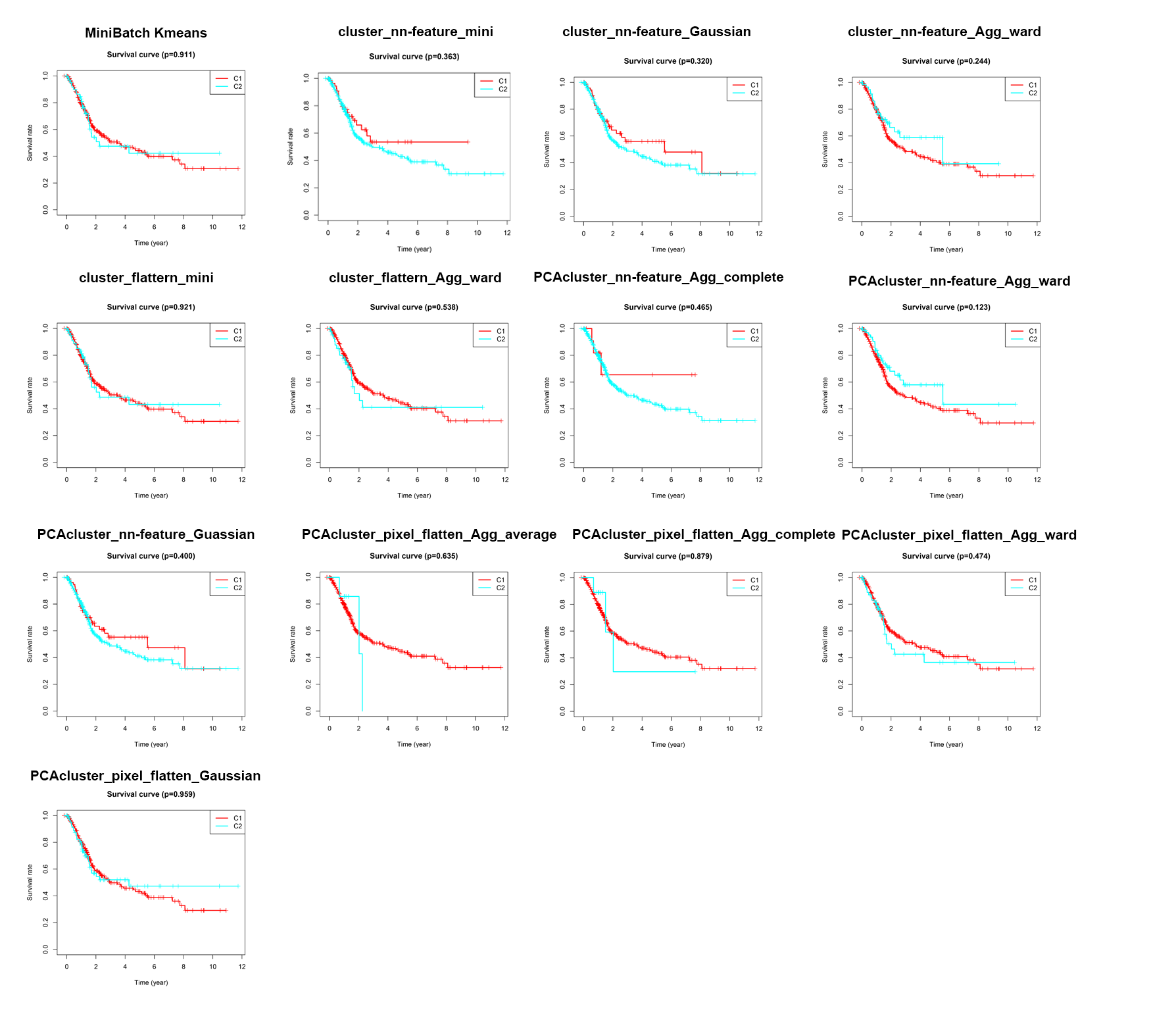


**Figure S13. Survival analysis of Binary Classification Model identified by deep learning procedure.** Mini batch K-Means, hierarchical clustering and Gaussian Mixture Mode (GMM) were respectively conducted for clustering. (*P < 0.05; **P < 0.01; ***P < 0.001).


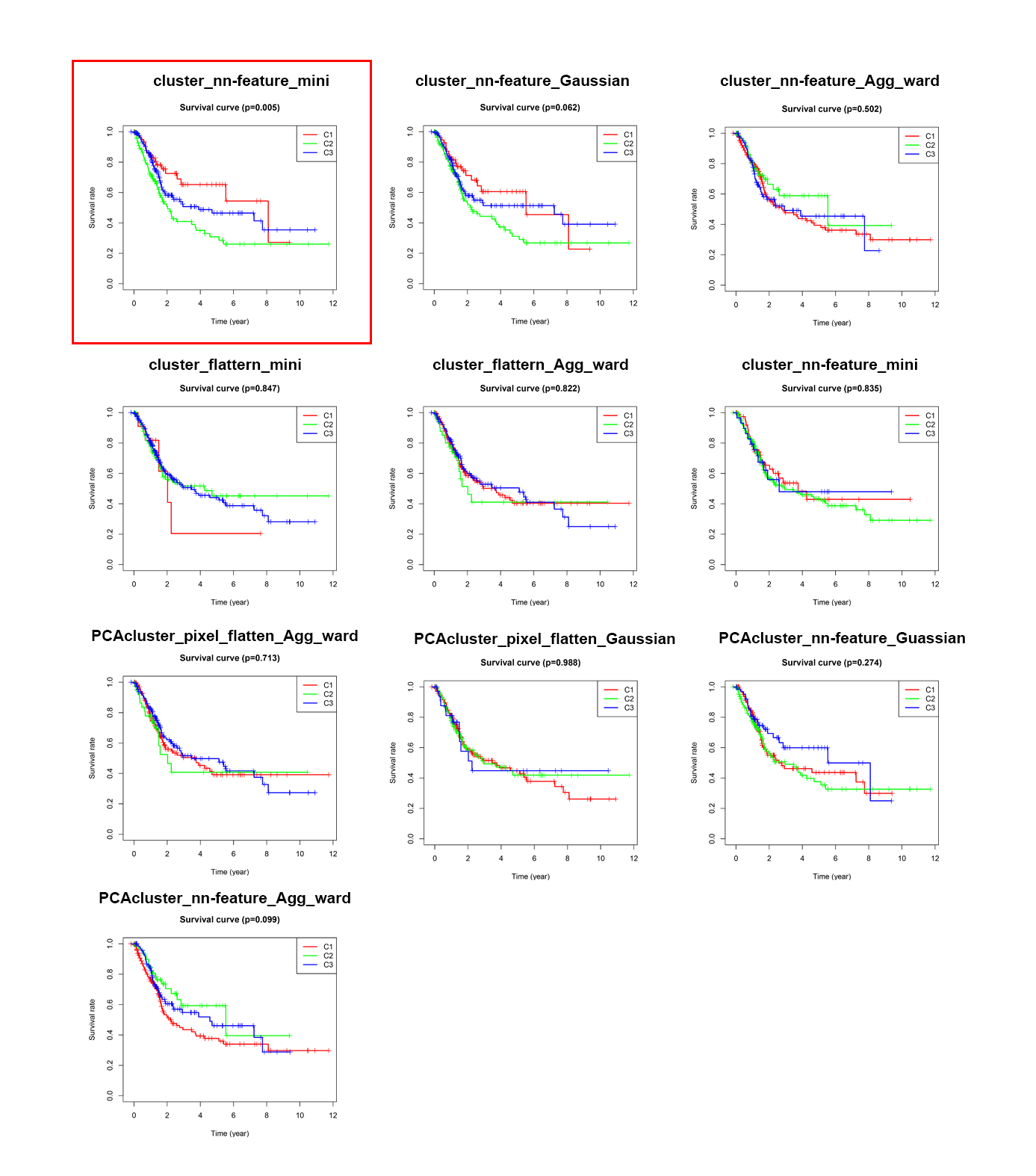
 **Figure S14. Survival analysis of Triple-classification Model identified by deep learning procedure.** Mini batch K-Means, hierarchical clustering and Gaussian Mixture Mode (GMM) were respectively conducted for clustering. The model marked by red rectangle was selected to develop WSI cluster. (*P < 0.05; **P < 0.01; ***P < 0.001).


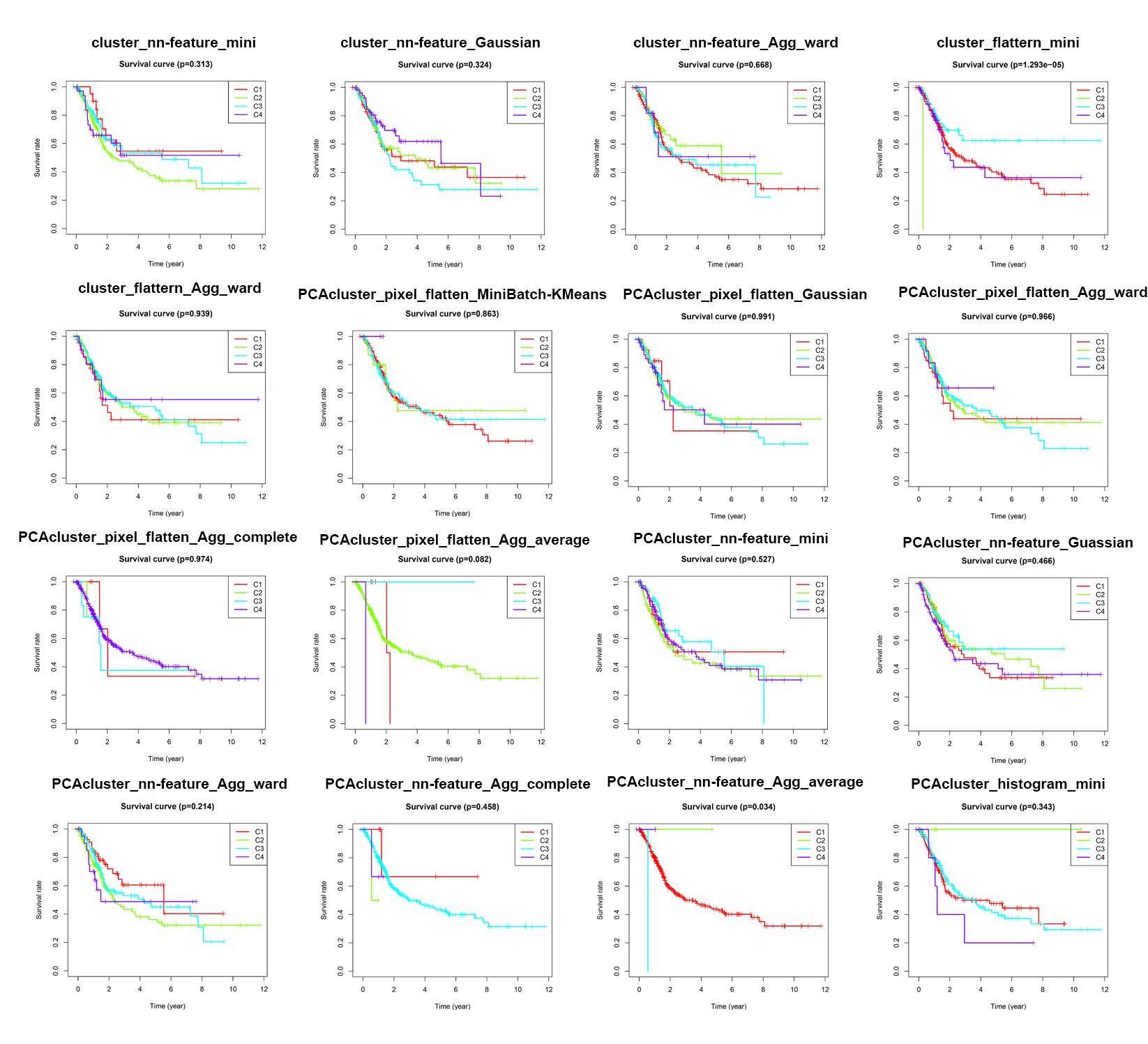
**Figure S15. Survival analysis of Four-classification Model identified by deep learning procedure.**

Mini batch K-Means, hierarchical clustering and Gaussian Mixture Mode (GMM) were respectively conducted for clustering. (*P < 0.05; **P < 0.01; ***P < 0.001).


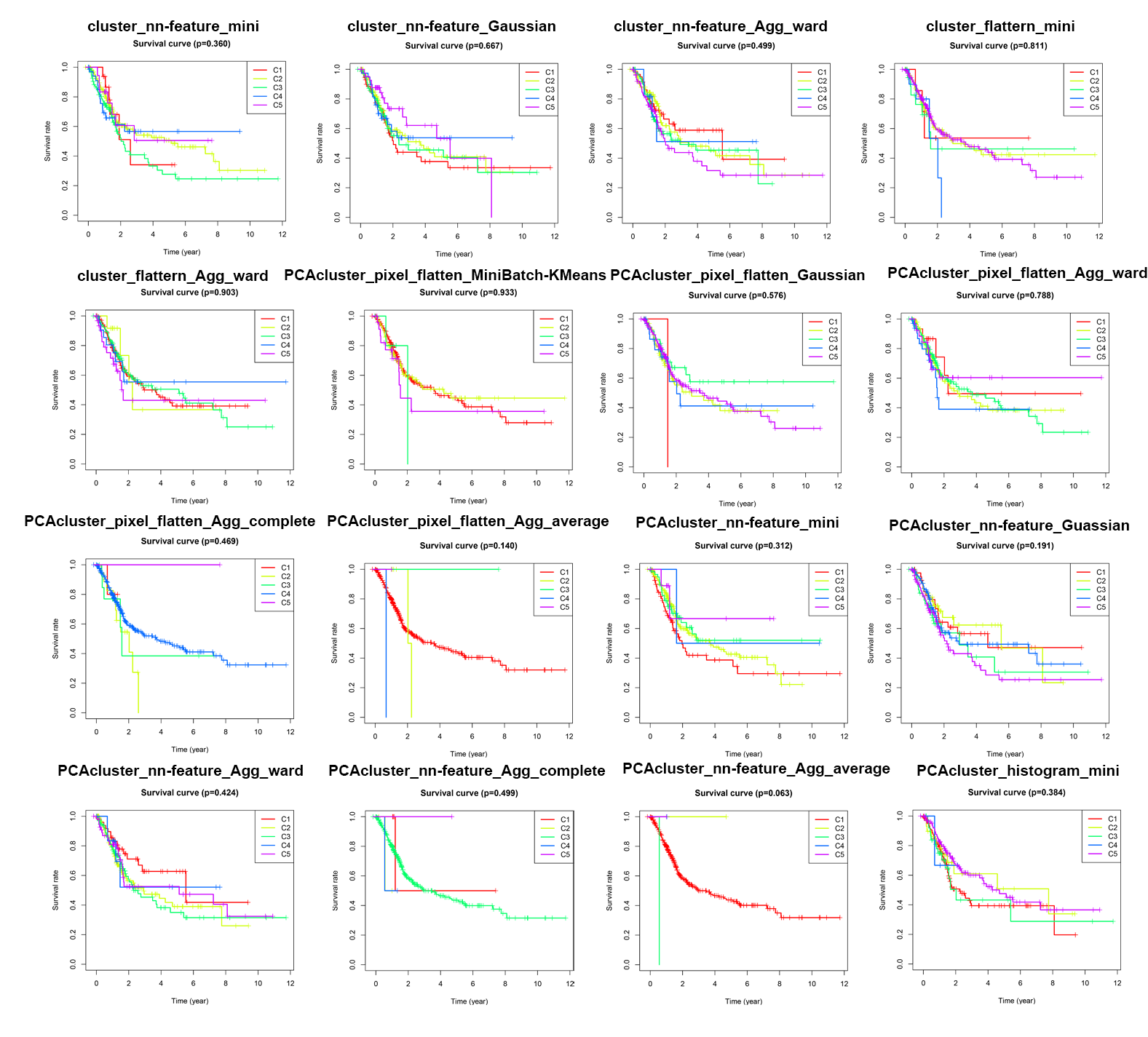


**Figure S16. Survival analysis of Five-classification Model identified by deep learning procedure.**

Mini batch K-Means, hierarchical clustering and Gaussian Mixture Mode (GMM) were respectively conducted for clustering. (*P < 0.05; **P < 0.01; ***P < 0.001).


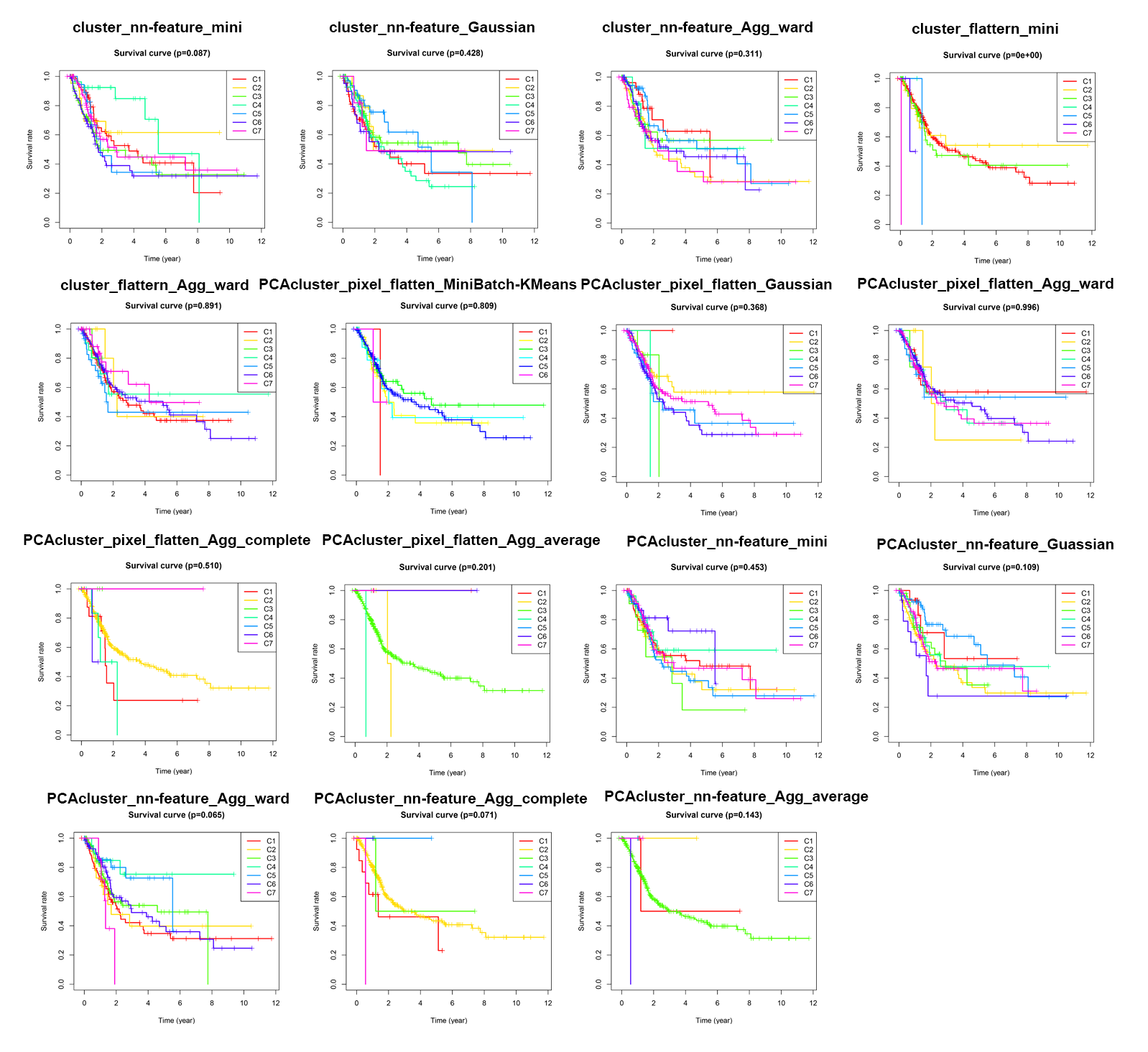
**Figure S17. Survival analysis of Seven-classification Model identified by deep learning procedure.**

Mini batch K-Means, hierarchical clustering and Gaussian Mixture Mode (GMM) were respectively conducted for clustering. (*P < 0.05; **P < 0.01; ***P < 0.001).

| Table.S1. Univariable and multivariable analyses for overall survival in patients with BLCA | | | | |
| --- | --- | --- | --- | --- |
| **Variables** | **Univariable analysis  (N=333)** | | **Multivariable analysis  (N=333)** | |
|  | **OR (95%CI)** | **P** | **OR (95%CI)** | **P** |
| **mRNA cluster**(luminal vs basal) | 1.470(1.041-2.076) | 0.029 | 1.562(1.069-2.282) | 0.021 |
| **Age** (years) | 1.043(1.024-1.063) | 0.000 | 1.039(1.019-1.059) | 0.000 |
| **Gender** (female vs. male) | 0.929(0.630-1.370) | 0.711 | 0.845(0.562-1.270) | 0.417 |
| **Stage** | 1.975(1.555-2.507) | 0.000 | 1.328(0.848-2.082) | 0.215 |
| **T** | 1.760(1.367-2.267) | 0.000 | 1.425(1.044-1.945) | 0.026 |
| **M** | 3.064(1.246-7.536) | 0.015 | 1.383(0.544-3.515) | 0.496 |
| **N** | 1.590(1.327-1.905) | 0.000 | 1.176(0.845-1.636) | 0.336 |
| **ImmuneScore** | 0.424(0.137-1.318) | 0.138 | 0.115(0.028-0.466) | 0.002 |
| **StromaScore** | 25.945(3.55-189.610) | 0.001 | 16.243(1.984-132.975) | 0.009 |
| **Cluster** |  |  |  |  |
| C0 | 0.441(0.231-0.841) | 0.231 | 0.419(0.212-0.829) | 0.012 |
| C1 | 0.848(0.601-1.197) | 0.601 | 0.624(0.43-0.907) | 0.013 |
| C2 | 1.680(1.189-2.373) | 1.189 | NA | NA |

| Table.S2. Clinicopathological characteristics of patients with BLCA in TCGA | | | | | | |
| --- | --- | --- | --- | --- | --- | --- |
| **Variables** | **TCGA** | | | | |  |
|  | **C0(n=43)** | | **C1(n=169)** | | **C2(n=121)** | |
|  | **N** | **%** | **N** | **%** | **N** | **%** |
| **mRNA cluster** |  |  |  |  |  |  |
| Luminal | 32 | 74.40% | 86 | 50.90% | 65 | 53.70% |
| Basal | 11 | 25.60% | 83 | 49.10% | 56 | 46.30% |
| **Age (median,IQR,Y)** | (67,59.5-73) | | (68,60-76) | | (71,63-78) | |
| **Gender** |  |  |  |  |  |  |
| Male | 32 | 74.40% | 133 | 78.70% | 85 | 70.20% |
| Female | 11 | 25.60% | 36 | 21.30% | 36 | 29.80% |
| **Stage** |  |  |  |  |  |  |
| Stage I | 1 | 2.30% | 0 | 0.00% | 1 | 0.80% |
| Stage II | 20 | 46.50% | 45 | 26.60% | 30 | 24.80% |
| Stage III | 11 | 25.60% | 65 | 38.50% | 44 | 36.40% |
| Stage IV | 11 | 25.60% | 59 | 34.90% | 46 | 38.00% |
| **T** |  |  |  |  |  |  |
| T0 | 1 | 2.30% | 0 | 0.00% | 0 | 0.00% |
| T1 | 2 | 4.70% | 0 | 0.00% | 1 | 0.80% |
| T2 | 19 | 44.20% | 51 | 30.20% | 37 | 30.60% |
| T3 | 18 | 41.90% | 93 | 55.00% | 61 | 50.40% |
| T4 | 3 | 7.00% | 25 | 14.80% | 22 | 18.20% |
| **M** |  |  |  |  |  |  |
| M0 | 42 | 97.70% | 166 | 98.20% | 118 | 97.50% |
| M1 | 1 | 2.30% | 3 | 1.80% | 3 | 2.50% |
| **N** |  |  |  |  |  |  |
| N0 | 33 | 76.70% | 111 | 65.70% | 76 | 62.80% |
| N1 | 5 | 11.60% | 16 | 9.50% | 18 | 14.90% |
| N2 | 5 | 11.60% | 41 | 24.30% | 23 | 19.00% |
| N3 | 0 | 0.00% | 1 | 0.60% | 4 | 3.30% |
| **ImmuneScore(median,IQR)** | (521.274, -163.861-1176.498) | | (804.668, 199.773-1361.855) | | (643.295, 193.972-1243.181) | |
| **StromaScore (median,IQR)** | (-814.376, -1349.310--89.730) | | (-151.591, -900.602-389.724) | | (-250.455, -798.130-248.450) | |

| Table.S3. Performance of patch classification models | | | | |
| --- | --- | --- | --- | --- |
| Model | Accuary | Auc | Specificity | Sensitivity |
| Model-1 | 92.09% | 0.98 | 0.962 | 0.9197 |
| Model-2 | 93.44% | 0.98 | 0.9394 | 0.9305 |
| Model-3 | 90.92% | 0.98 | 0.9152 | 0.9 |
| Model-4 | 94.89% | 0.99 | 0.929 | 0.9636 |
| *model-1 classifies WSI blocks into pure stromal, necrosis and tumor-containing blocks,   model-2 classifies tumor-containing blocks into high-immune and low-immune in tumor-containing blocks,   model-3 and model-4 classify high-immune and low-immune blocks into high-stromal and low-stromal blocks respectively | | | | |

| Table.S4. Ablation experiments of 3-year prediction features | | | | | |
| --- | --- | --- | --- | --- | --- |
| Feature | BPNN | Accuary | Auc | Specificity | Sensitivity |
| Feature-1 | 21-32-16-1 | 0.8067 | 0.87 | 0.8526 | 0.7272 |
| Feature-2 | 18-32-16-1 | 0.7467 | 0.83 | 0.8588 | 0.6 |
| Feature-3 | 6-12-1 | 0.6133 | 0.63 | 0.7 | 0.44 |
| Feature-1+2 | 39-32-16-1 | 0.8133 | 0.92 | 0.8421 | 0.7636 |
| Feature-1+3 | 27-32-16-1 | 0.8 | 0.86 | 0.8333 | 0.75 |
| Feature-2+3 | 24-32-16-1 | 0.7733 | 0.84 | 0.8824 | 0.6408 |
| Feature-1+2+3 | 45-64-32-1 | 0.86 | 0.95 | 0.8353 | 0.8923 |
| *Feature 1 shows image feature  Feature 2 shows tumor microenvironment feature  Feature 3 shows clinical feature | | | | | |
