# Supplementary material for: Artificial Intelligence Meets Whole Slide Images: Deep Learning Model Shapes an Immune-Hot Tumor and Guides Precision Therapy in Bladder Cancer
Source: J Oncol. 2022 Sep 19;2022:8213321. doi: 10.1155/2022/8213321 (PMC9553530; doi:10.1155/2022/8213321)
Supplement: Supplementary Materials — Figure S1: differential expression analysis and functional analysis of WSI clusters. The left parts show differential expression analysis in WSI clusters; the middle parts show GO analysis based on the separate DEGs between WSI clusters; and the right parts show KEGG analysis based on the separate DEGs between WSI clusters. (A) 530 DEGs was found in C0 vs. C1. (B) 497 DEGs was found in C0 vs. C2. (C) 342 DEGs was found in C1 vs. C2. Figure S2: immune characterization and mutation of WSI cluster. (A) Immunophenoscore (IPS) across the WSI clusters. (B, C) Immune cell infiltration in WSI cluster using Xcell algorithm. (D) Mutation profile of WSI cluster in BLCA main dysregulated pathways. (E) Cox regression analysis of immune checkpoints in WSI clusters. Figure S3: construction and validation of AI cluster. The workflow of constructing AI clusters and the AI score. Figure S4: functional analyses of AI clusters. (A) The differences in hallmark pathways between the AI cluster. (B) The differences in oncogenic pathways between the AI clusters. (C, D) The differences in mutational profiles between AI clusters. Figure S5: the differences in GO and KEGG pathways between AI clusters. (A) GO enrichment of the DEGs between AI clusters shows the activation status of biological pathways in different AI cluster. (B) KEGG pathway enrichment based on the DEGs between AI clusters. The blue bar shows the enrichment analysis results based on down-regulated genes in high AI score subtype, representing the negative correlated biological processes and KEGG pathways with the high AI scores, while the red bar shows the enrichment analysis results based on upregulated genes in high AI score subtype, representing the positive correlated biological processes and KEGG pathways with the high AI scores. Figure S6: (A) the proportions of every subgroup in seven classic subtype systems. (B) The distribution of AI score among different subgroups in all molecular subtype systems. Figure S7: AI score [file 8213321.f1.zip › Supplementary Data.pdf]

Basic information of TCGA-BLCA cohort

Patient.ID	Survival M	Status	Age	Gender	BMI	Smoking	Histology	Stage	Grade	pM	pN	pT	LVI	Neoadjuvai	Radiotherapy
TCGA-DK-A1A7	18.66667	Alive	67	female	21.91381	yes	Non-Papill	Stage iv	High Grad	M0	N2	T3	YES	No	NO
TCGA-CF-A3MF	12.76667	Alive	34	male	NA	yes	Papillary	Stage iii	Low Grade	M0	N0	T3	NA	No	YES
TCGA-CF-A3MI	12.33333	Alive	62	male	24.21875	yes	Papillary	Stage ii	Low Grade	M0	N0	T2	NA	No	NO
TCGA-CF-A47Y	12.43333	Alive	55	male	23.52941	yes	Papillary	Stage ii	Low Grade	M0	N0	T2	NA	No	NO
TCGA-FJ-A3Z7	31.5	Alive	76	male	27.42857	yes	Non-Papill	Stage iv	High Grad	MX	N2	T4a	YES	Yes	NO
TCGA-E7-A8O8	0.433333	Alive	59	male	19.47341	yes	Non-Papill	Stage ii	High Grad	M0	N0	T2	YES	No	NA
TCGA-DK-A3X1	66.96667	Alive	78	female	NA	yes	Non-Papill	Stage iii	High Grad	M0	N0	T3	NO	No	NO
TCGA-XF-A8HG	15.56667	Dead	69	male	28.69593	yes	Non-Papill	Stage iii	High Grad	MX	N0	T3b	YES	No	NA
TCGA-CF-A47X	12.8	Alive	60	male	20.2449	yes	Papillary	Stage ii	Low Grade	M0	N0	T2	NA	No	YES
TCGA-DK-A3IK	4.866667	Dead	87	male	26.64719	yes	Papillary	Stage iv	High Grad	M0	N2	T3	YES	No	NA
TCGA-CF-A47S	11.1	Alive	41	male	21.29529	yes	Papillary	Stage ii	Low Grade	M0	N0	T2	NA	No	YES
TCGA-UY-A78N	88.03333	Dead	80	male	25.05931	yes	Non-Papill	Stage iv	High Grad	MX	N1	T2	NO	No	NO
TCGA-DK-A1AE	16.36667	Alive	84	male	20.88276	yes	Non-Papill	Stage iii	High Grad	M0	N0	T3	YES	No	NO
TCGA-UY-A9PF	3.9	Alive	77	male	31.84806	yes	Papillary	Stage iv	High Grad	MX	N2	T3a	NA	No	NO
TCGA-ZF-A9R0	22.66667	Dead	82	male	27.04164	yes	Non-Papill	Stage iii	High Grad	M0	NX	T3b	NA	No	NO
TCGA-CF-A9FF	12.03333	Alive	52	male	19.36247	yes	Papillary	Stage ii	High Grad	M0	N0	T2	YES	No	NO
TCGA-H4-A2HO	1.533333	Alive	53	male	22.02432	yes	Papillary	Stage iii	High Grad	MX	N0	T4a	NO	No	NO
TCGA-2F-A9KQ	96.2	Alive	69	male	25.5102	yes	Non-Papill	Stage iii	High Grad	M0	N0	T3a	YES	No	NO
TCGA-GU-A763	33.23333	Alive	72	male	24.6755	yes	Papillary	Stage iii	High Grad	M0	N0	T4	NO	No	NO
TCGA-CF-A47W	12.26667	Alive	42	male	21.00767	yes	Papillary	Stage ii	Low Grade	M0	N0	T2	NA	No	NO
TCGA-CF-A3MH	13.26667	Alive	75	male	21.67126	yes	Papillary	Stage ii	Low Grade	M0	N0	T2	NA	No	YES
TCGA-CF-A9FL	18.83333	Dead	85	male	16.52893	yes	Papillary	Stage iii	High Grad	M0	N0	T3b	YES	No	NO
TCGA-XF-A8HF	98.46667	Dead	80	male	24.78426	yes	Non-Papill	Stage iii	High Grad	M0	N0	T3a	NO	No	NO
TCGA-DK-A6B6	37.16667	Alive	57	male	34.02035	yes	Papillary	Stage ii	High Grad	MX	NX	NA	NA	No	NO
TCGA-DK-A2I1	18.2	Alive	73	female	32.20576	yes	Papillary	Stage ii	High Grad	M0	N0	T2b	YES	No	NO
TCGA-ZF-A9R9	28.8	Dead	58	male	NA	yes	Non-Papill	Stage iv	High Grad	MX	N2	T3b	YES	No	NA
TCGA-CF-A47V	12.63333	Alive	52	male	20.17715	yes	Papillary	Stage ii	High Grad	M0	N0	T2	NA	No	YES
TCGA-DK-A3IL	13.76667	Dead	79	female	28.51563	yes	Non-Papill	Stage iv	High Grad	M0	N2	T3	NA	No	NA
TCGA-CF-A8HX	11.5	Alive	55	female	23.92242	yes	Papillary	Stage ii	High Grad	M0	N0	T2	NA	No	NO
TCGA-BT-A42E	36.93333	Alive	74	male	28.71972	yes	Non-Papill	Stage iii	High Grad	M0	N0	T3a	YES	No	NO
TCGA-XF-A9SH	65.7	Dead	65	female	23.37258	yes	Non-Papill	Stage ii	High Grad	M0	N0	T2b	YES	No	NA
TCGA-CU-A0YO	4.966667	Dead	84	male	NA	yes	Non-Papill	Stage iv	High Grad	MX	N2	T3a	YES	No	NO
TCGA-G2-A2EC	23.2	Dead	58	female	27.34375	yes	Non-Papill	Stage ii	High Grad	M0	N0	NA	NA	No	NO
TCGA-GD-A76B	7.466667	Dead	86	female	26.07897	yes	Papillary	Stage ii	High Grad	MX	N0	T2b	NO	No	NA
TCGA-E7-A3Y1	5.433333	Alive	57	male	27.71769	yes	Papillary	Stage ii	Low Grade	M0	N0	NA	NA	No	YES
TCGA-BT-A0YX	13.33333	Dead	70	female	NA	yes	Non-Papill	Stage iii	High Grad	M0	N0	T3b	YES	No	NO
TCGA-BT-A2LA	17.4	Alive	54	male	NA	yes	Non-Papill	Stage iii	High Grad	M0	N0	T3a	NO	No	NO
TCGA-CF-A5U8	13.3	Alive	59	male	19.62826	yes	Papillary	Stage ii	Low Grade	M0	N0	T2	NA	No	NO
TCGA-DK-A1AA	19.26667	Alive	57	male	32.55204	yes	Papillary	Stage iii	High Grad	M0	N0	T3	NA	No	NO
TCGA-CF-A7I0	12.26667	Alive	54	male	20.32225	yes	Papillary	Stage ii	Low Grade	M0	N0	T2	NA	No	NO
TCGA-G2-A2EK	16.16667	Alive	57	male	48.73253	yes	Non-Papill	Stage ii	High Grad	M0	N0	NA	NA	No	NO
TCGA-DK-A2I6	88.53333	Alive	81	male	27.93208	yes	Non-Papill	Stage iv	High Grad	M0	N1	T2b	YES	No	NO
TCGA-G2-AA3B	66.93333	Alive	75	female	27.25089	yes	Non-Papill	Stage ii	High Grad	M0	N0	T2	YES	No	NO
TCGA-G2-A3IE	20.4	Dead	51	male	29.61599	yes	Non-Papill	Stage ii	High Grad	MX	NX	NA	NA	No	NO
TCGA-FD-A43N	23.3	Alive	76	male	35.75128	yes	Non-Papill	Stage iii	High Grad	MX	N0	T3a	NA	No	NA
TCGA-DK-A3IS	50.96667	Alive	68	male	23.87829	yes	Papillary	Stage ii	High Grad	M0	N0	T2a	NA	No	NO
TCGA-G2-AA3F	29.76667	Alive	77	male	30.40529	yes	Non-Papill	Stage iv	High Grad	M0	N1	T3	YES	No	NO
TCGA-FJ-A3Z9	12.83333	Dead	72	male	31.35358	yes	Papillary	not reporte	High Grad	M0	N0	TX	NO	No	NO
TCGA-MV-A51V	13.66667	Alive	75	male	NA	yes	Papillary	Stage iii	High Grad	M0	N0	T3a	NO	No	NO
TCGA-UY-A78O	77.06667	Alive	75	female	46.29451	yes	Non-Papill	Stage ii	High Grad	MX	N0	T2	NO	No	NO
TCGA-GC-A3RD	14.26667	Alive	83	female	28.80441	yes	Non-Papill	Stage iii	High Grad	M0	N0	T3a	NO	No	NO
TCGA-E5-A2PC	44.2	Alive	61	female	27.05515	yes	Non-Papill	Stage iv	High Grad	MX	N1	T2b	NA	No	NO
TCGA-UY-A8OD	114.4	Alive	68	female	NA	no	Non-Papill	Stage ii	High Grad	MX	N0	T2b	NA	No	NO
TCGA-CU-A72E	13.76667	Dead	76	male	42.28395	yes	Non-Papill	Stage iv	High Grad	M0	N2	T3b	YES	Yes	NO
TCGA-GV-A3JZ	20.1	Alive	55	male	25.08286	yes	Non-Papill	Stage iv	High Grad	MX	N3	T4a	YES	No	NO
TCGA-GD-A2C5	27.06667	Alive	53	female	24.32323	yes	Papillary	Stage iv	High Grad	MX	N2	T3a	YES	No	NA
TCGA-DK-A6AW	54.03333	Alive	70	male	31.74179	yes	Non-Papill	Stage ii	High Grad	M0	N0	T2a	NO	No	NO
TCGA-BT-A42F	28.8	Alive	64	male	31.48148	yes	Non-Papill	Stage iv	High Grad	MX	N1	T4a	YES	No	NO
TCGA-FD-A6TI	9.8	Dead	73	male	45.36941	yes	Non-Papill	Stage iv	High Grad	MX	N1	T4b	YES	No	NO
TCGA-4Z-AA84	15.33333	Alive	61	male	22.86253	yes	Papillary	Stage iv	High Grad	M1	N2	T3a	YES	No	NA
TCGA-CU-A0YR	15.33333	Dead	83	male	23.85107	yes	Non-Papill	Stage iv	High Grad	M0	N2	T2	YES	No	NO
TCGA-FD-A3N6	28.36667	Alive	43	female	49.33492	yes	Non-Papill	Stage ii	High Grad	MX	N0	T2b	NA	No	NO
TCGA-BT-A3PK	10.1	Dead	80	male	27.77778	yes	Papillary	Stage ii	High Grad	MX	N0	T2b	NO	No	NO
TCGA-FD-A6TG	3.1	Dead	73	male	25.10239	yes	Non-Papill	Stage iv	High Grad	MX	N2	T3a	YES	No	NO
TCGA-CF-A27C	14.16667	Alive	52	male	20.41522	yes	Papillary	Stage iii	High Grad	M0	N0	T3	NA	No	YES
TCGA-GV-A3QF	20.56667	Dead	79	male	23.62029	yes	Papillary	Stage iv	High Grad	MX	N2	T3b	YES	No	NO
TCGA-DK-A6B0	77.66667	Alive	61	male	28.32658	yes	Papillary	Stage ii	High Grad	M0	N0	T2b	NO	No	NO
TCGA-XF-A8HB	45.66667	Alive	50	female	23.83673	yes	Papillary	Stage ii	High Grad	MX	N0	T2b	NO	No	NO
TCGA-BT-A42C	29.1	Alive	64	male	25.5367	yes	Papillary	Stage ii	High Grad	M0	N0	NA	NO	No	NO
TCGA-5N-A9KM	17.66667	Dead	73	female	24.97704	yes	Non-Papill	Stage iii	High Grad	MX	N0	T4a	NO	No	NA
TCGA-GD-A3OP	2.133333	Alive	84	female	NA	no	Papillary	Stage iv	High Grad	MX	N2	T4a	YES	No	NA
TCGA-XF-A8HC	6.666667	Dead	79	male	24.83576	yes	Papillary	Stage iv	High Grad	MX	N2	T3a	YES	No	NA
TCGA-CF-A1HR	12.96667	Alive	62	male	20.61925	yes	Non-Papill	Stage iii	High Grad	M0	N0	T3	NA	No	NO
TCGA-E7-A678	26.6	Alive	55	male	21.48438	yes	Non-Papill	Stage iii	Low Grade	M0	N0	T3	NO	No	NO
TCGA-K4-A54R	28.06667	Alive	59	male	NA	no	Papillary	Stage ii	High Grad	MX	N0	T2b	NO	No	NO
TCGA-GC-A3OO	16.03333	Alive	79	male	38.81872	yes	Non-Papill	Stage ii	High Grad	M0	N0	T2b	NO	No	NO
TCGA-BT-A3PH	4.733333	Dead	76	male	NA	yes	Non-Papill	Stage iv	High Grad	MX	N2	T3b	YES	No	NA
TCGA-GV-A3QH	8.6	Dead	67	male	26.87854	yes	Non-Papill	Stage ii	High Grad	MX	NX	NA	NA	No	NO
TCGA-FD-A5BS	54.63333	Alive	68	male	NA	yes	Non-Papill	Stage iii	High Grad	MX	N0	T3b	NA	No	NO
TCGA-UY-A78P	79.33333	Alive	78	female	NA	yes	Non-Papill	Stage ii	High Grad	MX	N0	T2	NO	No	NO
TCGA-ZF-AA51	57.13333	Alive	69	female	24.32323	yes	Papillary	Stage ii	High Grad	M0	NX	T2	NA	No	NO
TCGA-LT-A8JT	21.36667	Alive	69	female	21.2963	yes	Papillary	Stage ii	High Grad	M0	N0	T2a	NO	No	NO
TCGA-ZF-AA4U	8.733333	Dead	70	male	29.32099	yes	Non-Papill	Stage iii	High Grad	MX	NX	T4a	YES	No	NA
TCGA-GV-A3JW	21.63333	Alive	74	male	29.40292	yes	Non-Papill	Stage ii	High Grad	MX	NX	T2	NA	No	YES
TCGA-GV-A6ZA	23.03333	Alive	54	male	25.99244	yes	Papillary	Stage ii	High Grad	MX	N0	T2b	NA	No	NO
TCGA-ZF-AA5P	12.4	Alive	65	male	26.81222	yes	Non-Papill	Stage iv	High Grad	M0	N2	T2b	NA	No	NO
TCGA-GU-A767	4.8	Dead	81	male	22.79036	yes	Papillary	Stage iv	High Grad	MX	N2	T3b	YES	No	NA
TCGA-G2-A2EJ	48.66667	Alive	56	female	28.11533	yes	Non-Papill	Stage ii	High Grad	M0	N0	NA	NA	No	NO
TCGA-DK-A1AD	114	Alive	69	male	26.05243	no	Non-Papill	Stage iv	High Grad	M0	N2	T3b	NO	No	NO
TCGA-E7-A519	16.93333	Alive	72	male	25.50362	yes	Papillary	Stage ii	High Grad	M0	NX	T2b	NA	No	NO
TCGA-G2-A2EL	27.3	Dead	77	male	27.77691	yes	Non-Papill	Stage ii	High Grad	M0	N0	NA	NA	No	NO
TCGA-E7-A7DU	0.933333	Alive	73	male	36.75365	yes	Papillary	Stage iii	Low Grade	M0	N0	T3	NO	No	NA
TCGA-FD-A3SL	23.73333	Dead	60	male	34.63824	yes	Non-Papill	Stage iv	High Grad	M1	N2	T4a	YES	No	NO
TCGA-YC-A89H	19.1	Alive	78	female	20.92747	yes	Non-Papill	Stage ii	High Grad	MX	NA	NA	NO	No	NO
TCGA-4Z-AA87	48.46667	Alive	72	male	21.64412	yes	Non-Papill	Stage iii	High Grad	M0	N0	T4a	NO	No	NO
TCGA-C4-A0F1	2.966667	Alive	71	male	26.47211	yes	Non-Papill	Stage iii	High Grad	M0	N0	T3b	NA	No	NA
TCGA-4Z-AA7S	35.46667	Dead	66	male	NA	yes	Non-Papill	Stage iii	High Grad	M0	N0	T4a	NO	No	NA
TCGA-DK-A3IT	21.6	Alive	62	male	33.57651	yes	Non-Papill	Stage iii	High Grad	M0	N0	T3	NA	No	NO
TCGA-FD-A3NA	61.5	Alive	60	male	25.54026	yes	Non-Papill	Stage ii	High Grad	MX	N0	T2b	NA	No	NO
TCGA-K4-A83P	16.5	Alive	77	male	33.41241	yes	Non-Papill	Stage iv	NA	MX	N1	T4a	YES	No	NO
TCGA-4Z-AA81	42.33333	Dead	80	male	26.98962	yes	Papillary	Stage ii	High Grad	M0	N0	T2b	NO	No	NO
TCGA-FD-A5BR	27.1	Alive	57	male	37.34568	yes	Non-Papill	Stage ii	High Grad	MX	N0	T2b	NA	No	NO
TCGA-UY-A78M	23	Dead	81	female	23.49524	yes	Non-Papill	Stage iv	High Grad	MX	N2	T2	NO	No	NA
TCGA-CU-A3YL	30.2	Alive	67	male	29.02531	yes	Papillary	Stage iii	High Grad	M0	N0	T4a	NO	No	YES
TCGA-DK-A6AV	65.06667	Alive	82	female	23.8054	yes	Papillary	Stage ii	High Grad	M0	N0	T2a	NO	No	NO
TCGA-FT-A3EE	3.3	Dead	80	female	29.34004	yes	Papillary	Stage iii	High Grad	MX	N0	T4a	YES	No	NA
TCGA-FD-A43X	3.666667	Alive	84	male	24.24242	yes	Papillary	Stage ii	High Grad	MX	N0	T2a	NA	No	NO
TCGA-XF-A9T5	67.56667	Alive	78	female	27.88519	yes	Non-Papill	Stage iv	High Grad	MX	N1	T3a	NO	No	NO
TCGA-FD-A62O	7.2	Dead	74	male	19.40936	yes	Non-Papill	Stage iv	High Grad	MX	N2	T3a	NA	No	NO
TCGA-CF-A8HY	11.5	Alive	59	male	21.79945	yes	Papillary	Stage ii	High Grad	M0	N0	T2	YES	No	NO
TCGA-BT-A20T	15.1	Dead	63	male	NA	yes	Non-Papill	Stage iv	High Grad	M0	N1	T3b	NA	No	NO

TCGA-FD-A5BV	5.433333	Dead	47	female	17.90886	yes	Non-Papill	Stage iii	High Grad	MX	N0	T3b	NA	No	NA
TCGA-XF-AAMR	93	Alive	48	male	25.79592	yes	Non-Papill	Stage iii	High Grad	MX	N0	T3b	NO	No	NO
TCGA-GV-A3JV	14.46667	Dead	66	male	25.71643	yes	Non-Papill	Stage iv	High Grad	MX	N1	T3b	YES	No	NO
TCGA-FD-A3SN	29.56667	Alive	79	male	24.22145	yes	Papillary	Stage iii	High Grad	MX	N0	T3b	NA	No	NO
TCGA-FD-A6TE	12.53333	Alive	54	male	20.06173	yes	Non-Papill	Stage iv	High Grad	MX	N2	T3a	YES	No	NO
TCGA-FD-A3SJ	24.63333	Dead	59	male	24.93372	yes	Non-Papill	Stage iv	High Grad	MX	N2	T2b	NA	No	NO
TCGA-CU-A5W6	1.866667	Dead	70	male	26.44898	yes	Non-Papill	Stage iii	High Grad	M0	N0	T4a	YES	No	NA
TCGA-CF-A9FH	79.33333	Alive	78	female	NA	yes	Non-Papill	Stage ii	High Grad	MX	N0	T2	NO	No	NO
TCGA-BT-A2LB	16.4	Dead	73	female	NA	yes	Non-Papill	Stage iii	High Grad	M0	N0	T3a	NA	No	NO
TCGA-GC-A6I1	72.56667	Alive	59	female	30.11621	yes	Papillary	Stage ii	High Grad	MX	N0	T2a	NO	No	NO
TCGA-ZF-A9R3	31.63333	Dead	53	female	46.38219	yes	Papillary	Stage ii	High Grad	M0	NX	T2	NA	No	NO
TCGA-4Z-AA83	67.46667	Alive	52	male	30.15916	yes	Papillary	Stage ii	High Grad	M0	N0	T2a	NO	No	NO
TCGA-E7-A97Q	8.2	Dead	60	female	23.66524	yes	Non-Papill	Stage iv	High Grad	MX	N3	T4a	YES	No	NO
TCGA-FD-A43U	21.2	Alive	70	male	27.1731	yes	Non-Papill	Stage iv	High Grad	MX	N2	T4a	NA	No	NO
TCGA-HQ-A5ND	9.133333	Dead	78	male	23.52941	yes	NA	Stage iv	High Grad	M0	N1	T3b	YES	No	NO
TCGA-XF-A8HH	1.9	Dead	61	female	27.09925	yes	Non-Papill	Stage iv	High Grad	M0	N2	T3b	YES	No	NA
TCGA-E7-A4XJ	2.266667	Dead	66	male	22.13674	yes	Non-Papill	Stage ii	High Grad	M0	N0	T2	NO	No	NO
TCGA-CF-A9FM	13.26667	Alive	50	male	19.4674	yes	Papillary	Stage i	High Grad	M0	N0	T1	YES	No	NO
TCGA-GC-A3BM	21.7	Dead	70	male	28.08163	yes	Non-Papill	Stage ii	High Grad	M0	N0	T2b	YES	No	NO
TCGA-G2-AA3D	71.3	Alive	60	male	36.59246	yes	Non-Papill	Stage iv	High Grad	M0	N2	T3	YES	No	NO
TCGA-E7-A3X6	30.13333	Dead	70	male	30.79585	yes	Papillary	Stage ii	Low Grade	M0	NX	T2	NA	No	NO
TCGA-ZF-AA5H	29.9	Alive	60	female	30.86304	yes	Non-Papill	Stage iv	High Grad	M0	N2	T3b	YES	No	NO
TCGA-XF-A9SU	6.066667	Dead	74	female	24.56033	yes	Papillary	Stage iv	High Grad	MX	N1	T3b	YES	No	NA
TCGA-E7-A8O7	15.53333	Alive	52	male	20.54989	yes	Papillary	Stage ii	Low Grade	M0	N0	T2	NO	No	NO
TCGA-DK-A3IV	9.8	Dead	60	male	28.86696	yes	Non-Papill	Stage ii	High Grad	M0	NX	NA	NA	No	NO
TCGA-K4-A3WV	21.53333	Alive	77	female	NA	yes	Non-Papill	Stage ii	High Grad	MX	N0	T2b	NO	No	NO
TCGA-CF-A3MG	12.3	Alive	48	male	20.2449	yes	Papillary	Stage ii	Low Grade	M0	N0	T2	NA	No	NO
TCGA-ZF-A9R2	21.4	Alive	75	male	20.10916	yes	Papillary	Stage ii	High Grad	M0	N0	T2b	YES	No	NO
TCGA-FJ-A3ZE	10.8	Dead	65	male	32.548	yes	Non-Papill	Stage iv	High Grad	M1	N3	NA	YES	No	YES
TCGA-FJ-A3ZF	17.46667	Alive	73	male	30.23432	yes	Papillary	Stage iii	High Grad	M0	N0	NA	NO	Yes	NO
TCGA-FD-A5C0	18.33333	Dead	61	male	26.88172	yes	Non-Papill	Stage iv	High Grad	MX	N2	T3a	NA	No	NO
TCGA-LT-A5Z6	15.8	Alive	56	male	25.68007	yes	Non-Papill	Stage ii	High Grad	MX	NX	NA	YES	No	NO
TCGA-YF-AA3L	12.13333	Alive	47	female	33.44382	yes	Non-Papill	Stage ii	High Grad	MX	N0	T2b	YES	No	NO
TCGA-XF-AAN0	57.26667	Dead	68	male	25.35154	yes	Non-Papill	Stage iv	High Grad	M0	N2	T4a	YES	No	NA
TCGA-DK-A3WX	10.7	Dead	67	female	28.04038	yes	Non-Papill	Stage iii	High Grad	M0	N0	T3	YES	No	NO
TCGA-K4-A4AC	9.266667	Dead	83	male	NA	yes	Non-Papill	Stage ii	High Grad	MX	N0	T2b	NO	No	YES
TCGA-E7-A6MD	4.3	Alive	66	male	NA	no	Non-Papill	Stage iv	High Grad	M0	N1	T4a	YES	No	NA
TCGA-ZF-AA52	35.9	Dead	70	male	23.50356	yes	Papillary	Stage iii	High Grad	MX	NX	T3a	NA	No	NA
TCGA-XF-AAN3	87.5	Alive	76	male	19.84127	yes	Non-Papill	Stage iv	High Grad	MX	N2	T3b	YES	No	NO
TCGA-UY-A78K	17.86667	Dead	60	male	NA	yes	Papillary	Stage iv	High Grad	MX	N2	NA	NA	No	NA
TCGA-CU-A3KJ	18.73333	Alive	75	male	23.93948	yes	Non-Papill	Stage iii	High Grad	M0	N0	T3b	NO	No	NO
TCGA-FD-A3N5	22.83333	Dead	69	male	NA	yes	Non-Papill	Stage ii	High Grad	MX	N0	T2b	NO	No	NO
TCGA-FD-A3SM	18.23333	Dead	70	male	27.72748	yes	Papillary	Stage iv	High Grad	M1	N2	T3a	NA	No	NO
TCGA-DK-A1A6	67.33333	Alive	53	male	32.71409	yes	Non-Papill	Stage iv	High Grad	M0	N1	T2a	YES	No	NO
TCGA-ZF-AA4X	68.13333	Alive	56	male	23.83673	yes	Non-Papill	Stage ii	High Grad	M0	NX	T2	NO	No	YES
TCGA-XF-A8HI	18.13333	Dead	57	female	32.59637	yes	Papillary	Stage iv	High Grad	MX	N2	T2b	YES	No	NA
TCGA-DK-A1A3	22.16667	Dead	60	male	22.69402	yes	NA	Stage iv	High Grad	M0	N2	T3	YES	No	NA
TCGA-K4-A6FZ	1.833333	Alive	75	female	25.61728	yes	Non-Papill	Stage iii	High Grad	MX	N0	T3a	YES	No	NA
TCGA-XF-A9T8	13.93333	Dead	64	male	24.61521	yes	Non-Papill	Stage iii	High Grad	MX	N0	T3b	YES	No	NA
TCGA-G2-A2ES	33.46667	Dead	85	male	29.33384	yes	Non-Papill	Stage ii	High Grad	M0	N0	T3b	NA	No	NO
TCGA-XF-AAMQ	72.56667	Alive	59	female	30.11621	yes	Papillary	Stage ii	High Grad	MX	N0	T2a	NO	No	NO
TCGA-ZF-A9R4	30.7	Alive	83	male	NA	yes	Non-Papill	Stage ii	High Grad	M0	NX	T2	YES	No	NO
TCGA-4Z-AA7O	17.06667	Alive	64	male	25.76571	yes	Papillary	Stage ii	High Grad	M0	N0	T2a	YES	No	NO
TCGA-XF-AAN1	31.36667	Dead	75	female	32.59637	yes	Papillary	Stage iii	High Grad	M0	N0	T4a	NO	No	NA
TCGA-DK-A1AG	15.83333	Alive	65	male	26.03878	yes	Papillary	Stage iii	High Grad	M0	N0	T3	NA	No	NO
TCGA-GC-A6I3	9.066667	Alive	60	male	32.71409	yes	Papillary	Stage iv	High Grad	M1	N2	T3a	NA	No	NO
TCGA-4Z-AA7R	17.4	Dead	73	male	25.03992	yes	Papillary	Stage iv	High Grad	M0	N0	T4b	YES	No	NO
TCGA-ZF-A9RL	90.1	Alive	61	male	NA	yes	Papillary	Stage ii	High Grad	MX	NA	NA	NA	No	NO
TCGA-4Z-AA89	34.3	Alive	60	male	31.43591	yes	Papillary	Stage iv	High Grad	M0	N0	T4b	NA	No	NO
TCGA-PQ-A6FI	12.4	Alive	70	male	30.10381	yes	Non-Papill	Stage ii	High Grad	MX	N0	T2a	NO	No	NA
TCGA-FD-A3B3	32.46667	Dead	74	female	36.02402	yes	Papillary	Stage iii	High Grad	MX	N0	T3	NO	No	NO
TCGA-DK-A3IM	8.266667	Dead	76	male	30.21142	yes	Papillary	Stage iii	High Grad	M0	N0	T3	NA	No	NA
TCGA-GC-A3WC	18	Alive	80	female	26.83518	yes	Non-Papill	Stage iii	High Grad	MX	N0	T3	YES	No	NO
TCGA-ZF-AA4W	61	Alive	56	male	27.45289	yes	Non-Papill	Stage iii	High Grad	MX	N0	T3b	YES	No	NO
TCGA-4Z-AA7Q	17	Dead	79	male	24.16716	yes	Non-Papill	Stage iii	High Grad	M0	NX	T3a	NO	No	NA
TCGA-E7-A4IJ	22.46667	Dead	56	male	18.90204	yes	Papillary	Stage ii	High Grad	M0	NX	T2b	YES	No	NO
TCGA-G2-A3IB	7.333333	Dead	66	male	30.70707	yes	Non-Papill	Stage ii	High Grad	MX	NX	NA	NA	No	NO
TCGA-GV-A40G	19.33333	Alive	77	male	25.30864	yes	Non-Papill	Stage ii	High Grad	MX	N0	T2a	NO	No	NO
TCGA-FD-A3B7	4.066667	Dead	66	male	18.96007	yes	Non-Papill	Stage iii	High Grad	MX	N0	T3a	NA	No	NO
TCGA-GC-A3I6	21	Dead	45	male	27.37966	yes	Non-Papill	Stage iii	High Grad	M0	N0	T3a	NO	No	NO
TCGA-UY-A9PE	6.3	Alive	86	male	29.7619	yes	Non-Papill	Stage iv	High Grad	MX	N2	T2b	NA	No	NO
TCGA-2F-A9KP	12.13333	Dead	66	male	37.5766	yes	Non-Papill	Stage iv	High Grad	MX	N2	T3a	NA	No	NA
TCGA-ZF-AA4T	19.96667	Dead	65	male	28.73175	yes	Non-Papill	Stage iv	High Grad	MX	N2	T4	NO	No	NA
TCGA-XF-A9SV	12.93333	Dead	82	male	22.83951	yes	Papillary	Stage iv	High Grad	M1	N2	T4a	YES	No	NA
TCGA-E7-A7PW	13.86667	Alive	63	male	19.03114	yes	Non-Papill	Stage iii	High Grad	M0	NX	T3a	NO	No	NO
TCGA-BT-A2LD	20.76667	Dead	78	female	38.57876	yes	Non-Papill	Stage iv	High Grad	M0	N1	T3a	YES	No	NO
TCGA-ZF-A9R1	25.76667	Dead	81	male	30.48669	yes	Non-Papill	Stage iv	High Grad	M0	N1	T3b	YES	No	NO
TCGA-XF-A8HE	127.2333	Alive	47	male	25.24934	yes	Non-Papill	Stage iii	High Grad	MX	N0	T3b	NO	No	NO
TCGA-4Z-AA80	0.633333	Dead	73	male	29.03674	yes	Papillary	Stage ii	High Grad	M0	N0	T2a	NO	No	NA
TCGA-LC-A66R	15.53333	Alive	78	male	20.98765	yes	Papillary	Stage iv	High Grad	MX	N2	T4a	YES	No	NO
TCGA-K4-AAQO	11.96667	Alive	56	male	30.8642	yes	Non-Papill	Stage iii	High Grad	MX	N0	T3a	YES	Yes	NA
TCGA-ZF-A9RE	3.533333	Dead	78	female	22.77319	yes	Papillary	Stage ii	High Grad	MX	NA	NA	NO	No	NA
TCGA-2F-A9KO	24.46667	Dead	63	male	21.71807	yes	Non-Papill	Stage iv	High Grad	M0	N1	T3	NO	No	NO
TCGA-BT-A20J	19.3	Dead	75	male	NA	yes	Non-Papill	Stage ii	High Grad	MX	N0	T2b	NO	No	NO
TCGA-GU-A42P	11.06667	Dead	72	male	23.88844	yes	Papillary	Stage iv	High Grad	M0	N1	T3a	YES	No	NO
TCGA-FD-A43S	15.16667	Alive	71	female	24.74745	yes	Non-Papill	Stage iii	High Grad	MX	N0	T3b	NA	No	NO
TCGA-PQ-A6FN	16.9	Alive	78	female	24.22145	yes	Non-Papill	Stage iii	High Grad	MX	N0	T3a	NA	No	NA
TCGA-E7-A5KF	0.666667	Alive	67	male	22.26563	yes	Non-Papill	Stage ii	Low Grade	M0	N0	T2a	NO	No	NA
TCGA-UY-A9PA	35.73333	Alive	48	male	24.4898	yes	Non-Papill	Stage iii	High Grad	MX	N0	T3a	NO	No	NO
TCGA-GU-A764	20.33333	Alive	66	male	22.0384	yes	Non-Papill	Stage ii	High Grad	MX	N0	T2b	NO	No	NO
TCGA-E7-A7DV	1.233333	Alive	44	male	23.73866	no	Papillary	Stage iv	High Grad	MX	N3	T4	YES	No	NA
TCGA-DK-A2I2	7.9	Dead	63	female	29.66655	yes	Non-Papill	Stage iv	High Grad	M0	N3	T3	YES	No	NO
TCGA-ZF-A9RF	64.96667	Alive	74	male	NA	yes	Non-Papill	Stage ii	High Grad	M0	NX	T1	NO	No	NO
TCGA-2F-A9KW	8.466667	Dead	67	female	25.0995	yes	Non-Papill	Stage iii	High Grad	MX	N0	T3b	NO	No	NO
TCGA-SY-A9G0	33.6	Dead	82	male	28.3737	yes	Papillary	Stage iv	High Grad	M0	N1	T4	YES	No	NO
TCGA-UY-A78N	88.03333	Dead	80	male	25.05931	yes	Non-Papill	Stage iv	High Grad	MX	N1	T2	NO	No	NO
TCGA-GC-A3YS	25.26667	Alive	61	male	23.66691	yes	Non-Papill	Stage iv	High Grad	MX	N1	T3a	YES	No	NO
TCGA-4Z-AA7Y	50.73333	Alive	60	male	28.306	yes	Papillary	Stage ii	High Grad	M0	N0	T2a	NO	No	NO
TCGA-FD-A3B5	9.066667	Dead	86	male	22.95586	yes	Non-Papill	Stage iv	High Grad	MX	N1	T2b	NA	No	NO
TCGA-ZF-AA4V	60.2	Alive	66	male	27.13141	yes	Non-Papill	Stage iii	High Grad	M0	N0	T3b	NA	No	NO
TCGA-K4-A6MB	15.63333	Alive	64	male	27.77427	yes	Non-Papill	Stage iv	High Grad	MX	N1	T3b	YES	No	NO
TCGA-CF-A5UA	12.16667	Alive	67	male	17.99308	yes	Papillary	Stage ii	Low Grade	M0	N0	T2	NA	No	NO
TCGA-YC-A8S6	9.766667	Alive	71	male	38.20408	yes	Papillary	Stage ii	High Grad	MX	N0	T2a	NO	No	NO
TCGA-GC-A3RC	16.13333	Alive	59	male	36.2449	yes	Non-Papill	Stage ii	High Grad	M0	N0	T2b	YES	No	NO
TCGA-E7-A541	25.93333	Dead	66	male	NA	no	Papillary	Stage ii	High Grad	MX	N0	T2b	NO	No	NO
TCGA-FD-A5BU	19.6	Alive	76	female	23.30668	yes	Non-Papill	Stage ii	High Grad	MX	N0	T2b	NA	No	NO
TCGA-ZF-A9R5	36.33333	Alive	59	male	22.38631	yes	Papillary	Stage iii	High Grad	M0	N0	T3	NA	No	NO
TCGA-BT-A20N	26.5	Dead	72	male	NA	yes	Non-Papill	Stage iii	High Grad	MX	N0	T3a	NA	No	NO
TCGA-GU-A42R	19.23333	Dead	68	male	31.48148	yes	Papillary	Stage iv	High Grad	MX	N2	T4a	YES	No	NO
TCGA-UY-A78L	37.56667	Alive	62	male	31.83391	no	Papillary	Stage iv	High Grad	MX	N1	NA	NO	No	NO
TCGA-FD-A6TD	12.86667	Dead	77	male	39.73261	yes	Non-Papill	Stage iii	High Grad	MX	N0	T3a	NO	No	NO
TCGA-FD-A5C1	59.73333	Alive	61	female	35.98616	yes	Non-Papill	Stage iii	High Grad	MX	N0	T3b	NA	No	NO
TCGA-ZF-A9RN	20.5	Dead	67	female	29.04657	yes	Non-Papill	Stage iii	High Grad	M0	NX	T3b	YES	No	NA
TCGA-UY-A8OC	16.76667	Dead	66	male	35.98616	yes	Papillary	Stage iv	High Grad	M0	N1	T4	YES	No	NO

TCGA-FD-A6TA	63.73333	Alive	58	male	27.14304	yes	Papillary	Stage iv	High Grad	MX	N2	T3b	NA	No	NO
TCGA-E5-A4U1	39.36667	Alive	72	male	33.46403	yes	Papillary	Stage ii	High Grad	M0	N0	T2b	NO	No	NO
TCGA-FD-A62P	6.366667	Dead	76	male	29.53099	yes	Non-Papill	Stage ii	High Grad	MX	N0	T2b	NA	Yes	NA
TCGA-BT-A20Q	19.76667	Dead	73	male	NA	yes	Non-Papill	Stage iv	High Grad	M0	N2	T3b	NA	No	NO
TCGA-ZF-A9RM	48.5	Alive	70	male	29.0688	yes	Papillary	Stage ii	High Grad	MX	N0	T0	NO	No	NO
TCGA-DK-A1AB	16.93333	Dead	74	female	33.91251	yes	Non-Papill	Stage iv	High Grad	M0	N2	T4a	YES	No	NO
TCGA-ZF-A9R7	22.16667	Alive	76	female	26.10656	yes	Non-Papill	Stage ii	High Grad	M0	NX	NA	NO	No	NO
TCGA-DK-A6B2	15.9	Alive	70	male	34.15366	yes	Non-Papill	Stage iv	High Grad	M0	N1	T3	YES	No	NO
TCGA-ZF-A9RC	95.6	Alive	77	male	32.32653	yes	Non-Papill	Stage iii	High Grad	MX	N0	T3a	NO	No	NO
TCGA-GC-A3RB	19.4	Alive	54	male	NA	yes	Non-Papill	Stage iii	High Grad	M0	N0	T3b	NA	No	NO
TCGA-XF-AAMJ	55.66667	Dead	70	male	22.31328	yes	Non-Papill	Stage iii	High Grad	M0	N0	T3b	YES	No	NA
TCGA-ZF-AA53	58.7	Alive	60	male	35.03346	yes	Non-Papill	Stage ii	High Grad	M0	NX	T2	NA	No	NO
TCGA-FD-A6TC	6.233333	Alive	79	female	22.26563	yes	Non-Papill	Stage iii	High Grad	MX	N0	T4a	NO	No	NO
TCGA-XF-A9SL	67.33333	Dead	69	male	29.06122	yes	Non-Papill	Stage iv	High Grad	MX	N2	T3a	YES	No	NA
TCGA-GU-AATP	33.43333	Alive	74	male	34.47772	yes	Papillary	Stage iv	High Grad	MX	N2	T2	YES	No	NO
TCGA-DK-A3IU	23.53333	Dead	58	male	31.40245	yes	NA	Stage ii	High Grad	M0	N0	T2b	NA	No	NO
TCGA-G2-A2EO	60.13333	Dead	69	male	26.92911	yes	Non-Papill	Stage iii	High Grad	M0	N0	T3a	NA	No	NO
TCGA-FD-A43P	27.73333	Alive	74	male	47.34251	yes	Non-Papill	Stage ii	High Grad	MX	N0	T2a	NA	No	NO
TCGA-GD-A3OQ	3.166667	Alive	48	male	NA	yes	Papillary	Stage iv	High Grad	MX	N1	T4a	YES	No	NA
TCGA-GD-A6C6	2.233333	Alive	64	male	19.26717	yes	Papillary	Stage iii	High Grad	MX	N0	T3a	YES	No	NA
TCGA-FD-A62N	2.733333	Alive	69	male	25.35926	yes	Non-Papill	Stage iii	High Grad	MX	N0	T3b	NA	No	NA
TCGA-FD-A3SQ	47.43333	Dead	62	male	23.16743	yes	Non-Papill	Stage iv	High Grad	MX	N2	T3a	NA	No	NO
TCGA-XF-AAML	7.733333	Dead	75	male	38.94457	yes	Papillary	Stage ii	High Grad	MX	N0	T2b	NO	No	NA
TCGA-XF-AAMT	3	Dead	75	female	23.4375	yes	Non-Papill	Stage iv	High Grad	MX	N2	T3b	YES	No	NA
TCGA-FD-A3SS	13.03333	Dead	66	male	29.0688	yes	Non-Papill	Stage iv	High Grad	MX	N3	T4	YES	No	NO
TCGA-UY-A9PD	18.06667	Alive	80	male	22.72044	yes	Non-Papill	Stage iii	High Grad	MX	N0	T3a	NA	No	NO
TCGA-GU-A766	16	Alive	62	male	33.30612	yes	Non-Papill	Stage ii	High Grad	MX	N0	T2a	NO	No	NO
TCGA-XF-A9T0	26.63333	Alive	68	male	25.88057	yes	Non-Papill	Stage iii	High Grad	MX	N0	T3b	NO	No	NO
TCGA-K4-A3WU	18.83333	Dead	60	male	26.57589	yes	Non-Papill	Stage iv	High Grad	MX	N1	T3a	YES	No	NA
TCGA-G2-A3VY	17.86667	Alive	66	male	20.71611	yes	Non-Papill	Stage ii	High Grad	NA	NA	NA	NO	No	NO
TCGA-XF-A9T4	16.5	Dead	48	male	24.69136	yes	Non-Papill	Stage iv	High Grad	MX	N1	T2b	NO	No	NA
TCGA-H4-A2HQ	19.66667	Alive	64	female	22.86253	yes	Non-Papill	Stage iv	High Grad	M1	NX	NA	NA	No	NO
TCGA-4Z-AA7M	16.5	Alive	65	male	20.38043	yes	Non-Papill	Stage iii	High Grad	M0	N0	T3a	NO	No	NO
TCGA-FD-A6TK	11	Alive	60	male	22.18935	yes	Non-Papill	Stage iii	High Grad	MX	N0	T3a	NO	No	NO
TCGA-BT-A20R	5.133333	Dead	79	female	NA	yes	Non-Papill	Stage iv	High Grad	M0	N1	T3b	NA	No	NO
TCGA-CU-A0YN	13.1	Dead	60	male	21.27257	yes	Non-Papill	Stage iii	High Grad	M0	N0	T3a	NA	No	NO
TCGA-YF-AA3M	13.83333	Alive	57	male	27.28175	yes	Non-Papill	Stage ii	High Grad	MX	NX	T2a	NO	Yes	NO
TCGA-4Z-AA82	51.86667	Dead	59	male	19.69267	yes	Non-Papill	Stage iv	High Grad	M0	N1	T2a	NO	No	NO
TCGA-XF-AAMZ	44.93333	Dead	81	female	20.3125	yes	Papillary	Stage iv	High Grad	MX	N2	T3a	YES	No	NA
TCGA-UY-A9PH	52.03333	Alive	73	male	23.63403	yes	Non-Papill	Stage ii	High Grad	MX	N0	T2b	NA	Yes	NO
TCGA-FD-A6TB	19.06667	Alive	82	male	21.45329	yes	Non-Papill	Stage iii	High Grad	MX	N0	T3a	NO	Yes	NO
TCGA-XF-A8HD	98.8	Alive	77	male	23.76543	yes	Non-Papill	Stage iii	High Grad	MX	N0	T3a	NO	No	NO
TCGA-BT-A3PJ	26.3	Alive	76	male	NA	yes	Papillary	Stage iii	High Grad	M0	N0	T3b	NO	No	NO
TCGA-BT-A20W	8.466667	Dead	71	male	NA	no	Non-Papill	Stage ii	High Grad	M0	N0	T2b	NA	No	NO
TCGA-S5-A6DX	1.866667	Dead	83	male	30.04082	yes	Non-Papill	Stage iv	High Grad	MX	N2	T4a	YES	No	NO
TCGA-BL-A5ZZ	12.56667	Alive	80	female	26.72287	yes	Papillary	Stage iii	High Grad	MX	N0	T4a	YES	No	NO
TCGA-GV-A3JX	19.36667	Alive	59	male	21.85167	yes	Non-Papill	Stage iii	High Grad	MX	N0	T3b	NO	No	NO
TCGA-GC-A4ZW	0.5	Alive	71	male	26.57589	yes	Non-Papill	Stage iii	High Grad	M0	N0	T3a	NO	No	NA
TCGA-E7-A97P	14.56667	Dead	73	male	17.57813	yes	Non-Papill	Stage ii	High Grad	M0	NX	T2	NO	No	NO
TCGA-C4-A0F7	2.066667	Dead	77	male	23.66144	yes	Non-Papill	Stage iv	High Grad	M0	N2	T4b	NA	No	NA
TCGA-E7-A85H	13.13333	Alive	64	male	17.0068	yes	Non-Papill	Stage iii	High Grad	M0	N0	T3	NO	No	NO
TCGA-UY-A8OB	70.3	Alive	63	male	22.03173	yes	Non-Papill	Stage iv	High Grad	MX	N1	T3a	NA	No	NO
TCGA-HQ-A5NE	12.33333	Dead	57	male	20.2449	yes	Non-Papill	Stage iii	High Grad	M0	N0	T3	NO	No	NO
TCGA-XF-AAMY	100.3667	Alive	78	male	37.55839	yes	Papillary	Stage iii	High Grad	MX	N0	T3b	NO	No	NO
TCGA-XF-AAME	94.26667	Dead	64	female	26.17188	yes	Non-Papill	Stage ii	High Grad	MX	N0	T2b	NO	No	NA
TCGA-FD-A3B6	33.5	Dead	75	male	24.32607	yes	Papillary	Stage ii	High Grad	MX	N0	T2b	NA	No	NO
TCGA-FD-A3B4	17	Dead	55	female	26.5625	yes	Non-Papill	Stage iii	High Grad	MX	N0	T4a	NO	No	NO
TCGA-DK-A6B5	51.4	Alive	45	male	29.32099	yes	Non-Papill	Stage iv	High Grad	M0	N2	T4a	YES	No	NO
TCGA-DK-A2HX	47.33333	Dead	80	female	24.29543	yes	Non-Papill	Stage iv	High Grad	M0	N2	T3	YES	No	NA
TCGA-XF-AAMX	6.866667	Dead	87	female	27.28175	yes	Non-Papill	Stage iii	High Grad	MX	N0	T3b	YES	No	NA
TCGA-XF-AAN5	76.43333	Alive	61	female	19.60716	yes	Non-Papill	Stage iii	High Grad	MX	N0	T3b	YES	No	NO
TCGA-DK-A3IN	8.333333	Dead	72	male	32.27622	yes	Papillary	Stage iii	High Grad	M0	N0	T4a	NA	No	NO
TCGA-K4-A5RH	9.2	Alive	69	male	NA	yes	Non-Papill	Stage iii	High Grad	MX	N0	T3a	YES	No	NO
TCGA-XF-AAN4	27.43333	Dead	77	female	21.67126	yes	Non-Papill	Stage iii	High Grad	MX	N0	T3b	NO	No	NA
TCGA-FD-A3B8	12.8	Alive	56	male	29.26026	yes	Non-Papill	Stage ii	High Grad	MX	N0	T2b	NA	No	NO
TCGA-FD-A43Y	15.8	Dead	65	male	31.95079	yes	Non-Papill	Stage iii	High Grad	MX	N0	T4	NA	No	NO
TCGA-XF-A9SY	21.33333	Dead	60	female	22.67574	yes	Non-Papill	Stage iv	High Grad	MX	N2	T3b	YES	No	NO
TCGA-DK-A1AC	132.7	Alive	72	male	27.43017	yes	Non-Papill	Stage iii	High Grad	M0	N0	T3b	NA	No	NO
TCGA-4Z-AA7N	45.56667	Dead	65	male	30.42185	yes	Non-Papill	Stage iii	High Grad	M0	N0	T3a	YES	No	YES
TCGA-DK-A3WW	21.1	Alive	57	male	24.56931	yes	Papillary	Stage iii	High Grad	M0	N0	T3	NO	No	NO
TCGA-XF-A9T6	2.133333	Alive	88	female	29.21011	yes	Papillary	Stage iii	High Grad	MX	N0	T3b	YES	No	NA
TCGA-XF-A9SP	15.13333	Dead	59	male	24.07547	yes	Non-Papill	Stage iii	High Grad	MX	N0	T3b	YES	No	NA
TCGA-G2-A2EF	62.8	Alive	50	male	24.47587	yes	Non-Papill	Stage ii	High Grad	M0	N0	NA	NA	No	NO
TCGA-FD-A62S	13.53333	Dead	60	female	28.90625	yes	Non-Papill	Stage iii	High Grad	MX	N0	T3b	NA	No	NO
TCGA-E5-A4TZ	15.56667	Dead	64	male	27.76465	yes	Papillary	Stage iv	High Grad	MX	N2	T4b	YES	Yes	NA
TCGA-DK-A6B1	68.3	Alive	67	male	29.63989	yes	Papillary	Stage ii	High Grad	M0	N0	T2a	NO	No	NO
TCGA-ZF-AA4R	34.53333	Dead	67	male	35.34907	yes	Non-Papill	Stage iv	High Grad	MX	N1	T3a	NA	No	NA
TCGA-E7-A5KE	0.566667	Alive	78	female	16.01563	yes	Non-Papill	Stage ii	High Grad	M0	N0	T2a	NO	No	NA
TCGA-XF-AAMH	11.46667	Dead	80	male	34.85018	yes	Non-Papill	Stage iv	High Grad	MX	N1	T3b	YES	No	NA
TCGA-FD-A3SO	5.6	Dead	68	male	25.7122	yes	Non-Papill	Stage iv	High Grad	MX	N1	T3a	NA	No	NO
TCGA-DK-A1A5	2.166667	Dead	79	male	22.03857	yes	Non-Papill	Stage ii	High Grad	M0	N0	T2b	YES	No	NO
TCGA-XF-AAN2	62.3	Dead	73	male	35.83326	yes	Non-Papill	Stage ii	High Grad	MX	N0	T2b	NO	No	NA
TCGA-XF-A9SZ	28.63333	Dead	79	male	26.6436	yes	Non-Papill	Stage iv	High Grad	MX	N2	T3b	YES	No	NA
TCGA-GU-A42Q	11.46667	Dead	67	male	26.00438	yes	Non-Papill	Stage iii	High Grad	M0	N0	T3b	NO	No	NO
TCGA-5N-A9KI	2.533333	Dead	76	female	22.40588	yes	Non-Papill	Stage iii	High Grad	MX	NX	T4	YES	No	NA
TCGA-E7-A6ME	24.36667	Alive	75	male	21.15529	yes	Non-Papill	Stage ii	High Grad	M0	NX	T2b	NO	No	NO
TCGA-4Z-AA86	10.36667	Dead	66	male	NA	yes	Non-Papill	Stage iv	High Grad	M0	N1	T3a	YES	No	NO
TCGA-BT-A0S7	6.666667	Dead	75	male	NA	yes	Non-Papill	Stage iii	High Grad	MX	N0	T4a	NA	No	NO
TCGA-BL-A13J	2.7	Dead	65	male	22.9854	yes	Papillary	Stage iv	High Grad	M0	N2	T4	YES	No	NO
TCGA-FD-A5BY	8.366667	Alive	63	female	23.83673	yes	NA	Stage iii	High Grad	MX	N0	T3a	NA	No	NO
TCGA-GV-A40E	8.7	Dead	75	male	36.29592	yes	Non-Papill	Stage ii	High Grad	MX	NX	NA	NA	No	NO
TCGA-C4-A0EZ	9.1	Dead	69	female	26.60971	yes	Non-Papill	Stage iv	High Grad	M1	N1	T3a	NA	No	NO
TCGA-GU-AATO	10.8	Dead	75	male	20.98765	yes	Papillary	Stage iv	High Grad	MX	N2	T4a	YES	No	NA
TCGA-XF-A9SX	23.96667	Dead	63	female	23.30905	yes	Non-Papill	Stage iv	High Grad	MX	N2	T3b	YES	No	NA
TCGA-4Z-AA7W	28	Alive	55	male	24.31412	yes	Non-Papill	Stage ii	High Grad	M0	N0	T2a	NO	No	NO
TCGA-K4-A5RJ	17.96667	Alive	75	male	NA	yes	Papillary	Stage ii	High Grad	MX	N0	T2b	YES	No	NA
TCGA-2F-A9KT	78.4	Alive	83	male	24.69136	yes	Non-Papill	Stage ii	High Grad	M0	N0	T2b	NO	No	NO
TCGA-FD-A5BT	10.93333	Dead	84	male	22.63468	yes	Non-Papill	Stage iii	High Grad	MX	N0	T3b	NA	No	NA
TCGA-FT-A61P	11.23333	Alive	76	male	30.44983	yes	Papillary	Stage iv	High Grad	MX	N2	T3b	NO	No	NO
TCGA-YC-A9TC	0.666667	Dead	71	male	25.92593	yes	Papillary	Stage iv	High Grad	M1	NA	NA	YES	No	NA
TCGA-ZF-AA58	54.96667	Alive	61	female	25.45807	yes	Non-Papill	Stage iv	High Grad	MX	N2	T3a	NO	No	NO
TCGA-XF-A9SK	16.2	Dead	65	male	26.29657	yes	Non-Papill	Stage iv	High Grad	MX	N1	T3b	YES	No	NA
TCGA-XF-A9SM	34.93333	Alive	76	male	38.51406	yes	Non-Papill	Stage iii	High Grad	MX	N0	T3b	NO	No	NO
TCGA-ZF-A9RD	13.6	Dead	75	female	25.96454	yes	Non-Papill	Stage iv	High Grad	MX	N2	T3a	NO	No	NA
TCGA-XF-A9SW	12.06667	Dead	85	male	20.28651	yes	Non-Papill	Stage iv	High Grad	MX	N2	T3b	YES	No	NA
TCGA-SY-A9G5	39.53333	Alive	66	male	32.14631	yes	Papillary	Stage iii	High Grad	M0	N0	T4a	NO	No	NO
TCGA-G2-AA3C	7.033333	Dead	66	male	26.58318	yes	Non-Papill	Stage iv	High Grad	M0	N1	T3b	YES	No	NA
TCGA-DK-A3IQ	17.96667	Dead	74	male	25.19296	yes	Papillary	Stage iii	High Grad	M0	N0	T3	NA	No	NO
TCGA-XF-A9SJ	3.266667	Dead	83	male	27.75749	yes	Non-Papill	Stage iv	High Grad	MX	N2	T3b	NO	No	NA
TCGA-DK-A3X2	18.23333	Dead	85	male	NA	yes	Papillary	Stage iv	High Grad	M0	N2	T3	YES	No	NO
TCGA-FD-A5BZ	27.83333	Dead	77	female	26.40235	yes	Non-Papill	Stage iv	High Grad	MX	N1	T3a	NA	No	NA
TCGA-2F-A9KR	106.1	Dead	59	female	25.82645	yes	Non-Papill	Stage iii	High Grad	M0	N0	T3a	YES	No	NO
TCGA-UY-A9PB	29.96667	Alive	49	male	41.50932	yes	Non-Papill	Stage iii	High Grad	MX	N0	T3a	NO	No	NO
TCGA-FD-A6TH	4.366667	Dead	63	male	26.12245	yes	Non-Papill	Stage iv	High Grad	MX	N2	T3	YES	Yes	NO

TCGA-XF-AAMW	8.433333	Dead	79	female	25.23634	yes	Non-Papill	Stage iv	High Grad	NA	N0	T2b	NO	No	NA
TCGA-FD-A3SP	26.1	Alive	60	male	28.66613	yes	Non-Papill	Stage iii	High Grad	MX	N0	T3b	YES	No	NO
TCGA-DK-A3WY	165.5667	Alive	67	female	27.88762	yes	Non-Papill	Stage iii	High Grad	M0	N0	T3	NO	No	NO
TCGA-DK-A2I4	127.8333	Alive	79	male	31.74506	yes	Papillary	Stage iii	High Grad	M0	N0	T3b	NA	No	NO
TCGA-XF-AAMG	112.1333	Alive	49	male	23.5102	yes	Non-Papill	Stage iii	High Grad	MX	N0	T4a	NO	No	NO
TCGA-XF-A9ST	4.266667	Dead	68	male	31.87729	yes	Papillary	Stage iii	High Grad	MX	N0	T3b	YES	No	NA
TCGA-FD-A6TF	2.3	Dead	80	female	35.05886	yes	Non-Papill	Stage iv	High Grad	M1	N2	T3b	YES	No	NO
TCGA-FD-A5BX	5.766667	Dead	82	male	33.95918	yes	Non-Papill	Stage iv	High Grad	MX	N1	T3b	YES	No	NA
TCGA-GV-A3QI	37	Alive	47	male	26.6436	yes	Papillary	Stage iii	High Grad	MX	N0	T3b	NO	No	NO
TCGA-XF-AAN8	3.933333	Dead	74	female	22.83737	yes	Non-Papill	Stage iii	High Grad	MX	N0	T3b	NO	No	NA
TCGA-XF-A9T3	2.266667	Alive	69	female	19.53125	yes	Non-Papill	Stage iv	High Grad	MX	N2	T3b	YES	No	NA
TCGA-GD-A3OS	21.26667	Alive	54	female	29.72108	yes	Non-Papill	Stage ii	High Grad	MX	NX	NA	NA	No	NO
TCGA-BT-A20O	12.33333	Dead	75	male	NA	no	Non-Papill	Stage iii	High Grad	MX	N0	T3a	NA	No	NO
TCGA-FJ-A871	9.066667	Dead	49	male	24.89706	yes	Non-Papill	Stage iii	High Grad	MX	NX	T3b	NA	No	NO
TCGA-BL-A0C8	40.63333	Alive	73	male	42.41889	yes	Non-Papill	Stage i	High Grad	M0	NX	T1	NA	No	NO
TCGA-XF-A9SI	80.76667	Alive	73	female	24.9199	yes	Papillary	Stage ii	High Grad	MX	N0	T2b	YES	No	NO
TCGA-ZF-AA54	19.66667	Dead	71	male	28.36035	yes	Non-Papill	Stage iii	High Grad	MX	NX	T3	NO	No	NA
TCGA-BL-A3JM	6.833333	Dead	62	male	22.57687	yes	NA	Stage iii	High Grad	M0	N0	T3	YES	No	NO
TCGA-ZF-AA5N	5.6	Dead	62	female	28.69898	yes	Non-Papill	Stage iv	High Grad	M1	NX	T2	NO	No	NA
TCGA-XF-AAN7	18.83333	Dead	60	male	26.57589	yes	Non-Papill	Stage iv	High Grad	MX	N1	T3a	YES	No	NA
TCGA-FD-A3SR	20.06667	Dead	68	male	30.75637	yes	Non-Papill	Stage iv	High Grad	MX	N2	T4a	YES	No	NO
TCGA-XF-A9T2	19.16667	Dead	54	male	33.16795	yes	Non-Papill	Stage iii	High Grad	MX	N0	T3b	YES	No	NA
TCGA-BL-A13I	7.433333	Dead	57	female	20.69049	yes	Non-Papill	Stage iii	High Grad	M0	N0	T3	NA	No	NO
TCGA-XF-AAN8	3.933333	Dead	74	female	22.83737	yes	Non-Papill	Stage iii	High Grad	MX	N0	T3b	NO	No	NA

Basic information of IMvigor210 cohort

ID	survival_ti	status	Response	IC_level	TC_level	Immune_phenotype
SAMf275eb859a39	24.47639	0	CR/PR	IC1	TC0	excluded
SAMc1b27bc16435	24.1807	0	CR/PR	IC2	TC0	excluded
SAM36a9225b0222	23.8193	0	SD/PD	IC2	TC2+	inflamed
SAM36d87392593b	23.68789	0	SD/PD	IC2	TC2+	excluded
SAMd027124354ce	23.52361	0	CR/PR	IC0	TC0	desert
SAM681e4bf7cf85	23.3922	0	CR/PR	IC2	TC0	excluded
SAMb0a83e5fbde9	23.32649	0	SD/PD	IC0	TC0	NA
SAM0684af734db1	23.26078	0	CR/PR	IC0	TC0	NA
SAM6cb230f208a8	23.22793	0	CR/PR	IC0	TC1	desert
SAM8884fe446d20	23.16222	0	CR/PR	IC2	TC2+	inflamed
SAMd3601288319e	23.12936	0	CR/PR	IC2	TC0	excluded
SAM7fb6987514a4	22.83368	0	SD/PD	IC2	TC0	excluded
SAM1fa6bcb7fc48	22.73511	0	SD/PD	IC2	TC0	inflamed
SAMb419a8fcbfcd	22.73511	0	CR/PR	IC2	TC0	excluded
SAMfb7aec7cb0e2	22.53799	0	CR/PR	IC2	TC2+	inflamed
SAM62fb1388c871	22.50513	0	SD/PD	IC1	TC0	NA
SAMb3c02294aba7	22.40657	0	CR/PR	IC0	TC1	desert
SAM2570ff4aae6e	22.37372	0	CR/PR	IC2	TC0	inflamed
SAM2e9ac0b1b250	22.14374	0	CR/PR	IC2	TC2+	NA
SAM08cce2fa88f2	22.11088	0	SD/PD	IC0	TC0	excluded
SAM6d2ae0c39b96	22.11088	0	SD/PD	IC1	TC0	excluded
SAMe712352fb82a	22.11088	0	CR/PR	IC0	TC0	desert
SAM670649e105b5	22.07803	0	CR/PR	IC2	TC0	excluded
SAMa9ca8536d2b1	21.84805	0	SD/PD	IC1	TC1	NA
SAM7b40007f4aa4	21.65092	0	CR/PR	IC0	TC0	NA
SAMc1251c7bfee2	21.61807	0	CR/PR	IC2	TC0	excluded
SAM28687037e4ff	21.58522	0	SD/PD	IC2	TC2+	inflamed
SAMd35318127278	21.58522	0	CR/PR	IC1	TC1	excluded
SAMeaa477a5384b	21.58522	0	CR/PR	IC2	TC0	inflamed
SAM3b15b4c6311d	21.4538	0	CR/PR	IC2	TC2+	inflamed
SAM14938611a2d3	21.42094	0	CR/PR	IC1	TC0	desert
SAM14df63a65411	21.42094	0	CR/PR	IC2	TC2+	excluded
SAM560f23d6a3ad	21.38809	0	CR/PR	IC2	TC2+	inflamed
SAM61b9d4d84c64	21.38809	0	SD/PD	IC2	TC0	inflamed
SAM075e037d95bc	21.32238	0	CR/PR	IC1	TC0	excluded
SAM1dda30f1c5be	21.25667	0	SD/PD	IC1	TC0	desert
SAM73663ee4a96e	21.25667	0	CR/PR	IC2	TC0	excluded
SAM7d7c54623618	21.22382	0	CR/PR	IC0	TC0	excluded
SAM716f54e468f4	21.19097	0	SD/PD	IC2	TC0	NA
SAMae1690469964	21.15811	1	SD/PD	IC1	TC0	NA
SAMd697ba701077	21.15811	1	SD/PD	IC0	TC0	excluded
SAMe1eb5d988760	21.05955	0	CR/PR	IC2	TC0	inflamed
SAMb8070b7937e7	21.02669	1	SD/PD	IC1	TC0	desert
SAMaec7380f9ab0	20.86242	0	CR/PR	IC2	TC0	excluded
SAMd3bd67996035	20.82957	0	CR/PR	IC2	TC0	excluded
SAM1c8b086175ca	20.76386	1	SD/PD	IC0	TC0	excluded
SAM8f2275c36e8c	20.73101	0	SD/PD	IC1	TC0	desert
SAMbf1a3ae828e6	20.73101	0	CR/PR	IC2	TC0	excluded
SAMc2a1820d4e6b	20.73101	0	CR/PR	IC2	TC0	excluded
SAMc919aebc7fdd	20.73101	0	CR/PR	IC2	TC0	inflamed
SAM25510f300d79	20.69815	0	CR/PR	IC0	TC0	desert
SAMeb587a68006b	20.69815	0	SD/PD	IC1	TC0	NA
SAM6083aac8db99	20.63244	0	CR/PR	IC1	TC0	inflamed
SAMbda79f955628	20.63244	0	SD/PD	IC2	TC0	inflamed
SAM8a1b0e02ee42	20.56674	0	CR/PR	IC0	TC0	desert
SAMaaf505c36f93	20.56674	0	CR/PR	IC1	TC0	excluded
SAMfed609955db9	20.56674	0	SD/PD	IC2	TC2+	desert

SAM2eb07dedf07f	20.50103	0	CR/PR	IC2	TC0	inflamed
SAM52500cabdd36	20.46817	0	SD/PD	IC1	TC0	excluded
SAMcabb6d58ff55	20.27105	0	SD/PD	IC2	TC2+	inflamed
SAMd7d57ee3a863	20.27105	0	CR/PR	IC2	TC0	inflamed
SAM065890737112	20.07392	0	CR/PR	IC2	TC0	inflamed
SAMb470eb8f04be	20.07392	0	CR/PR	IC2	TC0	excluded
SAM4581bac493af	20.04107	0	NA	IC2	TC0	NA
SAMb15ac6e4c4ef	20.00821	0	CR/PR	IC1	TC0	NA
SAMd43f8933066b	20.00821	0	CR/PR	IC1	TC0	inflamed
SAM771445e92421	19.41684	0	CR/PR	IC1	TC0	inflamed
SAM203dcf14f927	19.35113	0	CR/PR	IC1	TC1	inflamed
SAM6662f5181f87	19.28542	0	SD/PD	IC1	TC0	inflamed
SAM6dd7ad1d797d	19.28542	1	SD/PD	IC1	TC2+	NA
SAM6f2a102a99df	19.28542	0	CR/PR	IC2	TC2+	NA
SAM3330c03fdf00	19.18686	0	SD/PD	IC1	TC0	NA
SAM7538ad9ff524	19.12115	0	SD/PD	IC1	TC0	inflamed
SAM9fb814c22bdb	19.12115	1	SD/PD	IC1	TC0	desert
SAMe50d15fde368	18.89117	0	CR/PR	IC1	TC0	desert
SAM27299aed7681	18.85832	0	CR/PR	IC1	TC0	excluded
SAM47fc46c3d6be	18.7269	0	SD/PD	IC2	TC0	inflamed
SAM727c0e92a2a7	18.7269	0	CR/PR	IC2	TC2+	NA
SAMe9475f77504b	18.66119	0	SD/PD	IC1	TC0	excluded
SAM1bcc62d8290c	18.62834	0	NA	IC1	TC0	excluded
SAM6ff654a20f98	18.56263	0	CR/PR	IC1	TC0	excluded
SAM34430ef08e5b	18.16838	0	SD/PD	IC1	NA	NA
SAM5767dd75d142	18.16838	0	CR/PR	IC1	TC0	excluded
SAMd5ab7fbfab4e	18.10267	0	NA	IC2	TC2+	excluded
SAM7edacb3deb65	18.00411	0	NA	IC0	TC0	desert
SAM415f36ad349e	17.97125	1	SD/PD	IC1	TC0	excluded
SAM39eb94fa504d	17.90554	1	SD/PD	IC2	TC0	excluded
SAM59f392864f5d	17.77413	1	SD/PD	IC2	TC0	excluded
SAM3e04eb914f3d	17.28131	0	CR/PR	IC2	TC0	excluded
SAM548551ef782c	17.28131	0	CR/PR	IC0	TC0	desert
SAM822b226466a1	17.28131	0	SD/PD	IC0	TC0	desert
SAMa535fcdf18a0	17.28131	0	CR/PR	IC0	TC0	NA
SAMcc7a42d87e9c	17.21561	0	CR/PR	IC2	TC0	inflamed
SAM31d9176e11fb	17.11704	0	CR/PR	IC2	TC1	inflamed
SAM5fe7a81a39dd	17.08419	1	SD/PD	IC2	TC0	inflamed
SAM91c47b054ffb	17.01848	1	CR/PR	IC2	TC0	inflamed
SAM1abf01dd4544	16.85421	0	CR/PR	IC1	TC0	excluded
SAM572f19794c96	16.82136	0	CR/PR	IC1	TC2+	excluded
SAM1f66db567eb5	16.59138	0	CR/PR	IC2	TC0	inflamed
SAM6157c8f38b72	16.59138	0	CR/PR	IC1	TC0	desert
SAM18a4dabbc557	16.45996	1	SD/PD	IC2	TC2+	excluded
SAM58e7832f4e7d	16.45996	0	CR/PR	IC0	TC0	desert
SAM9daccafc18db	16.45996	0	SD/PD	IC0	TC0	desert
SAM6964a6d7b967	16.26283	1	SD/PD	IC1	TC1	NA
SAMcf018fee2acd	16.22998	1	SD/PD	IC0	TC0	excluded
SAM3a1c9632ff7b	16.03285	0	SD/PD	IC0	TC0	NA
SAM18b9351e265a	15.86858	1	SD/PD	IC2	TC0	inflamed
SAM5cfa1699bdb7	15.83573	0	SD/PD	IC0	TC0	NA
SAMdb3f50c9129c	15.80287	1	SD/PD	IC0	TC0	excluded
SAM4caabd64e7fd	15.70431	0	CR/PR	IC1	TC0	excluded
SAM8b4b8b0f9e73	15.67146	0	SD/PD	IC1	TC0	excluded
SAM0257bbbbd388	15.6386	1	SD/PD	IC1	TC0	excluded
SAM3b1066e5801b	15.6386	0	SD/PD	IC2	TC0	excluded
SAM61baf919bb01	15.60575	1	SD/PD	IC0	TC0	desert
SAMffa5c7cad0e5	15.54004	1	CR/PR	IC1	TC0	desert
SAM9306c5c92444	15.44148	0	SD/PD	IC2	TC1	inflamed

SAM9e11ec6bea80	15.40862	1	SD/PD	IC2	TC2+	inflamed
SAMe94c30c30616	15.37577	1	SD/PD	IC1	TC0	excluded
SAM2b672f4336c7	15.31006	1	SD/PD	IC0	TC0	desert
SAMad83c9c53537	14.98152	0	SD/PD	IC1	TC0	NA
SAM75f12d1a55fc	14.75154	0	SD/PD	IC2	TC0	inflamed
SAM80c6183220e6	14.75154	0	SD/PD	IC1	TC0	inflamed
SAMbc8dc3a7b54e	14.75154	1	SD/PD	IC0	TC0	desert
SAM76a431ba6ce1	14.12731	1	SD/PD	IC1	TC0	inflamed
SAMda4d892fddc8	14.12731	0	SD/PD	IC1	TC0	NA
SAMe3d4266775a9	13.99589	0	SD/PD	IC0	TC0	NA
SAMb8101c538753	13.40452	1	SD/PD	IC2	TC2+	inflamed
SAM94859b440b1d	13.33881	1	SD/PD	IC1	TC0	excluded
SAMab8052a03398	13.30595	1	SD/PD	IC2	TC0	inflamed
SAM4b7ea015fd9e	13.2731	1	SD/PD	IC1	TC0	excluded
SAMc0d625a50eb8	13.2731	1	SD/PD	IC0	TC0	desert
SAM07a93a28f801	12.846	0	CR/PR	IC1	TC0	excluded
SAM568ce160abd9	12.846	1	SD/PD	IC0	TC0	NA
SAMba1a34b5a060	12.846	1	SD/PD	IC2	TC0	inflamed
SAM8533e5e261d6	12.81314	1	CR/PR	IC0	TC0	desert
SAM7d2dfba6cd84	12.71458	1	SD/PD	IC1	TC0	inflamed
SAM97a00e0929fb	12.71458	0	CR/PR	IC2	TC0	excluded
SAM1e9c4d1d39ae	12.41889	1	SD/PD	IC1	TC0	inflamed
SAM75142fcab9df	11.92608	1	SD/PD	IC2	TC0	excluded
SAM30cf07d4874f	11.66324	1	SD/PD	IC1	TC0	excluded
SAMb2f1d0e54ece	11.40041	1	CR/PR	IC2	TC2+	desert
SAM753d4bb52dbe	11.36756	1	SD/PD	IC1	TC0	excluded
SAM2c9586161ce6	11.26899	1	SD/PD	IC1	TC0	desert
SAMe97af0feefdf	11.10472	1	SD/PD	IC1	TC0	excluded
SAM5d1dfd5207f5	10.9076	1	SD/PD	IC0	TC0	NA
SAM181b638b8248	10.87474	1	SD/PD	IC2	TC2+	excluded
SAM7114d99032ec	10.84189	1	SD/PD	IC0	TC0	NA
SAMa1e62d323e1d	10.57906	1	SD/PD	IC2	TC0	inflamed
SAMb0d11db9aa79	10.5462	1	SD/PD	IC2	TC0	inflamed
SAM2bba8cb35e48	10.48049	1	SD/PD	IC0	TC0	excluded
SAMd636e3461955	10.48049	1	SD/PD	IC1	TC0	inflamed
SAM0f956e757453	10.41478	1	SD/PD	IC1	TC0	excluded
SAMf2aae1443f67	10.34908	1	SD/PD	IC1	TC0	desert
SAM7bff231634e9	10.25051	1	SD/PD	IC2	TC2+	NA
SAM2dc3f04e45e9	10.1848	1	SD/PD	IC2	TC2+	inflamed
SAMef0e3d2415fd	10.1191	1	SD/PD	IC2	TC2+	inflamed
SAM19fec8f3b3bd	9.889117	1	SD/PD	IC2	TC2+	excluded
SAM49d48750e294	9.856263	1	SD/PD	IC1	TC0	excluded
SAMe56c96c51190	9.7577	1	SD/PD	IC1	TC2+	excluded
SAMc0ef41aa6c8b	9.560575	1	SD/PD	IC0	TC0	desert
SAMc0da5d48686d	9.494867	1	SD/PD	IC1	TC2+	desert
SAM30b5c6c54cf7	9.264887	1	CR/PR	IC2	TC0	excluded
SAM28e6031ac18b	9.232033	1	SD/PD	IC0	TC0	desert
SAMfd947610629d	9.034908	1	SD/PD	IC0	TC0	desert
SAM18be5b395318	9.002053	1	SD/PD	IC2	TC1	excluded
SAM9725303dce0c	8.903491	0	SD/PD	IC1	TC0	desert
SAM9aa6a095a9d6	8.837782	1	SD/PD	IC1	TC0	excluded
SAM025b45c27e05	8.772074	1	SD/PD	IC1	TC0	desert
SAM3ee5dcd894f0	8.706366	1	SD/PD	IC0	TC0	desert
SAM31f41dd0d6ca	8.279261	1	SD/PD	IC1	TC0	NA
SAM7c67b05aa109	8.246407	1	SD/PD	IC0	TC1	NA
SAM297c0301e861	8.082136	1	SD/PD	IC1	TC2+	excluded
SAM65afda25b920	8.082136	1	SD/PD	IC2	TC2+	excluded
SAMeff2ce356ccb	8.082136	1	SD/PD	IC0	TC0	desert
SAM2070b416069c	8.016427	1	SD/PD	IC1	TC1	excluded

SAMc57eadb2d82b	8.016427	1	SD/PD	IC0	TC0	desert
SAM52e3fa3ad574	7.950719	1	SD/PD	IC1	TC0	NA
SAM9539a4f19ebc	7.950719	1	SD/PD	IC0	TC0	desert
SAMe7bcab05402e	7.917864	1	SD/PD	IC0	TC0	NA
SAM2f228939632f	7.88501	1	SD/PD	IC1	TC0	excluded
SAMd2492b2a31bb	7.852156	1	SD/PD	IC2	TC2+	desert
SAM2e7aa8fa0ab3	7.720739	1	SD/PD	IC1	TC0	excluded
SAM9cafb905b36a	7.622177	1	SD/PD	IC2	TC2+	inflamed
SAM49f9b2e57aa5	7.457906	1	SD/PD	IC0	TC0	excluded
SAM12502d970c10	7.392197	1	SD/PD	IC2	TC0	inflamed
SAMdf3e42c8672a	7.326489	1	SD/PD	IC2	TC2+	inflamed
SAM5c139c5c1c4f	7.227926	1	SD/PD	IC2	TC0	excluded
SAM3785587846ce	7.063655	1	NA	IC1	TC0	NA
SAMa321770ac31c	7.063655	0	SD/PD	IC2	TC0	excluded
SAM3e8baff50d7a	6.965092	1	SD/PD	IC0	TC0	desert
SAM18bc1078bc15	6.899384	1	SD/PD	IC1	TC0	NA
SAM63405b04ab2d	6.800821	1	SD/PD	IC0	TC0	NA
SAM5e3bae090b8c	6.735113	1	SD/PD	IC2	TC0	excluded
SAM9410b866974a	6.735113	1	SD/PD	IC0	TC0	NA
SAM553c3c35b847	6.702259	1	SD/PD	IC0	TC0	NA
SAM557dde1b9f3e	6.702259	1	SD/PD	IC1	TC0	excluded
SAMbfdffb97c446	6.702259	1	SD/PD	IC1	TC2+	excluded
SAM0ce9c983b20f	6.603696	1	SD/PD	IC2	TC0	excluded
SAM09c84ec0cf34	6.505133	1	SD/PD	IC0	TC0	inflamed
SAMf28c01545593	6.406571	1	SD/PD	IC1	TC0	desert
SAMce39dd79b441	6.275154	1	SD/PD	IC2	TC0	inflamed
SAM6780ed436b55	6.2423	1	SD/PD	IC2	TC0	inflamed
SAMdcae54fcd7fa	6.2423	1	SD/PD	IC1	TC0	excluded
SAMe5bc41772bc9	6.110883	1	SD/PD	IC1	TC0	excluded
SAM6792d6e98068	6.01232	1	SD/PD	IC2	TC0	desert
SAM6cbc10abddb0	5.946612	1	SD/PD	IC2	TC0	inflamed
SAM5234688806a7	5.880903	1	SD/PD	IC0	TC0	desert
SAM961d04c42bd9	5.880903	1	SD/PD	IC0	TC0	excluded
SAM1c0ecfb3eb63	5.848049	1	SD/PD	IC2	TC0	excluded
SAM85f0a3ac1c45	5.848049	1	SD/PD	IC2	TC0	excluded
SAM23aa15d4a0b0	5.716632	1	SD/PD	IC1	TC0	excluded
SAM5cc2d9036053	5.683778	1	SD/PD	IC0	TC0	desert
SAM9a2cf3c06fb3	5.650924	1	SD/PD	IC1	TC0	inflamed
SAMc692536a795a	5.650924	1	SD/PD	IC1	TC0	desert
SAM1ac4e3dee297	5.519507	1	SD/PD	IC1	TC0	excluded
SAMaf42c1541269	5.519507	0	SD/PD	IC2	TC0	inflamed
SAMe9ae8beb82fa	5.486653	1	SD/PD	IC1	TC0	desert
SAM166a419a4e5a	5.38809	1	SD/PD	IC1	TC0	excluded
SAM1a87df750b9d	5.38809	1	SD/PD	IC1	TC0	excluded
SAM26104d5adc89	5.38809	1	SD/PD	IC0	TC0	excluded
SAMd4c0837b0997	5.38809	1	SD/PD	IC2	TC1	excluded
SAMa913c6139ec8	5.125257	1	SD/PD	IC2	TC0	excluded
SAM3cb94b0d5297	5.059548	1	SD/PD	IC1	TC0	excluded
SAMae4da274eded	4.99384	1	SD/PD	IC0	TC0	NA
SAM9681450bbc90	4.960986	1	NA	IC2	TC0	inflamed
SAM5a2347c0498a	4.895277	1	SD/PD	IC0	TC0	desert
SAM7f0d9cc7f001	4.632444	1	SD/PD	IC1	TC0	excluded
SAM0571f17f4045	4.533881	1	SD/PD	IC2	TC0	excluded
SAM8e8ef2368dfa	4.501027	1	SD/PD	IC0	TC0	desert
SAM54e58f1b0230	4.36961	1	SD/PD	IC0	TC0	desert
SAMb2e4a082541a	4.36961	1	SD/PD	IC1	TC0	NA
SAMf20b827dca51	4.36961	1	SD/PD	IC2	TC0	inflamed
SAM110501d0eedb	4.271047	1	SD/PD	IC1	TC0	desert
SAMbcbc7957c264	4.172485	1	SD/PD	IC1	TC0	desert

SAMe0c49ea0df5d	4.13963	1	SD/PD	IC1	TC0	excluded
SAM63b2189c36d7	3.975359	1	SD/PD	IC0	TC0	desert
SAMdee1011782cd	3.909651	1	SD/PD	IC1	TC0	NA
SAM468a9e1dc821	3.876797	1	SD/PD	IC2	TC0	desert
SAM7829a341b9f3	3.876797	1	SD/PD	IC2	TC1	inflamed
SAMb15ad09d6e24	3.876797	1	SD/PD	IC2	TC2+	excluded
SAM5b57e47fdcb3	3.843943	1	SD/PD	IC2	TC0	NA
SAM59fda9035d1d	3.74538	1	SD/PD	IC0	TC0	NA
SAM8e469834acc1	3.712526	1	SD/PD	IC1	TC0	excluded
SAM99a46b9eec27	3.613963	1	SD/PD	IC0	TC0	excluded
SAMd135d5867fe3	3.613963	1	SD/PD	IC2	TC2+	excluded
SAM36851bc8b9ae	3.548255	1	SD/PD	IC1	TC2+	excluded
SAMa424c75831b4	3.548255	1	SD/PD	IC2	TC0	NA
SAM1f83ebd6be9b	3.5154	1	SD/PD	IC1	TC0	desert
SAM45c8e6412c66	3.482546	1	SD/PD	IC1	TC2+	inflamed
SAM5d989c86255e	3.449692	1	SD/PD	IC1	TC0	desert
SAMba7176afe070	3.449692	0	SD/PD	IC0	TC0	excluded
SAMae02629a97f7	3.252567	1	SD/PD	IC0	TC0	NA
SAMee3844cc0b9f	3.219713	1	SD/PD	IC0	TC0	NA
SAMb4c7a001537d	3.186858	1	SD/PD	IC1	TC1	inflamed
SAMcc4675f394a1	3.12115	1	SD/PD	IC0	TC0	NA
SAMe7bf6c015192	3.12115	1	SD/PD	IC2	TC0	inflamed
SAMeb29625f76a5	3.12115	1	SD/PD	IC1	TC0	excluded
SAMf82bbdc267c8	3.12115	1	SD/PD	IC0	TC0	desert
SAMbe25e2c88f3e	2.924025	1	NA	IC2	TC1	NA
SAM675a12a09c15	2.89117	1	SD/PD	IC1	TC0	desert
SAM9b9d48b0b02c	2.89117	1	NA	IC1	TC0	inflamed
SAM0d855cff64e6	2.858316	1	SD/PD	IC1	TC0	desert
SAM7746b76437e6	2.825462	1	NA	IC0	TC0	desert
SAM8a42c0d59187	2.825462	1	NA	IC0	TC0	NA
SAM87a8e18eb45b	2.759754	1	SD/PD	IC1	TC0	excluded
SAMb963dda93cfd	2.759754	1	SD/PD	IC2	TC2+	excluded
SAMbe83eae4026e	2.726899	1	SD/PD	IC1	TC0	excluded
SAM17c45bf16bb6	2.694045	1	NA	IC0	TC0	excluded
SAMb8f13a0525a6	2.694045	1	NA	IC2	TC0	excluded
SAM7893196e0e89	2.661191	1	SD/PD	IC0	TC0	NA
SAM0a7c2091dd56	2.628337	1	SD/PD	IC2	TC1	excluded
SAMaabf4afe4213	2.628337	1	SD/PD	IC0	TC0	NA
SAM29da928587ad	2.595483	1	SD/PD	IC1	TC0	desert
SAM2624229effe8	2.562628	1	SD/PD	IC0	TC0	excluded
SAMbcb07ba81cee	2.529774	1	SD/PD	IC2	TC0	excluded
SAMcb132b0cdd2c	2.529774	1	SD/PD	IC1	TC2+	desert
SAM032c642382a7	2.49692	1	SD/PD	IC2	TC2+	inflamed
SAMff41c4e8c08f	2.464066	1	NA	IC0	TC0	excluded
SAMdad5c29dc105	2.431211	1	SD/PD	IC1	TC0	excluded
SAM187e056d6a2a	2.299795	1	SD/PD	IC0	TC0	NA
SAM0bdb3428bd13	2.234086	1	SD/PD	IC0	TC0	NA
SAM23095936e611	2.234086	1	SD/PD	IC2	TC0	desert
SAM957378bd907f	2.234086	1	SD/PD	IC0	TC0	excluded
SAMd86389d0d768	2.201232	1	SD/PD	IC0	TC0	desert
SAMe3210d3632b4	2.168378	1	SD/PD	IC2	TC2+	inflamed
SAM563d6233dfa2	2.135524	1	SD/PD	IC0	TC0	NA
SAM7aa01fc49a80	2.135524	1	SD/PD	IC1	TC0	excluded
SAM81b71522417a	2.135524	1	SD/PD	IC0	TC0	NA
SAM943df5cf15df	2.135524	1	SD/PD	IC1	TC0	desert
SAMaff272833538	2.135524	1	NA	IC2	TC0	excluded
SAM1ab1b28d9f2b	2.102669	1	SD/PD	IC1	TC0	desert
SAM3894ac3956a5	2.102669	0	SD/PD	IC1	TC0	excluded
SAM978a587b207e	2.102669	1	SD/PD	IC0	TC2+	excluded

SAMe07c4560772d	2.102669	0	SD/PD	IC0	TC0	desert
SAMf3a9bce50099	2.102669	0	SD/PD	IC2	TC0	excluded
SAM4b0175e8db6e	2.069815	1	SD/PD	IC1	TC0	desert
SAMd98bac0a070f	2.069815	1	SD/PD	IC0	TC0	desert
SAM9448d858692c	2.036961	1	NA	IC2	TC1	inflamed
SAM2dc578e0165f	1.971253	1	NA	IC2	TC2+	excluded
SAMe7e4f7c076a7	1.971253	1	SD/PD	IC2	TC2+	inflamed
SAMd1bd63734394	1.938398	1	SD/PD	IC1	TC0	NA
SAMfddc359e862b	1.938398	1	NA	IC0	TC0	excluded
SAM00b9e5c52da9	1.905544	1	NA	IC1	TC0	excluded
SAM491e341d5a82	1.87269	1	NA	IC1	TC0	desert
SAM2de7cffb5f72	1.839836	1	NA	IC1	TC2+	NA
SAM73b653ae20d1	1.806982	1	NA	IC2	TC2+	inflamed
SAM7a9093b9c7e9	1.806982	1	NA	IC0	TC0	desert
SAMc6eff056c89a	1.806982	1	SD/PD	IC0	TC0	excluded
SAMa1871f491b02	1.741273	1	SD/PD	IC0	TC0	desert
SAM59289ca42c99	1.708419	1	NA	IC1	TC0	excluded
SAM7ee2b6e4d6b3	1.708419	1	NA	IC0	TC0	excluded
SAMd215b503f99a	1.708419	0	SD/PD	IC0	TC0	NA
SAM7fb7a13c096b	1.675565	1	SD/PD	IC0	TC0	excluded
SAMcee0fa8c05b4	1.675565	1	NA	IC1	TC1	excluded
SAMf2ce197162ce	1.577002	1	NA	IC1	TC1	NA
SAM18039827e1b9	1.478439	1	SD/PD	IC0	TC0	desert
SAM1f3c93814cb9	1.445585	1	NA	IC0	TC0	excluded
SAM9d2494119c05	1.445585	1	SD/PD	IC0	TC0	NA
SAM4305ab968b90	1.412731	1	SD/PD	IC0	TC0	desert
SAM3779e979db6b	1.314168	1	NA	IC1	TC0	excluded
SAM5ffd7e4cd794	1.215606	1	NA	IC0	TC0	NA
SAM0a0f2bac4b20	1.182752	1	SD/PD	IC1	TC1	NA
SAM31291c256373	1.149897	1	NA	IC1	TC0	inflamed
SAM4501e41e4751	1.117043	1	SD/PD	IC1	TC1	excluded
SAM4edbe45817b3	1.051335	1	NA	IC2	TC2+	inflamed
SAMa90d73f8d891	1.01848	1	SD/PD	IC0	TC0	NA
SAMd0e47be700b0	0.887064	1	NA	IC0	TC0	desert
SAMbd8ee73983b8	0.854209	1	NA	IC1	TC0	excluded
SAMe41b1e773582	0.854209	1	SD/PD	IC1	TC0	excluded
SAM3f2033c90438	0.821355	1	NA	IC0	TC0	inflamed
SAM9eebdef2858a	0.821355	1	NA	IC1	TC0	excluded
SAMc97f35a29d16	0.821355	0	NA	IC0	TC0	desert
SAM3f446449bf81	0.755647	1	NA	IC2	TC0	inflamed
SAM04c589eb3fb3	0.689938	0	NA	IC1	TC0	NA
SAM4918c524b83a	0.62423	0	NA	IC1	TC0	desert
SAM5fc9ae0aed1f	0.62423	1	NA	IC2	TC2+	inflamed
SAM714285adf612	0.62423	1	NA	IC2	TC2+	inflamed
SAM85e41e7f33f9	0.62423	1	NA	IC1	TC1	inflamed
SAMbf91f27e7f9b	0.591376	1	NA	IC2	TC0	excluded
SAMabc151b01ea3	0.558522	1	SD/PD	IC1	TC0	excluded
SAM59b825252c0d	0.525667	1	NA	IC1	TC0	NA
SAM8e43e9caf307	0.492813	1	NA	IC1	TC0	excluded
SAM99b1f6a9534e	0.492813	1	NA	IC1	TC0	excluded
SAM698d8d76b934	0.427105	1	NA	IC1	TC0	excluded
SAM95c70496ffb5	0.361396	1	NA	IC2	TC0	inflamed
SAMa0ca029b7afd	0.361396	1	NA	IC0	TC0	NA
SAMaf7578d55754	0.229979	1	NA	IC0	TC2+	excluded
SAMdab9ca8fb5de	0.197125	0	SD/PD	IC0	TC0	excluded

Basic information of GSE13507 cohort

ID	status	survival_ti	TMN stag	Grade
GSM340605	1	18.17	T1N0M0	1
GSM340606	0	136.97	TaN0M0	1
GSM340607	0	136.37	TaN0M0	1
GSM340608	1	26.90	T1N0M0	1
GSM340609	1	87.07	T1N0M0	1
GSM340610	1	15.30	T1N0M0	1
GSM340611	1	18.53	T1N0M0	1
GSM340612	0	129.93	T2N0M0	1
GSM340613	1	12.57	T3bN0M0	2
GSM340614	0	128.97	T3aN0M0	1
GSM340615	0	125.03	TaN0M0	1
GSM340616	0	125.27	TaN0M0	1
GSM340617	0	130.60	T1N0M0	1
GSM340618	1	1.03	T4N0M0	1
GSM340619	0	129.67	T1N0M0	1
GSM340620	1	16.67	T3aN2M1	1
GSM340621	0	127.63	T1N0M0	1
GSM340622	0	124.53	TaN0M0	1
GSM340623	1	3.13	T4N2M1	2
GSM340624	1	65.93	T1N0M0	1
GSM340625	1	3.23	T1N0M0	1
GSM340626	0	120.60	T1N0M0	1
GSM340627	1	22.67	T1N0M0	1
GSM340628	0	120.50	T3aN0M0	2
GSM340629	1	50.43	T1N0M0	1
GSM340630	1	66.30	T2N0M0	1
GSM340631	0	120.17	TaN0M0	1
GSM340632	0	120.60	T1N0M0	1
GSM340633	1	14.57	T3bN0M0	2
GSM340634	0	115.83	T3aN0M0	2
GSM340635	0	106.60	TaN0M0	1
GSM340636	0	121.20	T2N0M0	2
GSM340637	1	98.00	T1N0M0	1
GSM340638	1	67.03	T1N0M0	1
GSM340639	0	99.73	TaN0M0	1
GSM340640	1	50.30	TaN0M0	1
GSM340641	0	98.23	T1N0M0	2
GSM340642	0	106.03	T1N0M0	1
GSM340643	0	95.30	T1N0M0	1
GSM340644	1	80.43	TaN0M0	1
GSM340645	1	35.70	T1N0M0	2
GSM340646	1	51.77	T1N0M0	2
GSM340647	0	92.83	T1N0M0	1
GSM340648	0	90.73	T2N0M0	1
GSM340649	0	90.47	T1N0M0	1
GSM340650	0	86.07	T1N0M0	1
GSM340651	1	134.97	T1N0M0	1
GSM340652	1	65.93	T1N0M0	1
GSM340653	1	25.83	T3bN0M0	2
GSM340654	1	10.27	T3bN2M0	2
GSM340655	1	85.43	T1N0M0	2
GSM340656	1	3.17	T1N0M0	2
GSM340657	0	83.63	T1N0M0	1
GSM340658	0	83.63	T1N0M0	1
GSM340659	1	70.93	T1N0M0	1
GSM340660	0	81.63	T2N0M0	1
GSM340661	0	80.20	T1N0M0	1

GSM340662	0	78.83	T1N0M0	2
GSM340663	0	78.73	T1N0M0	2
GSM340664	1	70.73	TaN0M0	1
GSM340665	1	8.70	T2N1M1	2
GSM340666	0	75.33	T1N0M0	1
GSM340667	0	75.70	T1N0M0	1
GSM340668	0	75.13	TaN0M0	1
GSM340669	0	75.13	T1N0M0	1
GSM340670	0	74.17	TaN0M0	1
GSM340671	1	16.23	T1N0M0	2
GSM340672	1	15.10	T3bN0M0	2
GSM340673	1	11.23	T2N0M0	1
GSM340674	0	69.43	T2N0M0	2
GSM340675	1	25.03	T1N0M0	2
GSM340676	0	67.07	TaN0M0	1
GSM340677	0	65.23	T2N0M0	2
GSM340678	1	11.97	T4N0M0	2
GSM340679	0	62.00	T1N0M0	1
GSM340680	0	61.33	T1N0M0	1
GSM340681	0	60.90	T1N0M0	2
GSM340682	0	60.20	T1N0M0	1
GSM340683	0	59.60	T1N0M0	1
GSM340684	1	10.40	T4N0M0	2
GSM340685	1	59.87	T1N0M0	1
GSM340686	0	59.00	T1N0M0	1
GSM340687	0	58.73	T1N0M0	1
GSM340688	1	46.17	T1N0M0	1
GSM340689	0	58.43	T1N0M0	1
GSM340690	0	58.07	T1N0M0	1
GSM340691	0	57.63	T1N0M0	1
GSM340692	1	36.30	T1N0M0	1
GSM340693	0	56.47	T2N0M0	2
GSM340694	0	55.50	T2N0M0	2
GSM340695	0	52.97	T1N0M0	1
GSM340696	1	11.50	T3N1M0	2
GSM340697	1	17.87	T3N0M0	2
GSM340698	1	28.53	T1N0M0	2
GSM340699	1	17.63	T1N0M0	1
GSM340700	1	15.40	T2N0M0	2
GSM340701	0	48.43	T3bN0M0	2
GSM340702	1	4.50	T4N1M1	1
GSM340703	0	47.50	TaN0M0	1
GSM340704	1	5.23	T2N1M0	2
GSM340705	0	45.60	T3bN0M0	2
GSM340706	0	45.13	T2N0M0	2
GSM340707	0	43.50	TaN0M0	1
GSM340708	0	44.10	T1N0M0	2
GSM340709	1	5.93	T2N0M0	1
GSM340710	1	31.53	T1N0M0	2
GSM340711	0	41.80	T1N0M0	1
GSM340712	1	6.50	T2N2M0	2
GSM340713	0	41.07	T1N0M0	1
GSM340714	0	39.77	T2bN0M0	2
GSM340715	0	38.97	T2N0M0	1
GSM340716	1	31.97	T1N0M0	1
GSM340717	0	37.30	T1N0M0	1
GSM340718	0	36.83	T2aN0M0	1
GSM340719	0	36.57	T1N0M0	1
GSM340720	0	28.10	T2aN0M0	2

GSM340721	1	3.93	T2N0M0	2
GSM340722	1	23.53	T1N0M0	1
GSM340723	1	15.10	T3N0M0	2
GSM340724	0	34.87	TaN0M0	1
GSM340725	0	34.23	T3N1M0	2
GSM340726	0	33.70	TaN0M0	1
GSM340727	1	11.07	T4aN0M0	1
GSM340728	1	5.77	T3N0M0	2
GSM340729	0	33.07	T2N0M0	1
GSM340730	0	32.87	TaN0M0	1
GSM340731	1	9.23	T2N0M0	2
GSM340732	1	7.10	T3N1M0	2
GSM340733	1	17.13	T2N0M0	1
GSM340734	1	23.57	TaN0M0	1
GSM340735	0	30.70	T1N0M0	1
GSM340736	0	30.10	T1N0M0	2
GSM340737	0	29.73	T1N0M0	1
GSM340738	1	15.43	T1N0M0	1
GSM340739	0	29.37	T1N0M0	1
GSM340740	1	17.67	T1N0M0	1
GSM340741	0	28.47	T1N0M0	1
GSM340742	0	23.90	T1N0M0	1
GSM340743	0	23.57	T1N0M0	1
GSM340744	1	13.93	T1N0M0	2
GSM340745	1	2.13	TaN3M1	2
GSM340746	0	22.27	T2N0M0	2
GSM340747	1	6.87	T4N0M1	1
GSM340748	0	20.83	T1N0M0	1
GSM340749	0	21.63	T1N0M0	1
GSM340750	0	21.40	TaN0M0	1
GSM340751	0	57.87	T1N0M0	1
GSM340752	0	21.23	T1N0M0	2
GSM340753	0	21.23	TaN0M0	1
GSM340754	0	20.47	T1N0M0	1
GSM340755	0	20.40	T1N0M0	2
GSM340756	0	19.00	TaN0M0	1
GSM340757	0	79.30	T2N0M0	2
GSM340758	1	15.47	T4N1M0	2
GSM340759	1	26.43	T2N1M1	2
GSM340760	1	13.27	T4N0M0	2
GSM340761	1	6.40	T3NxM0	2
GSM340762	1	10.67	T4aN0M0	2
GSM340763	0	18.13	T2N2M0	1
GSM340764	0	15.37	T3aN0M0	1
GSM340765	0	11.80	T4N2M0	2
GSM340766	0	10.87	T2N0M0	2
GSM340767	0	10.03	T2N0M0	1
GSM340768	0	6.73	T1N0M0	1
GSM340769	0	5.30	T2aN0M0	2

Basic information of GSE48075 cohort

ID	status	survival ti	Tumor stage
GSM1167428	1	65.6	cT2N0M0
GSM1167429	0	37.7	cT3bN0M0
GSM1167433	0	72.4	cT4aN0M0
GSM1167434	1	14.9	cT2N0M0
GSM1167342	1	20.4	pT3bN0M0
GSM1167435	1	18.2	cT3bN0M0
GSM1167436	0	47.7	cT2N0M0
GSM1167437	0	52.2	cT2N0M0
GSM1167439	1	34.6	cT2N0M0
GSM1167441	1	4.3	cT3aN0M0
GSM1167443	1	7.4	cT2N0M0
GSM1167444	0	71.4	cT2N0M0
GSM1167445	1	6.2	cT2N0M0
GSM1167449	0	36.2	cT4aN0M0
GSM1167451	0	27.8	cT2N0M0
GSM1167453	1	18.7	cT4bN0M0
GSM1167454	0	43.2	cT2N0M0
GSM1167455	0	140.7	cT2N0M0
GSM1167457	0	44.6	cT4aN+M+
GSM1167458	0	38.7	cT2N0M0
GSM1167461	1	1.5	cT3bN0M+
GSM1167462	1	1.6	cT2N+M0
GSM1167344	1	68.1	cT2N0M0
GSM1167465	1	8.7	cTis/cT2N0M0
GSM1167466	0	30.4	cT2N0M0
GSM1167468	0	61.1	cT2N0M0
GSM1167469	0	39	cT2N0M0
GSM1167473	1	16.9	cT2N0M0
GSM1167346	1	7.9	cT2N0M0
GSM1167347	1	3.4	cT4bN0M+
GSM1167334	1	37.2	cT2N0M0
GSM1167350	1	23	cT2N0M0
GSM1167354	1	105.9	cT2N0M0
GSM1167360	1	10.5	cT4bN0M0
GSM1167361	0	53.1	cT3bN0M0
GSM1167363	1	14.2	cT3bN0M0
GSM1167335	0	60	cT3bN0M0
GSM1167372	0	42	cT2N0M0
GSM1167373	0	47.8	cT2N0M0
GSM1167375	1	42.8	cT2N0M0
GSM1167376	1	46.3	cT3bN0M0
GSM1167377	0	51.5	cT4aN0M0
GSM1167378	0	41.1	cT2N0M0
GSM1167379	1	4.7	cT4bN0M0
GSM1167336	1	2.9	cT3bN+M+
GSM1167380	1	36.3	cT3bN+M0
GSM1167381	1	8	cT2N0M0
GSM1167383	1	9.3	cT2N0M+
GSM1167384	1	11.8	cT3bN+M0
GSM1167385	1	16	cT3bN0M0
GSM1167387	1	5.9	cT2N+M0
GSM1167388	1	8.1	cT3aN0M0
GSM1167389	0	1.7	cT3bN+M+
GSM1167390	1	25.3	cT3bN0M0
GSM1167391	1	17.4	cT2N+M0
GSM1167393	1	3.4	cT3bN+M0
GSM1167396	1	4.6	cT3bN+M0

GSM1167398	1	6.5	cT2N+M+
GSM1167399	0	179.2	cT2N0M0
GSM1167400	1	86.7	cT2N0M0
GSM1167401	1	8	cT2N0M0
GSM1167402	1	110.2	cT3bN0M0
GSM1167403	1	61	cT2N0M0
GSM1167404	1	82.4	cT3aN0M0
GSM1167406	1	4.9	cT3bN0M0
GSM1167339	1	7.6	cT3bN0M0
GSM1167410	1	28.7	cT2N0M0
GSM1167414	0	109.3	cT2N0M0
GSM1167416	0	9	cT3bN0M0
GSM1167419	0	66.5	cT2N0M0
GSM1167420	0	64.2	cT2N0M0
GSM1167422	0	57.3	cT2N0M0
GSM1167424	0	39.5	cT2N0M0

Basic information of GSE135222 cohort

ID	survival ti	status
GSM3995402	95	1
GSM3995403	618	0
GSM3995404	43	1
GSM3995405	45	1
GSM3995406	412	0
GSM3995407	37	1
GSM3995408	44	1
GSM3995409	168	1
GSM3995410	82	1
GSM3995411	37	1
GSM3995412	279	0
GSM3995413	324	0
GSM3995414	65	1
GSM3995415	59	1
GSM3995416	3	1
GSM3995417	28	1
GSM3995418	257	1
GSM3995419	250	0
GSM3995420	205	0
GSM3995421	25	1
GSM3995422	11	1
GSM3995423	34	1
GSM3995424	73	1
GSM3995425	29	1
GSM3995426	174	1
GSM3995427	23	1
GSM3995428	38	1

Basic information of GSE100797 cohort		
**ID**	**futime**	**fustat**
p01	7.741667	0
p02	0.575	1
p03	0.95	1
p06	0.383333	1
p11	5.958333	0
p14	0.266667	1
p15	5.591667	0
p16	5.441667	0
p17	5.408333	0
p20	1.816667	1
p22	2.675	1
p24	4.675	0
p25	0.458333	1
p26	4.391667	0
p27	0.291667	1
p29	0.45	1
p31	1.675	1
p34	0.416667	1
p35	1.975	1
p36	2.05	1
p37	1.008333	1
p40	1.166667	1
p42	3.525	0
p43	1.9	1
p45	3.1	0
p46	2.066667	1
p47	1.575	1

Basic information of E-MTAB-4321 cohort		
**ID**	**futime**	**fustat**
U0001	2.691667	0
U0002	3.875	0
U0006	3.158333	0
U0007	3.608333	0
U0010	3.341667	0
U0011	3.091667	0
U0012	3.025	0
U0015	1.933333	0
U0018	2.95	0
U0023	2.983333	0
U0024	3.633333	0
U0026	2.983333	0
U0027	2.691667	0
U0028	0.891667	0
U0032	3.025	0
U0035	3.466667	0
U0036	2.75	0
U0037	3.016667	0
U0038	3.458333	0
U0039	3.225	0
U0041	3.391667	0
U0042	3.766667	0
U0045	1.55	0
U0046	2.858333	0
U0048	3.208333	0
U0053	2.525	0
U0054	1.066667	0
U0057	3.25	0
U0059	2.991667	0
U0060	2.425	0
U0062	1.091667	0
U0064	1.883333	0
U0067	2.666667	0
U0069	1.825	0
U0071	3.408333	0
U0072	3.125	0
U0073	3.266667	0
U0074	3.166667	0
U0076	3.1	0
U0077	3.35	0
U0078	3.566667	0
U0080	3.333333	0
U0083	3.45	0
U0086	3.341667	0
U0087	3.366667	0
U0088	3.008333	0
U0089	2.725	0
U0090	2.966667	0
U0092	2.058333	0
U0094	2.975	0
U0095	2.541667	0
U0099	1.958333	0
U0100	2.383333	0
U0102	2.5	0
U0104	1.15	0
U0105	3.475	0
U0106	3	0

U0109	1.983333	0
U0110	2.975	0
U0114	3.183333	0
U0115	3.566667	0
U0117	2.75	0
U0118	3.433333	0
U0119	1.991667	0
U0120	2.241667	0
U0121	2.491667	0
U0122	2.5	0
U0125	1.633333	0
U0126	2.45	0
U0128	2.691667	0
U0129	2.458333	0
U0130	2.5	0
U0134	4.058333	0
U0138	0.075	0
U0139	0.075	0
U0140	3.375	0
U0141	3.45	0
U0142	3.891667	0
U0143	1.258333	0
U0144	3.875	0
U0147	3.525	0
U0149	3.525	0
U0150	0.066667	0
U0151	2.9	0
U0152	0.125	0
U0153	3.558333	0
U0154	3.575	0
U0156	3.416667	0
U0158	3.658333	0
U0159	3.791667	0
U0160	3.758333	0
U0161	3.416667	0
U0168	0.133333	0
U0169	0.091667	0
U0170	0.116667	0
U0171	3.375	0
U0175	0	0
U0177	1.058333	0
U0178	0	0
U0179	3.066667	0
U0181	1.475	0
U0182	3.191667	0
U0183	3.191667	0
U0184	3.141667	0
U0186	0	0
U0188	2.766667	0
U0189	2.191667	0
U0190	2.975	0
U0193	0	0
U0195	3.058333	0
U0199	6.116667	0
U0200	4.916667	0
U0201	5.291667	0
U0204	5.05	0
U0207	0.466667	0
U0214	2.316667	0

U0219	3.416667	0
U0224	5.808333	0
U0226	2.516667	0
U0233	5.625	0
U0261	5.183333	0
U0262	6.241667	0
U0264	5.916667	0
U0268	5.75	0
U0273	5.6	0
U0275	5.433333	0
U0278	5.808333	0
U0283	5.158333	0
U0290	5.891667	0
U0307	4.991667	0
U0316	4.858333	0
U0317	5.441667	0
U0322	5.658333	0
U0323	5.408333	0
U0326	0.533333	0
U0328	6.075	0
U0329	5.25	0
U0330	4.058333	0
U0332	4.9	0
U0334	5.791667	0
U0337	5.083333	0
U0344	4.333333	0
U0346	5.183333	0
U0348	3.966667	0
U0349	5.116667	0
U0350	0	0
U0378	3.491667	0
U0380	5.483333	0
U0383	1.883333	0
U0384	2.666667	0
U0385	1.966667	0
U0392	4.733333	0
U0394	5.375	0
U0397	5.083333	0
U0399	0	0
U0400	5.125	0
U0401	5.366667	0
U0411	3.033333	0
U0412	2.875	0
U0414	4.808333	0
U0416	5.191667	0
U0421	1.516667	0
U0425	4.25	0
U0426	5.233333	0
U0427	4.908333	0
U0430	4.908333	0
U0433	2.008333	0
U0435	4.808333	0
U0436	5.616667	0
U0437	4.925	0
U0438	4.45	0
U0439	4.841667	0
U0442	5.383333	0
U0446	5.3	0
U0447	5.283333	0

U0448	1.683333	0
U0450	1.316667	0
U0453	5.025	0
U0454	4.916667	0
U0455	5.116667	0
U0457	5.283333	0
U0458	5.65	0
U0459	4.95	0
U0461	5.233333	0
U0466	3.208333	0
U0469	4.825	0
U0473	4.825	0
U0475	3.391667	0
U0476	4.675	0
U0479	0.158333	0
U0494	4.725	0
U0496	2.975	0
U0497	5.05	0
U0502	3.908333	0
U0516	1.891667	0
U0519	1.258333	0
U0531	3.908333	0
U0534	3.641667	0
U0535	3.65	0
U0538	3.625	0
U0541	3.608333	0
U0542	3.608333	0
U0544	2.225	0
U0549	3.591667	0
U0550	3.591667	0
U0551	1.5	0
U0553	3.508333	0
U0555	1.608333	0
U0556	3.508333	0
U0558	3.491667	0
U0559	2.116667	0
U0560	3.45	0
U0562	2.916667	0
U0566	3.1	0
U0570	1.383333	0
U0573	3.341667	0
U0577	2.05	0
U0580	0.166667	0
U0583	3.391667	0
U0584	3.166667	0
U0585	3.358333	0
U0587	1.983333	0
U0589	2.541667	0
U0590	3.6	0
U0598	2.716667	0
U0600	4.166667	0
U0603	2.241667	0
U0605	4.025	0
U0606	3.366667	0
U0607	4.066667	0
U0608	3.416667	0
U0611	3.183333	0
U0612	3.841667	0
U0613	1.441667	0

U0614	3.908333	0
U0615	3.125	0
U0618	3.183333	0
U0624	2.4	0
U0625	3.358333	0
U0631	3.316667	0
U0635	2.683333	0
U0638	3.191667	0
U0641	3.191667	0
U0642	1.825	0
U0643	3.675	0
U0646	3.566667	0
U0648	3.7	0
U0654	3.341667	0
U0655	2.266667	0
U0657	3.475	0
U0664	3.858333	0
U0666	2.7	0
U0670	1.341667	0
U0754	3.35	0
U0765	1.891667	0
U0766	2	0
U0768	4	0
U0772	3.533333	0
U0777	3.35	0
U0779	3.325	0
U0794	4.208333	0
U0796	0	0
U0832	0.891667	0
U0835	0	0
U0839	1.516667	0
U0844	3.483333	0
U0847	2.683333	0
U0850	2.508333	0
U0855	1.525	0
U0856	3.016667	0
U0867	4.233333	0
U0870	4.05	0
U0903	1.283333	0
U0904	2.808333	0
U0905	2.133333	0
U0907	4.7	0
U0909	5.333333	0
U0910	4.233333	0
U0912	3.525	0
U0913	4.783333	0
U0915	3.741667	0
U0918	3.941667	0
U0928	3.95	0
U0932	4.083333	0
U0952	3.133333	0
U0954	4.691667	0
U0963	4.2	0
U0965	4.616667	0
U0972	4.575	0
U0973	3.683333	0
U0975	1.416667	0
U0976	4.008333	0
U0977	4.416667	0

U0986	4.208333	0
U0998	4.366667	0
U0999	4.266667	0
U1002	2.916667	0
U1005	3.35	0
U1006	1.225	0
U1011	1.133333	0
U1018	2	0
U1019	2.258333	0
U1020	2.591667	0
U1023	0.866667	0
U1025	3.35	0
U1026	1.4	0
U1030	2.641667	0
U1031	1.8	0
U1032	4.508333	0
U1033	4.641667	0
U1037	1.058333	0
U1043	2.4	0
U1046	1.008333	0
U1050	2.641667	0
U1052	2.208333	0
U1055	1.183333	0
U1057	3.183333	0
U1061	0	0
U1064	2.216667	0
U1067	2.583333	0
U1082	2.216667	0
U1084	3.491667	0
U1089	0	0
U1099	2.4	0
U1100	2.883333	0
U1103	2.125	0
U1105	2.491667	0
U1106	2.65	0
U1108	2.658333	0
U1113	1.816667	0
U1123	1.591667	0
U1135	1.4	0
U1136	1.875	0
U1146	2.325	0
U1148	2.283333	0
U1150	2.266667	0
U1151	0	0
U1153	2.691667	0
U1155	0	0
U1157	2.608333	0
U1158	0	0
U1161	2.416667	0
U1162	1.233333	0
U1165	2.583333	0
U1174	3.008333	0
U1177	2.5	0
U1179	2.966667	0
U1182	2.9	0
U1188	0.358333	0
U1189	3.175	0
U1190	1.933333	0
U1191	0	0

U1192	0.7	0
U1194	2.133333	0
U1196	2.341667	0
U1205	3.333333	0
U1206	2.175	0
U1207	2.666667	0
U1209	2.616667	0
U1211	4.425	0
U1212	1.583333	0
U1228	2.975	0
U1230	3.316667	0
U1231	2.333333	0
U1232	2.708333	0
U1233	3.075	0
U1235	2.65	0
U1237	2.816667	0
U1240	2.866667	0
U1242	1.425	0
U1243	2.525	0
U1244	1.075	0
U1248	3.425	0
U1249	1.125	0
U1256	1.191667	0
U1264	2.491667	0
U1265	2.433333	0
U1266	2.366667	0
U1267	2.691667	0
U1270	2.508333	0
U1271	2.025	0
U1272	2.625	0
U1275	1.791667	0
U1276	2.508333	0
U1277	1.975	0
U1279	2.175	0
U1280	2.308333	0
U1282	2.358333	0
U1284	2.291667	0
U1286	2.283333	0
U1288	2.141667	0
U1289	2.166667	0
U1291	2.016667	0
U1292	1.908333	0
U1293	2.225	0
U1295	1.966667	0
U1297	1.15	0
U1298	1.816667	0
U1300	2.258333	0
U1301	1.975	0
U1303	2.191667	0
U1304	2.175	0
U1305	2.3	0
U1307	1.808333	0
U1308	1.908333	0
U1309	2.383333	0
U1311	2.316667	0
U1314	2.9	0
U1317	2.808333	0
U1330	2.075	0
U1332	2.025	0

U1335	2.025	0
U1336	2.058333	0
U1337	2.058333	0
U1338	2.05	0
U1341	2.3	0
U1343	2.233333	0
U1345	2.075	0
U1346	2.108333	0
U1349	2.216667	0
U1354	1.95	0
U1356	1.291667	0
U1363	1.975	0
U1368	2	0
U1374	2.191667	0
U1388	3.033333	0
U1403	2.741667	0
U1404	2.183333	0
U1408	1.208333	0
U1409	2.35	0
U1412	0.883333	0
U1413	2.541667	0
U1425	2.25	0
U1426	0.85	0
U1427	2.25	0
U1430	3.266667	0
U1431	2.591667	0
U1432	2.333333	0
U1434	2.85	0
U1435	2.641667	0
U1458	0.541667	0
U1462	0.7	0
U1464	0.716667	0
U1476	1.091667	0
U1478	1.083333	0
U0013	1.275	1
U0091	1.166667	1
U0093	0.433333	1
U0137	1.475	1
U0256	2.083333	1
U0267	0.35	1
U0291	2.933333	1
U0312	0.766667	1
U0408	0.516667	1
U0431	0.783333	1
U0434	0.266667	1
U0512	0.108333	1
U0514	0.091667	1
U0515	0.075	1
U0623	2.175	1
U0627	0.116667	1
U0645	0.25	1
U0653	0.083333	1
U0659	1.216667	1
U0696	1.2	1
U0706	0.15	1
U0709	0.3	1
U0989	1.075	1
U0993	3.658333	1
U1171	0.266667	1

U1263	2.525	1
U1283	1.55	1
U1396	0.166667	1
U1415	0.108333	1
U1465	0.083333	1
U1480	0.725	1

Basic information of Nathanson2017pre cohort		
**ID**	**futime**	**fustat**
P6126	1.9	0
P167	2.7	0
P1494	2	0
P2056	3.9	0
P2051	0.8	1
P5784	0.5	1
P8727	0.4	1
P1867	1.5	1
P3549	0.5	1
